# STEM-PD trial protocol: a multi-centre, single-arm, first-in-human, dose-escalation trial, investigating the safety and tolerability of intraputamenal transplantation of human embryonic stem cell-derived dopaminergic cells for Parkinson’s disease

**DOI:** 10.1136/bmjopen-2025-107597

**Published:** 2025-12-30

**Authors:** Gesine Paul, Hjalmar Bjartmarz, Anders Björklund, Emma Cutting, Amy Evans, Bronwen Harry, Oskar Hansson, Saeed Kayhanian, Agnete Kirkeby, Nicholas Lao-Kim, Olle Lindvall, Jenny Nelander, Paola Piccini, Ruben Smith, Susann Ullén, Trinette Van Vliet, Hakan Widner, Malin Parmar, Roger A Barker

**Affiliations:** 1 Department of Neurology, Skåne University Hospital, Malmö, Sweden; 2 Department of Clinical Sciences and Wallenberg Center for Molecular Medicine, Lund University, Lund, Sweden; 3 Department of Neurosurgery, Region Skåne, Kristianstad, Sweden; 4 Department of Experimental Medical Science, Wallenberg Neuroscience Center and Lund Stem Cell Center, Lund, Sweden; 5 Cambridge Clinical Trials Unit, Cambridge, UK; 6 Department of Clinical Neurosciences, University of Cambridge, Cambridge, UK; 7 Lund University; Skane University Hospital, Malmö, Sweden; 8 Cambridge Stem Cell Institute, Cambridge, UK; 9 Department of Experimental Medical Science, Lund University, Lund, Sweden; 10 Novo Nordisk Foundation Center for Stem Cell Medicine (reNEW), Department of Biomedical Sciences, University of Copenhagen, Copenhagen, Denmark; 11 Imperial College, London, UK; 12 Lund Stem Cell Center and Department of Clinical Sciences, Lund, Sweden; 13 Department of Neurology, Imperial College London, Imperial College Healthcare NHS Trust, London, UK; 14 Clinical Studies Sweden - Forum South, Skåne University Hospital, Lund, Sweden

**Keywords:** Clinical Trial, Parkinson-s disease, Neurosurgery

## Abstract

**ABSTRACT:**

**Introduction:**

Parkinson’s disease (PD) is a common neurodegenerative disease, which has extensive pathology that critically includes the loss of midbrain dopaminergic neurons. This loss leads to debilitating motor features such as bradykinesia and rigidity, as well as some non-motor symptoms. Intracerebral dopamine cell transplants have been explored for many years as a new approach to treating PD and initially used human fetal ventral mesencephalic tissue with inconsistent results, related in part to major logistical challenges in sourcing enough tissue of the right quality and the limited possibilities for quality control and standardisation. Dopaminergic neurons can now be derived reliably from human stem cell sources, which may overcome some of the challenges associated with fetal tissue transplantations.

**Methods and analysis:**

STEM-PD is a multi-centre, single-arm, dose-escalation, first-in-human advanced therapy investigational medicinal product (ATIMP) trial in Europe using a cell product that consists of dopaminergic neural progenitors derived from the RC17 human embryonic stem cell line. The aim of the study is to assess the safety, tolerability and feasibility of intraputamenal transplantation of this cell product in patients with moderately advanced PD. Eight participants will be recruited from two sites, Skånes University Hospital (Lund, Sweden) and Cambridge University Hospital (Cambridge, UK). The primary outcome of the trial is safety and tolerability, assessed by the number and nature of adverse events and serious adverse events, and the absence of space-occupying lesions on cranial MRI, in the first 12 months following transplantation. Secondary and exploratory outcomes, including clinical measures, changes in anti-Parkinson’s medication and measures of graft survival using positron emission tomography imaging, will be assessed at both 12 and 36 months post-grafting.

**Ethics and dissemination:**

Ethical approval was obtained from the Swedish Ethical Review Authority (EPM dnr 2021-06945-01) and South Central - Oxford A Research Ethics Committee (reference 23/SC/0243). Clinical Trial Authorisation was given by the Swedish Medical Products Agency (Dnr: 5.1-2022-57953) and the Medicines and Healthcare products Regulatory Agency for clinical trials authorisation (reference CTA 40773/0001/001-0001). Authorisation for transfer to Clinical Trial Regulation (EU) 536/2014 was given by the Swedish Medical Products Agency (Dnr: 5.1.1-2024-100773). Potential participants will receive verbal and written information about the trial and written informed consent will be obtained prior to enrolment. A lay summary of the results of the trial will be uploaded to the trial website which is publicly accessible. Trial results will be published in peer-reviewed journals.

**Trial registration numbers:**

NCT05635409.

**STRENGTHS AND LIMITATIONS OF THIS STUDY**

- First-in-human study to investigate the safety and tolerability of transplanting RC17 human embryonic stem cell-derived dopaminergic precursor cells into the brains of patients with Parkinson’s disease.
- A trial working across two centres in different countries to show feasibility across sites.
- The trial involves a single dose escalation after assessment of 6 months post-transplant safety data from a lower-dose cohort of four patients.
- Recruitment to trial from a previous long-running longitudinal observational trial (TransEuro), providing individualised disease trajectories for comparison pre- and post-grafting.
- The trial’s limitations are the small number of participants and the relatively short follow-up period in the first instance.

## Introduction

 Parkinson’s disease (PD) is a common neurodegenerative disorder that is characterised by the loss of midbrain dopamine (DA) neurons from the substantia nigra.^1^ These neurons modulate movement and certain aspects of cognition and therefore their loss leads to motor disabilities including bradykinesia and rigidity, as well as other motor and non-motor problems.^2^ There are currently no disease-modifying treatments for PD, and medical management is mainly focused on controlling the motor features using drugs that act on the dopaminergic system, such as levodopa (L-dopa) or DA agonists.^3^ Over time, these drugs become less effective and are associated with significant side-effects, including motor fluctuations, and for this, a number of other approaches have been used and are now in routine clinical practice (eg, deep brain stimulation (DBS) and dopaminergic infusion therapies).^4^ In addition, there is a significant non-nigral and non-dopaminergic pathology of PD which can significantly impact on quality of life.^5^ Thus, there is an urgent need for better treatments of Parkinson’s and a number of experimental approaches are being considered, including gene therapies, immune therapies, small molecules and drug repurposing approaches.^4^

One of the most promising potential PD therapies as an alternative to oral or enteral DA replacement medications has been DA cell replacement, using cells obtained from the developing human foetal midbrain.^6^ This approach has given inconsistent results, with some patients doing extremely well, exhibiting near-normal graft-derived dopaminergic innervation of the putamen and coming off all their anti-PD medication for years, while others have shown no or only modest clinical improvements.^7 8^ In some cases, patients also developed new involuntary movements called graft-induced dyskinesias (GIDs), which were so severe in a few patients that further neurosurgery with DBS was needed.^9^,^11^ These GIDs were thought to arise from the transplantation of serotonin neurons that develop adjacent to the DA cells in the human brainstem and that cannot be completely excluded in the dissection and tissue preparation.^12^ The inherent variation of cell quality using human fetal tissue also makes it impossible to properly quality control the product for grafting, as each patient will ultimately have a tissue implant derived from a unique collection of foetal material.^13^ Additionally, it is very challenging to source enough tissue for transplantation, as exemplified in the recent TransEuro trial of fetal tissue grafting for PD, in which 21 transplant surgeries were performed while 87 scheduled surgery slots were cancelled due to a lack of available tissue.^13^

To address these issues, a human stem cell-derived dopaminergic neural progenitor cell (the STEM-PD product) has been developed from the RC17 human embryonic stem cell line, which we will investigate as a DA replacement therapy through grafting into patients with moderately advanced PD in the STEM-PD clinical trial.^14^ This is the first such trial using this approach in Europe and the first to use this human embryonic stem cell line, but a similar approach has been undertaken by other groups who have recently published the results from their first-in-human trials.^15 16^

## Methods and analysis

### Overview

The STEM-PD trial is a multi-centre, single-arm, dose-escalation, first-in-human advanced therapy investigational medicinal product (ATIMP) trial. The aim of the study is to assess the safety, tolerability and feasibility of intraputamenal transplantation of the STEM-PD product in patients with moderately advanced PD.

Participants will be patients with moderately advanced PD who have been followed up for at least 12 months as part of the TransEuro observational study and who meet all inclusion and exclusion criteria as outlined in table 1^13^.

**Table 1:** Inclusion and exclusion criteria for the trial

Inclusion criteria	Exclusion criteria
Have given written informed consent to participate in the trial	Tremor dominant disease, as assessed by the PI or other delegated clinician
Diagnosed with PD as defined using Queen Square Brain Bank criteria	Significant drug-induced dyskinesias as defined by a score of ≥2 in the Abnormal Involuntary Movement Scale (AIMS) dyskinesias rating scale, in any body part in the ON state
Moderate disease as defined by having Hoehn and Yahr stage 2–3 in OFF state	Ongoing major medical or psychiatric disorders, including depression (MADRS>20) and psychosis, or other medical unsuitability, as judged by the PI or other delegated clinician
Disease duration >10 years	Any contraindication to neurosurgery
Male or female, aged between 50 and 75 years (inclusive)	Unable to be imaged using MRI
Have a significant response to dopamine therapies as judged by the PI or other delegated clinician	Extensive ventral striatal loss or normal findings on F-DOPA PET at screening
Have symptoms that are not appropriately controlled by existing oral anti-PD medications, as judged by the PI or other delegated clinician	Significant cognitive impairment indicative of an incipient dementia/established dementia or values consistent with MoCA score of ≤24
Ability to travel to Lund for surgery	Unable to perform normal copying of interlocking pentagons and/or a semantic fluency score for naming animals of less than 20 over 90 s
Followed up for at least 12 months prior to inclusion in this trial in the TransEuro observational study	
Be fluent in English/Swedish to enable completion of questionnaires as assessed by the PI or other delegated clinician at Cambridge/Lund, respectively	
Be approved by the TMG clinical subgroup for trial participation	

F-DOPA, [18F]-fluorodopa; MADRS, Montgomery-Åsberg Depression Rating Scale; MoCA, Montreal Cognitive Assessment; PD, Parkinson’s disease; PET, positron emission tomography; PI, principal investigator; TMG, Trial Management Group.

Eight participants will be recruited, split into two equal cohorts (n=4), with the aim to graft 100 000 surviving DA neurons per putamen in the first cohort (3.54×10^6^ STEM-PD cells grafted per putamen) and 200 000 surviving DA neurons per putamen in the second cohort (7.08×10^6^ STEM-PD cells grafted per putamen).

The number of cells being grafted is based on studies suggesting these are the numbers of DA cells that need to differentiate and survive for a meaningful clinical benefit in PD.

The decision for the escalation of dose for cohort 2 will be undertaken once all four participants in the dose 1 group have reached the 6-month follow-up time point. The data relating to clinical evaluations, MRI scans and their 6 months [^18^F]-fluorodopa (F-DOPA) Positron emission tomography (PET) imaging will be reviewed by the Data and Safety Monitoring Board (DSMB) in conjunction with the clinical subgroup of the Trial Management Group (TMG). The DSMB will make a recommendation on how to proceed, and a decision will be made by the TMG around the progression to dose 2. The DSMB can also recommend waiting for additional data (eg, up to the 12-month follow-up with imaging) before making any recommendation.

Participants will be recruited from two sites—Lund, Sweden and Cambridge, UK, with all surgeries occurring in Lund using the same surgical device. Transplantation of the cells will be done bilaterally in a single session in a stereotactic neurosurgical procedure, using the Rehncrona-Legradi device for cell delivery into the putamen. This device is manufactured in-house at the Skåne University Hospital in Lund and is not CE marked, so it can only be used in this hospital (for more detail, see Barker *et al*^17^). This device has been shown to deliver developing DA cells accurately to the target site—the putamen^18^—and so we deemed it to be the best device to use in this trial given no approved and licensed device is available for cell delivery to the CNS. Given this, all UK participants will have to travel to Sweden and, to help support this, they will be accompanied by a caregiver and a medically qualified member of the UK trial team. Furthermore, their stage of disease has been chosen (after discussions with the ethical committees) such that they are fully ambulant when on medication, so they will be able to undertake this travel. This is part of the inclusion criteria.

The surgical procedure will use a stereotactic image-guided technique. Five targets for each putamen will be used, two in the anterior part and three in the posterior part of the putamen, divided as follows: one anterior to the anterior commissure; one at the anterior commissural line; and three posterior to the anterior commissure. The trajectories to those targets for each putamen will be made through one or more burr holes per side of the cranium, depending on the best trajectory to the target, and avoiding the ventricles and blood vessels, which will be imaged preoperatively using MRI. The trajectories will be placed to achieve maximal distribution of the graft tissue within the putamen. For dose 1, each tract will have four deposits, and for dose 2, each tract will have eight deposits. Each deposit will contain 2.5 µL of cell suspension (70 000 cells/µL).

The trial does not have any predetermined stopping criteria. However, premature termination would be triggered by concerns raised by the investigators and/or the DSMB regarding safety and ethical implications of continuing the trial. A temporary halt to further transplantations will be triggered if one participant develops a serious adverse event (SAE) judged to be possibly, probably or definitely related to the STEM-PD product, or if two participants develop any adverse reaction towards the cell product that is graded as severe. In the case of a temporary halt, all investigators, the DSMB and trial sites will be notified promptly. Regulatory authorities and ethics committees will be informed of the temporary halt within 15 days via a substantial amendment. If the trial is temporarily halted, the trial teams in Cambridge and Lund, and a representative of the Sponsor will need to make a formal decision to restart the trial, providing justification to do so. The DSMB will also be consulted prior to making this decision and will be asked to provide a recommendation on how to proceed.

### Patient and public involvement

An advisory panel comprised of five people with PD and carers reviewed and provided feedback on the research question, trial design and outcomes, and participant pathway for this research protocol. The advisory panel also reviewed the participant information sheets for clarity to ensure they were detailed enough and easy to understand, and a member of the panel attended the ethical committee meeting.

### Non-investigational medicinal products

The STEM-PD product is an allogenic cell product, and participants will therefore receive an immunosuppressant regimen for 12 months, with the standard agents used in solid organ transplantation regimes. The following agents will be used for all patients after surgery: tacrolimus (0.05 mg/kg two times per day), azathioprine (1 mg/kg once daily) and prednisolone (40 mg once daily for 12 weeks, following which it will be tapered to 10 mg daily). Additionally, participants will receive methylprednisolone (1 g intravenous) at the time of surgery and basiliximab (20 mg intravenous) on the day of surgery and on day 4 following transplantation. Prophylactic antibiotics will be administered around the time of surgery according to local guidelines, as well as trimethoprim-sulfamethoxazole three times per week for the duration of immunosuppression. Prophylaxis for osteoporosis with alendronic acid and calcium carbonate/vitamin D supplementation will also be administered for the duration of immunosuppression, as well as gastroprotection with a proton-pump inhibitor.

### Schedule of assessments

**Figure 1:**
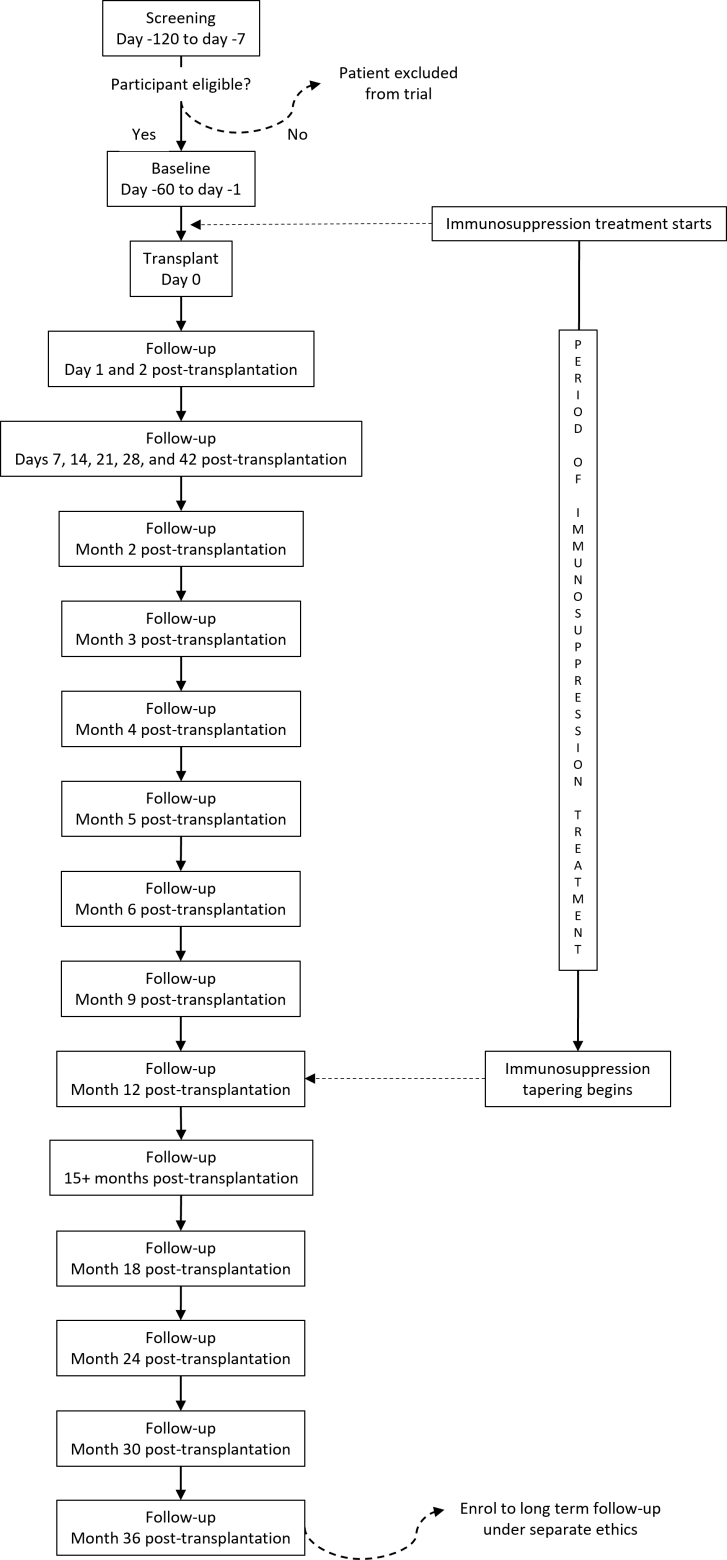
Flowchart of trial timeline.

Participants will be followed up for 36 months following transplantation (figure 1). Preoperative assessments will include CT-angiography and MRI for surgical planning. Assessments of safety will be undertaken with a clinical examination and blood tests at each visit, with additional MRI brain imaging being undertaken at day 28, month 2, month 3, month 6, month 9, month 12, month 24 and month 36 following surgery.

PET imaging will be undertaken at screening ([^18^F]F-DOPA), baseline ([^18^F]FE-PE2i) and then month 6 ([^18^F]F-DOPA), month 12 ([^18^F]F-DOPA and [^18^F]FE-PE2i), month 24 ([^18^F]F-DOPA and [^18^F]FE-PE2i), and month 36 ([^18^F]F-DOPA and [^18^F]FE-PE2i) following surgery.

Major motor and cognitive assessments in both ON and OFF DA medication states will be undertaken at baseline, month 6, month 12, month 24 and month 36 following surgery.

The schedule of assessments is summarised in table 2.

**Table 2:** Schedule of assessments over the course of the trial

Assessment	Screening	Baseline	Day before surgery	Surgery day 0	Days							Months												
Visit	Day −120 to day −7	Day −60 to day −1			1	2	7	14	21	28	42	2	3	4	5	6	9	12	15+*	18	24	30	36	
**General**																								
Informed consent	X																							
Assessment of eligibility	X																							
Concomitant medication (including PD medication)	X	X	X	X	X	X	X	X	X	X	X	X	X	X	X	X	X	X	X	X	X	X	X	
Height	X																							
Weight	X	X	X				X	X	X	X	X	X	X	X	X	X	X	X	X					
ECG		X				X																	X	
Vital signs		X		X	X	X	X	X	X	X	X	X	X	X	X	X	X	X	X	X	X	X	X	
Physical examination	X	X					X	X	X	X	X	X	X	X	X	X	X	X		X	X	X	X	
Demographics	X																							
Medical history	X																							
Years of education	X																							
Smoking status	X																							
AE assessment	X	X		X	X	X	X	X	X	X	X	X	X	X	X	X	X	X	X	X	X	X	X	
Pregnancy test†	X	X	X							X		X	X	X	X	X	X	X		X				
Provision of participant contact/alert card		X																						
Review nIMP compliance							X	X	X	X	X	X	X	X	X	X	X	X	X	X				
Enrolment to long-term F/U																							X	
**Biological samples**																								
FBC	X		X‡		X	X	X	X	X	X	X	X	X	X	X	X	X	X						
U&E	X		X‡		X	X	X	X	X	X	X	X	X	X	X	X	X	X						
Tacrolimus/ciclosporin level§					X	X	X	X	X	X	X	X	X	X	X	X	X	X						
LFTs	X		X‡		X	X	X	X	X	X	X	X	X	X	X	X	X	X						
Coagulation tests	X		X‡																					
CRP	X		X‡		X	X	X	X	X	X	X	X	X	X	X	X	X	X						
Cross-match testing			X‡																					
Microbiology/serology	X																							
HLA antibody titres¶	X												X			X	X	X		X	X	X	X	
Glucose	X		X‡		X	X	X	X	X	X	X	X	X	X	X	X	X	X		X				
HbA1c	X		X‡										X			X	X	X		X				
Blood lipids	X		X‡		X		X	X		X		X	X			X	X	X		X				
TPMT	X																							
MRSA (Cambridge participants only)	X																							
Research blood sample (optional)		X														X				X			X	
Lumbar puncture for CSF collection and accompanying blood sample** (optional)		X														X				X				
**Imaging**																								
MRI (screening)	X																							
MRI (surgical planning)		X																						
CT angiogram		X																						
MRI (with frame)				X																				
MRI (safety)						X				X		X	X			X††	X	X††			X††		X††	
MRI (extended battery)		X														X‡‡		X‡‡			X‡‡		X	
PET scan (F-DOPA)§§	X															X‡‡		X			X‡‡		X	
Done (PE2i)§§		X																X‡‡			X‡‡		X	
MDS-UPDRS part III¶¶	X ON+OFF	X ON+OFF											X ON			X ON+OFF	X ON	X ON+OFF		X ON	X ON+OFF	X ON	X ON+OFF	
MDS-UPDRS parts I, II and intravenous	X	X											X			X	X	X		X	X	X	X	
9 hole peg test¶¶		X ON+OFF											X ON			X ON+OFF	X ON	X ON+OFF		X ON	X ON+OFF	X ON	X ON+OFF	
Timed sit-stand-walk test¶¶		X ON+OFF											X ON			X ON+OFF	X ON	X ON+OFF		X ON	X ON+OFF	X ON	X ON+OFF	
Hauser patient diary card***		X											X			X	X	X		X	X	X	X	
RUSH dyskinesia scale¶¶		X ON+OFF											X ON			X ON+OFF	X ON	X ON+OFF		X ON	X ON+OFF	X ON	X ON+OFF	
AIMS¶¶	X ON+OFF	X ON+OFF											X ON			X ON+OFF	X ON	X ON+OFF		X ON	X ON+OFF	X ON	X ON+OFF	
30 s tap test¶¶		X ON+OFF											X ON			X ON+OFF	X ON	X ON+OFF		X ON	X ON+OFF	X ON	X ON+OFF	
PKG***		X											X			X	X	X		X	X	X	X	
L-dopa challenge test¶¶		X														X		X			X		X	
**Cognitive assessments**																								
MoCA	X ON															X ON		X ON			X ON		X ON	
Pentagon copying	X ON																							
Semantic (animal naming) fluency†††	X ON																							
HVLT-R		X ON																					X ON	
Stroop test		X ON																					X ON	
Digit span test		X ON																					X ON	
WAIS similarities test		X ON																					X ON	
Boston naming task		X ON																					X ON	
**Psychiatric assessments**																								
MADRS	X	X								X			X			X		X			X		X	
Non-motor/QOL assessments																								
PDQ-39		X																	X			X		X
EQ-5D-5L		X																	X			X		X
PD NMSS		X																	X			X		X
TREATMENT																								
Surgery (transplantation of STEM-PD product)				X																				
nIMPs			As prescribed (including tapering)																					

‘ON’ or ‘OFF’ dopaminergic medications are indicated for clinical assessments at each stage.

* The 15+ month visit will occur within 2 weeks of the participant completing their course of immunosuppressants. If the completion of immunosuppressants is within 2 weeks of the scheduled 18-month post-surgery visit, then this visit and the assessments listed will not take place.

† To be performed for female participants of childbearing potential only. At screening, a serum pregnancy test will be performed, and on all subsequent visits, either a serum or urine pregnancy test will be performed at the site’s discretion. Monthly urine pregnancy tests will be performed by participants at home at months 7, 8, 10, 11, 13, 14, 15, 16 and 17. A member of the trial team will contact the participant to confirm the pregnancy test has been performed and record the result.

‡ Samples will not need to be repeated on day −1 if performed within the 3 days prior to this visit, ie, to day −4). Blood typing does not need to be repeated.

§ To be performed depending on which immunosuppressant treatment the participant is on, that is, if the participant is on tacrolimus treatment, then the tacrolimus level will be tested, and if on ciclosporin A, the CyA level will be tested.

¶ At screening, will include a screening test of anti-HLA antibodies. If positive, a specific assessment of any major histocompatibility complex (MHC) class I antigens against those expressed in the STEM-PD product will be carried out. For all other visits, HLA antibodies will be monitored.

** Where possible, blood analysis will be performed on samples already collected.

†† For visits completed in Lund, this will be done simultaneously with the extended battery MRI

‡‡ Optional for Cambridge participants.

§§ Low dose CT scans will be performed in conjunction with each positron emission tomography scan performed at RS-SUS.

¶¶ To be videoed with the participant wearing a cap.

*** Hauser patient diary cards and PKG will be provided to participants ahead of the visit to enable completion of data prior to the visit. At the visits, the trial team will collect in the Hauser patient diary cards and PKG, as outlined in STEM-PD SOP4: Motor and Cognitive Assessments. PKG for Lund participants only.

††† The semantic (animal naming) fluency assessment may also be videoed if required by the site to assist with scoring.

AE, adverse event; CRP, C reactive protein; CSF, cerebrospinal fluid; EQ-5D-5L, Euroqol questionnaire; FBC, full blood count; F-DOPA, [18F]-fluorodopa; F/U, follow-up; HbA1c, glycated haemoglobin; HLA, human leucocyte antigen; HVLT-R, Hopkins Verbal Learning Test - Revised; LFT, liver function tests; MADRS, Montgomery-Åsberg Depression Rating Scale; MoCA, Montreal Cognitive Assessment; MRSA, Methicillin-resistant Staphylococcus aureus; nIMP, non-investigational medicinal product; NMSS, Non-Motor Symptoms Scale; PD, Parkinson’s disease; PDQ-39, Parkinson's disease Questionnaire 39; PKG, Parkinson’s KinetiGraph; QOL, quality of life; TPMT, hiopurine methyltransferase; U&E, urea and electrolytes; WAIS, Wechsler Adult Intelligence Scale.

### Assessments to be undertaken

Occurrence of adverse events (AEs) and SAEs will be recorded as is standard in all clinical trials. AEs of special interest will also be collected and include:

1. Stroke.
2. Major intracranial haemorrhage.
3. Central nervous system infection.
4. Major worsening of the participant’s PD or rapid and substantial worsening of their existing dyskinesias that does not respond to a reduction in dopaminergic therapy.
5. Clinically significant event which may be related to immunosuppressive treatment, such as atypical infections, malignancies (squamous skin cancer, Kaposi’s sarcoma and lymphomas) and metabolic events (osteoporosis, diabetes).
6. Any event occurring up to 24 hours post-transplantation (not including well-known surgery-related events, eg, nausea, malaise).

Clinical assessments will be undertaken to evaluate changes between baseline and month 36 post-transplantation in four domains: global cognitive measures, non-motor/quality of life assessments, motor features in the OFF DA medication state and PD medications. The assessments to be undertaken are summarised in table 3. The motor assessments will be video recorded, such that they can be rated by a blinded independent rater, as was done in the TransEuro fetal DA cell transplant trial.^17^

**Table 3:** Clinical assessments to be undertaken in four domains at baseline and month 36 following transplantation

Global cognitive changes	Non-motor/quality of life assessments	Motor features in the OFF dopamine medication state	Parkinson’s disease medications
Montreal Cognitive Assessment	PDQ-39: overall total+subscale totals	MDS-UPDRS part III: total score	L-dopa equivalent dose
Hopkins Verbal Learning Test-Revised	EQ-5D-5L VAS score	AIMS: total score+dichotomous >2 points in one body part	L-dopa yes/no
Stroop	EQ-5D-5L: individual scores for each dimension	9 hole peg test: separately for each side, mean of two rounds	
Digit span	NMSS: overall total+total score per domain	Timed sit-stand-walk: one value (seconds)	
Wechsler Adult Intelligence Scale similarities	MADRS score: total score at baseline and 36 months	Hauser diary card: percentage of waking time. Mean of 3 days for four parameters (ON with troublesome dyskinesia, ON with non-troublesome dyskinesia, ON without dyskinesia, OFF)	
Boston naming task	MADRS dichotomous >20 at 36 months	RUSH dyskinesia: Off dyskinesias and total dyskinesia score	
		30 s tap test: separately for each side, mean of two rounds	
		Parkinson’s KinetiGraph (PKG)—wrist-worn accelerometer readings (Lund participants only)	

AIMS, Abnormal Involuntary Movement Scale; EQ-5D-5L, EQ-5D-5L - Euroqol questionnaire; MADRS, Montgomery-Åsberg Depression Rating Scale; NMSS, Non-Motor Symptoms Scale; PDQ-39, Parkinson's disease Questionnaire 39; VAS, Visual Analogue Scale.

### Primary outcome

The primary objective of this first-in-human trial is to assess the safety, tolerability and feasibility of intraputamenal transplantation of the STEM-PD product in patients with moderate PD.

This will be measured by the number and nature of AEs and SAEs in the first 12 months following transplantation, as well as cranial MRI in the first 12 months following transplantation, looking for any space-occupying masses at the graft site.

### Secondary outcomes

The secondary objectives of the trial will be:

1. To evaluate the course and efficacy of the clinical features following intraputamenal transplantation of the STEM-PD product in patients with moderate PD.
2. To assess the survival of DA cells following transplantation of the STEM-PD product in patients with moderate PD using PET imaging.
3. To determine the safety and clinical efficacy between doses (if dose escalation is undertaken) of the STEM-PD product, including an assessment as to whether there is a dose–response effect.

The following secondary outcome measures will therefore be collected:

1. Changes in clinical measures at 36 months following transplantation compared with baseline (including emergence of new neurological features, including GIDs, global cognitive changes and changes in non-motor/quality of life assessments).
2. Changes in motor features in the OFF state at 36 months.
3. Change in anti-Parkinson medication as measured by L-dopa equivalent dose at 36 months.
4. [^18^F]F-DOPA uptake and DA transporter (DAT) binding (using [^18^F]FE-PE2i imaging) at 36 months on PET imaging with [^18^F]F-DOPA and [^18^F]FE-PE2i compared with PET imaging performed pre-transplant.
5. The number and nature of SAEs and AEs in the 12–36 months period following transplantation.

The secondary outcome measures will be reported with descriptive statistics for measurements at baseline and at 36 months. Absolute and relative differences will also be presented. MDS- UPDRS part III in ON and OFF state, AIMS sum score and PDQ-39 will also be described using line charts for all available time points.

[^18^F]F-DOPA PET imaging will be reported as absolute measurements of DA storage capacity measured by [^18^F]F-DOPA influx rate (K_i_), and relative measurements as measured by standardised uptake value ratios (SUVR) with occipital and/or cerebellar reference regions. K_i_ and SUVR values will be reported per brain hemisphere and per region of interest (ROI) in basal ganglia subregions, including the nucleus accumbens, pre-commissural ventral putamen, post-commissural ventral putamen, pre-commissural dorsal putamen and post-commissural dorsal putamen.

[^18^F]FE-PE2i-PET imaging will be reported as absolute measurements of DAT binding as ascertained using *BP*_ND_, and relative measurements as calculated using SUVR with occipital and/or cerebellar reference regions. The *BP*_ND_ and SUVR values will be assessed in the same ROIs as described for [^18^F]F-DOPA .

### Exploratory outcomes

The trial will collect the following data for exploratory outcomes:

1. Changes in F-DOPA uptake and DAT binding at 6 months post-transplantation on PET imaging with F-DOPA and PE2i compared with PET imaging performed pre-transplant.
2. Changes in F-DOPA uptake and DAT binding at 12 months post-transplantation on PET imaging with F-DOPA and PE2i compared with PET imaging performed pre-transplant.
3. Changes in F-DOPA uptake and DAT binding at 24 months post-transplantation on PET imaging with F-DOPA and PE2i compared with PET imaging performed pre-transplant.
4. Change in Fluctuation Dyskinesia Score compared with baseline as determined by measurement using wearable movement monitoring devices (Parkinson KinetiGraph).
5. Change in dyskinesia score compared with baseline as determined by measurement using wearable movement monitoring devices.
6. Change in Bradykinesia score features compared with baseline as determined by measurement using wearable movement monitoring devices.
7. Levels of donor cell-specific anti-human leucocyte antigen class I antibodies post-transplant.
8. Changes in markers of inflammation in the cerebrospinal fluid.
9. Change in response to an L-dopa challenge test, including measuring the duration and profile of the L-dopa effects between baseline and 36 months: change in MDS-UPDRS Part III score.
10. Change in response to an L-dopa challenge test, including measuring the duration and profile of the L-dopa effects between baseline and 36 months; change in AIMS score.

### Statistical considerations

No formal sample size calculations are planned, as this is a safety and tolerability trial, and the primary outcomes are descriptive. In addition, the trial is not powered for CIs of a given width. Instead, four participants will be dosed with dose 1, followed by four participants with dose 2 (or dose 1), as this was judged to be an appropriate number to give reliable information about the above outcomes, while not exposing a larger cohort to unknown risks. This number was derived in part from similar first in human studies done with transplants of human foetal DA cells and novel dopamine gene therapies, such as OXB-102 and ProSavin.^19 20^ This cohort size is similar to the number of patients that have been grafted in a recently published Japanese trial using iPSC-derived DA cells and was agreed on after detailed discussions with the regulatory agencies in Sweden and the UK (MPA and MHRA).^16^ Furthermore, trials of this size have been shown previously to identify specific side effects, including GIDs.^17 21^

The descriptive statistics will be computed using the Full Analysis Set (FAS). FAS is defined as all patients who received any study treatment. The number of missing values will be presented for each variable at each time point. The results in tables will be presented separately for each time point. Continuous and ordinal variables will be presented using number of non-missing observations, number of missing observations, median, minimum and maximum. The frequency (N) and percentages (%) (based on the non-missing sample size) of observed levels will be reported for all categorical variables.

## Ethics and dissemination

The STEM-PD trial was approved by the Swedish Ethical Review Authority (EPM Dnr 2021-06945-01) and the South Central—Oxford A Research Ethics Committee (reference 23/SC/0243). Clinical Trial Authorisation was given by the Swedish Medical Products Agency (Dnr: 5.1-2022-57953) and the Medicine and Healthcare products Regulatory Agency (reference CTA 40773/0001/001-0001). Authorisation for transfer to Clinical Trial Regulation (EU) 536/2014 was given by the Swedish Medical Products Agency (Dnr: 5.1.1-2024-100773).

Potential participants will receive verbal and written information about the trial and these will follow Good Clinical Practice, local regulatory and legal requirements, and the General Data Protection Regulation. The investigator or designee will ensure that each trial participant is fully informed about the nature and objectives of the trial and possible risks associated with their participation. All patients remain on the standard of care for the treatment of PD, under the care of their treating neurologist during the course of the trial. All patient information and consent documentation have been reviewed by the ethical review bodies in both countries and are provided in online supplemental file 1.

The results of the trial will be published in scientific journals and presented at international conferences. The results of the trial will also be disseminated directly to participants in the trial via a webinar or in-person event, as well as to the wider cohort of people with PD as part of an established programme of patient-public involvement in Cambridge (Annual Parkinson’s disease Open Day, NIHR Biomedical Campus Neuroscience theme day) and in Lund (Multipark Patient Café).

The STEM-PD trial opened and began recruitment in 2022. The first patient underwent transplant surgery in February 2023 and the final patient was recruited in August 2024 and received a graft in October 2024.

## Supplementary material

10.1136/bmjopen-2025-107597 online supplemental file 1
