# Supplementary material for: STEM-PD trial protocol: a multi-centre, single-arm, first-in-human, dose-escalation trial, investigating the safety and tolerability of intraputamenal transplantation of human embryonic stem cell-derived dopaminergic cells for Parkinson’s disease
Source: BMJ Open. 2025 Dec 30;15(12):e107597. doi: 10.1136/bmjopen-2025-107597 (PMC12766818; doi:10.1136/bmjopen-2025-107597)
Supplement: online supplemental file 1 [file bmjopen-15-12-s001.docx]

**PARTICIPANT INFORMATION SHEET & INFORMED CONSENT FORM**

**STEM-PD Trial:**

**A clinical trial to evaluate the safety of transplantation of stem cell-derived dopamine cells, that have not been tested in humans before, into the brain of individuals with Parkinson’s disease**

**(Formally registered as: A multicentre, single arm, first in human, dose-escalation trial, investigating the safety and tolerability of intraputamenal transplantation of human embryonic stem cell derived dopaminergic cells for Parkinson’s disease (STEM-PD product))**

You are being invited to take part in a research trial. Before deciding whether to take part, you need to understand why this research is being done and what it involves. Please take time to read the following information carefully and talk to others about the trial if you wish. Please ask us if anything is not clear or if you would like more information. Please take time to decide whether, or not, you wish to take part.

Section 1 tells you the purpose of this trial and what will happen to you if you take part.

Section 2 gives you more detailed information about the conduct of the trial.

A separate glossary has been written to accompany this information sheet where you can find definitions of the words that are emphasised in **bold** throughout this document.

**Table of Contents**

[**Table of Contents** 1](#_Toc166677477)

[Section 1: Purpose of the trial and what will happen 5](#_Toc166677478)

[1. What is the purpose of the trial? 5](#_Toc166677479)

[2. What is the treatment being tested? 5](#_Toc166677480)

[3. How will the STEM-PD cells be transplanted? 6](#_Toc166677481)

[4. Why have I been invited? 6](#_Toc166677482)

[5. Do I have to take part? 6](#_Toc166677483)

[6. What other medications will I need to take as part of this trial? 7](#_Toc166677484)

[7. What will I have to do? 9](#_Toc166677485)

[7.1 Trial visit flow chart 12](#_Toc166677486)

[8. What will happen to me if I take part? 14](#_Toc166677487)

[8.1 Screening visit (5 hours long) – part 1 15](#_Toc166677488)

[8.2 Screening visit (2.5 hours long) – part 2 – at Skåne University Hospital, Lund. 16](#_Toc166677489)

[8.3 Baseline visit (6.5 hours long) 17](#_Toc166677490)

[8.4 Travel to Lund for surgery 19](#_Toc166677491)

[8.5 One day before, and the day of surgery 20](#_Toc166677492)

[8.6 Follow-up procedures at day 1 and day 2 after the surgery 21](#_Toc166677493)

[8.7 Follow-up visits at 7, 14, 21, 28 and 42 days after the surgery (1 hour long) 22](#_Toc166677494)

[8.8 Follow-up visits at 2, 3, 4 and 5 months after the surgery (1 – 2.5 hours long) 23](#_Toc166677495)

[8.9 Follow-up visits 6 months after the surgery 23](#_Toc166677496)

[8.9.1 Part 1 - John van Geest Centre for Brain Repair (6 hours long) 24](#_Toc166677497)

[8.9.2 **OPTIONAL:** Part 2 - Skåne University Hospital, Lund (3.5 hours long) 25](#_Toc166677498)

[8.10 Follow up 7 and 8 months after the surgery 25](#_Toc166677499)

[8.11 Follow-up visit 9 months after the surgery (2.5 hours long) 25](#_Toc166677500)

[8.12 Follow up 10 and 11 months after the surgery 26](#_Toc166677501)

[8.13 Follow-up visits 12 months after the surgery 26](#_Toc166677502)

[8.13.1 Part 1 – John van Geest Centre for Brain Repair (6 hours long) 26](#_Toc166677503)

[8.13.2 Part 2 – Skåne University Hospital, Lund (up to 6 hours long, over up to 2 days) 26](#_Toc166677504)

[8.14 Follow up 13, 14, 15, 16, and 17 months after the surgery 27](#_Toc166677505)

[8.15 Follow up visit after completion of immunosuppressants (1 hour long) 27](#_Toc166677506)

[8.16 Follow-up visit 18 months after the surgery (2.5 hours long) 28](#_Toc166677507)

[8.17 Follow-up visits 24 months after the surgery 28](#_Toc166677508)

[8.17.1 Part 1 - John van Geest Centre for Brain Repair (5.5 hours long) 28](#_Toc166677509)

[8.17.2 **OPTIONAL:** Part 2 – Skåne University Hospital, Lund (up to 6 hours long, over up to 2 days) 29](#_Toc166677510)

[8.18 Follow-up visit 30 months after the surgery (1.5 hours long) 29](#_Toc166677511)

[8.19 Follow-up visits 36 months after the surgery 30](#_Toc166677512)

[8.19.1 Part 1 - John van Geest Centre for Brain Repair (6 hours long) 30](#_Toc166677513)

[Part 2 – 30](#_Toc166677514)

[8.19.2 Region Skåne – Skåne University Hospital (6 hours long, over 2 days) 30](#_Toc166677516)

[8.20 Enrolment in long term follow up study 31](#_Toc166677517)

[8.21 Schedule of trial procedures 32](#_Toc166677518)

[9. What are the side effects of the treatment being tested? 34](#_Toc166677519)

[10. What are the possible disadvantages and risks of taking part? 34](#_Toc166677520)

[10.1 Possible risks relating to the surgery 35](#_Toc166677521)

[10.1.1 Possible anaesthesia risks 35](#_Toc166677522)

[10.1.2 Possible risks of the **NG tube** 35](#_Toc166677523)

[10.1.3 Possible risks associated with transplantation 35](#_Toc166677524)

[10.1.4 Possible other risks 36](#_Toc166677525)

[10.2 Possible risks relating to other trial procedures 36](#_Toc166677526)

[10.2.1 Immunosuppressive treatment 36](#_Toc166677527)

[10.2.2 Antibiotic treatment 37](#_Toc166677528)

[10.2.3 Temporarily stopping your PD medication for some assessments (**OFF** **medication** state) 38](#_Toc166677529)

[10.2.4 Blood samples 38](#_Toc166677530)

[10.2.5 **Optional** lumbar puncture 38](#_Toc166677531)

[10.2.6 **MRI** scans 39](#_Toc166677532)

[10.2.7 **PET** scans 40](#_Toc166677533)

[10.2.8 CT angiography 40](#_Toc166677534)

[10.2.9 Assessments/questionnaires 40](#_Toc166677535)

[10.2.10 Incidental findings 41](#_Toc166677536)

[11. What are the possible benefits of taking part? 41](#_Toc166677537)

[12. What are the alternatives for treatment? 41](#_Toc166677538)

[13. What happens when the trial stops? 41](#_Toc166677539)

[14. Expenses & payment? 41](#_Toc166677540)

[Section 2: Trial Conduct 42](#_Toc166677541)

[15. Who is the Sponsor for the trial? 42](#_Toc166677542)

[16. What if new information becomes available? 42](#_Toc166677543)

[17. What if I decide I no longer wish to participate in the trial? 42](#_Toc166677544)

[18. What if there is a problem? 43](#_Toc166677545)

[19. How will we use information about you? 44](#_Toc166677546)

[**19.1** **What are your choices about how your information is used if you change or stop your participation in the trial?** 46](#_Toc166677547)

[19.2 Where can you find out more about how your information is used? 46](#_Toc166677548)

[20. What will happen to my samples? 46](#_Toc166677549)

[21. How will results of the trial be published? 47](#_Toc166677550)

[22. Who is funding the trial? 48](#_Toc166677551)

[23. Who has reviewed this trial? 48](#_Toc166677552)

[24. Further information and contact details 48](#_Toc166677553)

[*In the event of an emergency please contact*: 49](#_Toc166677554)

[INFORMED CONSENT FORM 49](#_Toc166677555)

# Section 1: Purpose of the trial and what will happen

## What is the purpose of the trial?

One of the most promising treatments to repair the damage caused in Parkinson’s disease (PD) to date has been the transplantation of **dopamine** cells obtained from human **foetal tissue** into the brain. However, this has given inconsistent results, with some patients doing extremely well and coming off all their anti-PD medication for years, whilst others have shown no or only modest clinical improvements. In some cases, patients also developed new involuntary movements called **graft-induced dyskinesias (GIDs)**. Due to practical and ethical issues with using **foetal tissue**, work began around 10 years ago to make **dopamine** **neurons** from human **stem cell** sources, rather than relying on **foetal tissue**. **Stem cells** are able to develop into many different cell types, including brain cells and in this trial, we have made them into **dopamine** cells specifically for testing in PD.

In this trial, we are assessing a **stem cell** therapy, called **STEM-PD**, in people with PD. We are aiming to assess whether the **STEM-PD** cells are safe to use in people with PD, as well as looking at which dose of the **STEM-PD** cells should be optimally used in future trials. We will be looking for signs that the **STEM-PD** cells mature to **dopamine** **neurons** and produce **dopamine** in the brain after being transplanted. The trial will investigate potential signs of benefit to people with PD by looking at features of the disease and seeing if they improve.

The **STEM-PD** cells have not been used in humans before.

## What is the treatment being tested?

**STEM-PD** is a **stem cell** therapy made of **dopaminergic progenitor cells**, which give rise to the **dopamine**-producing **neurons** found normally in the human brain and which are specifically lost in PD. The **STEM-PD** cells come from a single source. In this trial, we plan to transplant the **STEM-PD** cells into both sides of the brain in one surgical procedure. In the event of an interruption to the transplant, for example where it is medically required, we will discuss options with you for completion of the transplant. When transplanted, we hope that these **dopaminergic progenitor cells** will mature into **dopamine**-producing **neurons**, increasing the level of **dopamine** in the brain.

This is the first time the **STEM-PD** cells will be given to humans. **STEM-PD** is not licenced for use in any country.

The **STEM-PD** cells have been tested in animal studies and have been shown to be safe and show signs of efficacy.

We plan to test two different doses of **STEM-PD** in this trial to see which will be most suitable for future studies. The starting dose is a dose of cells that we believe is likely to be therapeutic and will be transplanted to the first 4 participants. To ensure that we are not using a dose that is too low, we also plan to test a higher dose in a further 4 patients. An independent committee made up of experts in PD and cell transplantation to the brain will review the data from the first dose, and help us to decide if, and when, we progress to the higher dose. Your trial doctor will discuss with you the dose that you will receive.

## How will the STEM-PD cells be transplanted?

The **STEM-PD** cells will be transplanted into the brain using the **Rehncrona-Legradi device** (**R-L**). The **R-L** is a neurosurgical cell transplantation device that is manufactured by Region Skåne, a University Hospital, and approved for use in this trial by the Medical Technology department at Region Skåne in Lund, Sweden. The device has been used for over 30 years in numerous operations and in several clinical studies of cell transplantation to the human brain, including the **foetal tissue** transplants completed as part of the TransEUro study.

The **R-L** is a non-CE marked device, meaning it is not a licenced instrument and it cannot be used outside Region Skåne – Skåne University Hospital. Therefore, the transplant surgery will take place at Region Skåne – Skåne University Hospital, Lund, Sweden.

The use of the **R-L** is not under investigation in this trial.

## Why have I been invited?

You have been invited to participate in this trial because you have PD and have previously shown a good response to oral **dopamine** therapies (meaning the **STEM-PD** cells may be a suitable treatment for you). You have also been approached as you have previously taken part in the ongoing TransEUro observational study.

We plan to include 8 participants with PD from the UK and Sweden.

## Do I have to take part?

Taking part in this trial is completely voluntary. If you decide to take part you will be asked to sign an informed consent form; however, you are still free to change, limit your commitment, or completely withdraw after you agree to join this trial.

The only exception to this is where your participation must change or be stopped by your trial doctor in order to protect your safety or well-being.

You can leave/change your participation in the trial at any time without giving a reason. If you choose not to participate or to leave the trial, your future medical treatment and normal standard of care will not be affected in any way.

Your participation in this trial will last for 3 years, thereafter the expectation is that you agree to participate in a separate long-term follow-up trial. This is so that we can continue to follow your progress over an extended period of time due to this being a ‘first in human’ trial.

## What other medications will I need to take as part of this trial?

You will be required to take other medications as part of your involvement in this trial. These fully licenced medications are not being tested as part of the trial and are safe to use within the remit of this trial. You will be given a medication schedule to remind you when to take these other medications.

It is very important that you take these medications as instructed by the trial doctor. We will check that you are taking these medications as directed and ask you about any side effects at your trial visits.

*Immunosuppressants*

The day before your surgery, you will be started on **immunosuppressants**, which are medications that reduce the strength of the body's immune system. **Immunosuppressants** are commonly used in transplantation surgery to prevent the body from rejecting a transplanted organ/cells.

In this trial, the **immunosuppressants** aim to protect the transplanted cells from being rejected by your immune system. You will be given a combination of **immunosuppressant** medications, based on what is used as standard following organ transplantation, and you will continue these medications for up to 18 months following your surgery. As these medications suppress your immune system, we will closely monitor you for any side effects, as well as reviewing other medications you are taking for any potential interactions.

The **immunosuppressants** used in this trial will be a combination of the following medications:

- Prednisolone (a type of medication known as a corticosteroid)
- Tacrolimus (or cyclosporine if you are not able to take tacrolimus, for example if you cannot tolerate it)
- Azathioprine
- Basiliximab

When taking **immunosuppressants** you should avoid grapefruit, grapefruit juice, concentrated citrus seed capsules, cannabidiol, and other CBD preparations as they can affect how the **immunosuppressants** work.

The trial doctor may decide that you will also need to take medications to protect your stomach whilst you are taking prednisolone. This will be discussed with you if it is required.

If you want to know more about the **immunosuppressant** medications above, please ask your trial doctor who will be able to provide you with further information.

*Medications to reduce the risk of osteoporosis*

As described above, prednisolone is a corticosteroid. The long-term use of corticosteroids increases the risk of a condition called **osteoporosis**, a disease that weakens bones, making them fragile and more likely to break. To reduce the risk of you developing this condition, you will receive two supplements, as detailed below.

Alendronic acid

When you are started on **immunosuppressants**, you will also be given a 70 mg tablet of alendronic acid (or an equivalent locally approved therapy) to take once a week. Like all medicines, alendronic acid can causes side effects in some people. However, many people do not experience side effects or only minor ones whilst taking this supplement. You will need to stay on alendronic acid whilst you are taking prednisolone.

Calcium/vitamin D supplements

You will also be given a supplement containing calcium and vitamin D. Like all medicines, side effects are possible, however, many people have no side effects or only minor ones with this supplement. As with alendronic acid, you will start taking this supplement at the time as the corticosteroids and remain on them until you stop taking the prednisolone.

*Antibiotics*

As is standard procedure for surgery, you will be given antibiotics at the time of surgery and for up to 48 hours post-surgery. The trial doctor will determine which antibiotics you are given before your surgery, based on the standard procedure at the hospital.

Additionally, whilst you are on **immunosuppressants**, you will also be given the antibiotics trimethoprim and sulfamethoxazole to reduce your risk of infection. These antibiotics are commonly used to treat people who are susceptible to infections. Whilst you are taking the **immunosuppressants** you will be given trimethoprim and sulfamethoxazole in a ratio of 1:5 three times a week. Common side effects of this medication include:

- Diarrhoea
- Electrolyte imbalance - in most cases, this refers to a minor imbalance in some of the elements measured in blood which typically do not cause any signs of symptoms, however if the degree of imbalance is greater, that can make people feel tired, weak and generally unwell.
- Fungal overgrowth – this typically occurs in the mouth and is often known as oral thrush and if present may give you a dry mouth, altered sense of taste and/or slight irritation. Occasionally this can spread to the digestive system where it can give the sensation of a filled stomach, heartburn or weight loss.
- Headache
- Nausea
- Rashes

***PET*** *scan tracers*

**PET,** or positron emission tomography, imaging scans will be used within the trial to examine and create a detailed image of the **dopamine**-producing **neurons** in your brain. **PET** is a technique that uses a radioactive material (called a ‘tracer’) which is injected into the bloodstream where it travels throughout the body and then into the brain. **PET** scans have been done for over 30 years. The radioactive tracer emits a signal which is detected by the scanner, leading to the generation of a 3D colour image on a computer. You will have a total of 7 **PET** scans over the duration of the trial.

In this trial, two tracers are being used, called ^18^F-fluorodopa (^18^F-FDOPA) and ^18^F-FE-PE2i. These tracers will give us different pieces of information, but together they will allow us to see if the transplanted cells survive and mature into **dopamine neurons** in the brain. For the **PET** scans you will have an injection through a cannula into a blood vessel in your forearm of a short-lasting radioactive liquid. The injected radioactivity fades away naturally over a few hours. Only one tracer will be injected per scan.

For the ^18^F-FDOPA scans, you will be given 150 mg of a medication called carbidopa, about 1 hour before the injection of the radioactive tracer. The carbidopa will help the 18F-FDOPA tracer travel to the brain, by stopping it being broken down by certain enzymes in the rest of the body.

## What will I have to do?

We know that taking part in a trial can be quite daunting, so if you decide to take part, we will guide you through what is required from you:

- You will be asked to attend all the trial visits and provide regular updates on your participation as detailed in section 8 and depicted in the trial visit flow chart at this end of this section.
- You will be given medication in addition to your normal anti-PD medications to take every day. It is important that you take all the provided medication as directed by your trial doctor.
- During the trial you should continue taking your PD treatment in the same way as before. If your neurologist feels that your PD treatment should be stopped or changed, then you are free to do so. Please inform the trial team of any changes to your PD treatment.
- There are some medications and supplements which may interact with the medications you are given in the trial. It is therefore important that you consult with the trial team before starting any new medication or supplements, including those bought over the counter.
- You should tell the trial team if you feel unwell or different in any way. If you have any major concerns or are feeling very unwell, please contact your trial doctor immediately using the contact numbers at the end of this information sheet.
- If you do participate, we will inform your GP that you are taking part.
- You should also discuss your participation in this trial with any insurance provider you have (e.g., life insurance, income protection, critical illness cover, and private medical insurance) and seek advice if necessary, as failure to notify them may affect or invalidate your cover.
- To take part in this trial, you will need to have had a full COVID-19 vaccination dose before you receive trial treatment.
- Due to the required commitment and procedures of this trial we suggest you speak to family members or friends who could support you throughout your participation, should you decide to take part.
- You will need to have a valid passport for the duration of the trial (3 years).
- If you choose to take part in the trial, we will want to continue to monitor you for life. You will therefore be asked at the end of the trial if you are willing to consent to a separate long-term follow-up trial.

Please share this information with your partner if it is appropriate.

- Some of the medicines you are given as part of this trial could harm an unborn baby or nursing infant. You will not be able to take part in this trial if you are pregnant or breastfeeding. You should not participate in this trial if you are planning to become pregnant or father a child during the period of **immunosuppressant** treatment.
- Participants of childbearing potential must use effective contraception for the duration of the **immunosuppressant** treatment, plus an additional 3 months after completion of **immunosuppressant** treatment. For the purpose of the trial, a person is considered to be of childbearing potential following their first menstrual period until post-menopausal, unless surgically sterile. Permanent sterilisation methods include hysterectomy, bilateral salpingectomy, and bilateral oophorectomy. A post-menopausal state is defined as no menstruation for 12 months without an alternative medical cause. For participants of childbearing potential, one form of highly effective contraception should be used from the following:
  - Combined (oestrogen and progestogen containing) hormonal contraception associated with inhibition of ovulation
    - Oral
    - Intravaginal
    - Transdermal
  - Progestogen-only hormonal contraception associated with inhibition of ovulation
    - Oral
    - Injectable
    - Implantable
  - Intrauterine device (IUD)
  - Intrauterine hormonal-releasing system
  - Bilateral tube occlusion
- Men must use an effective form of contraception for the entire duration of the **immunosuppressant** treatment. This includes:
  - Condom and spermicide (chemical that kills sperm) even if female partner(s) is/are using another method of contraception or are already pregnant.
- You do not need to use contraception if:
  - you are a woman and only have one male partner who has had an operation to cut the tubes that carry sperm (vasectomy)
  - you (or your partner) are a woman who cannot become pregnant
  - you are a man and you have had a vasectomy
  - you practice true abstinence as part of your usual and preferred lifestyle (no sexual activity from 6 weeks before your screening visit until you have completed **immunosuppressant** treatment if you are male or three months after completing **immunosuppressant** treatment if you are female). If you become sexually active, you must use one of the methods listed above.
- If you or your partner becomes pregnant during the trial, you should inform your trial doctor immediately. Your trial doctor will discuss all the options available to you and will liaise with your GP and other health professionals where appropriate.
- You also may not donate sperm for the duration of the time you are taking **immunosuppressants**.

###
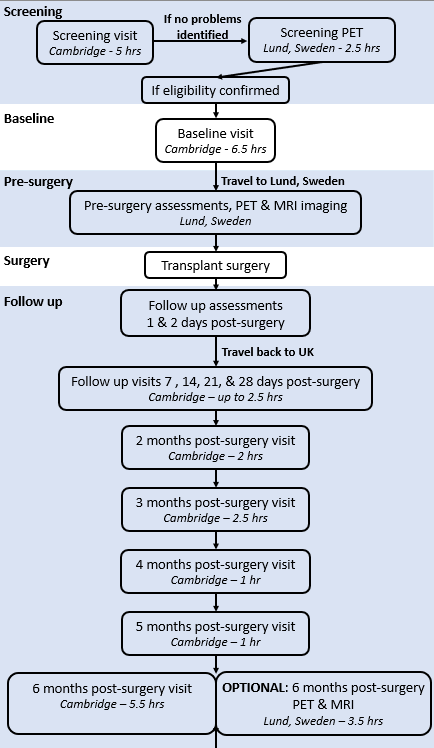
 7.1 Trial visit flow chart

Continued on the next page


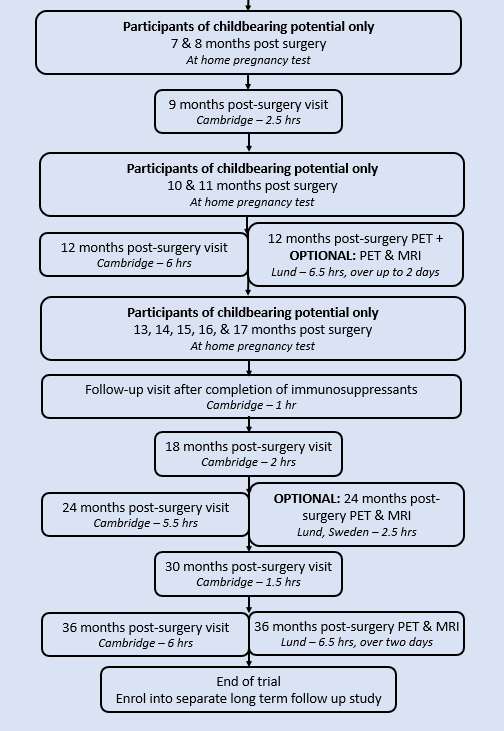


## What will happen to me if I take part?

If you agree to participate in the trial, you will be asked to sign the informed consent form at the end of this document and be given a copy to take away and refer to later.

The maximum trial duration is 40 months, including a screening period of up to 4 months, and a 36-month follow-up period after the transplant. The transplantation surgery will take place at Region Skåne – Skåne University Hospital in Lund, Sweden. You will need to be able to travel to Lund in Sweden to take part in this trial. There are up to 25 visits in total that you would need to attend as part of this trial. Some (up to 6 visits) of the trial visits will require travel to Region Skåne – Skåne University Hospital in Lund, Sweden for pre-operative assessments, surgery, and specialist imaging. Most of the visits will occur at the John van Geest Centre for Brain Repair (VGB), based on the Cambridge biomedical campus, with **MRI** imaging being performed at the Wolfson Brain Imaging Centre (which is also on the Cambridge biomedical campus). Some visits/assessments may be carried out at the Wellcome Clinical Research Facility which is also based on the Cambridge biomedical campus near to the VGB. The details and locations of these visits and assessments are indicated below.

Some visits are longer than others, but there will be an opportunity to take breaks between each examination or test. If required, you can complete the visit over two days. Please let the trial team know if you would like to spread the assessments over two days. Refreshments are available to trial participants, including teas, coffee, and cold drinks. If you have any special requirements, then please let the trial team know.

Attending visits OFF medication

For up tp 12 of the visits, we will ask you to attend **OFF** your usual anti-PD medication prior to the visit. You should continue any other medication for other conditions as normal. This means that you will need to stop taking your PD medication 12 hours before your visit or 24-hours before for long-acting **dopamine** agonists (e.g. ropinirole XL) and MAO-B inhibitors (e.g. rasagiline). Any other medications that interfere with the imaging used in the trial will need to be stopped for 24 hours before the scan. This will be discussed with you. Please contact the trial team if you are unsure. To help you remember when to stop taking your medication, your appointment letter will tell you exactly which doses you need to miss. As missing this medication can be difficult for some people, wherever possible your trial visit will be scheduled for the morning. If it is impossible for you to travel to the trial visit whilst **OFF** **medication**, we can arrange overnight accommodation for you local to the site.

We ask you to come in **OFF** **medication**, so we can assess your ability to do specific assessments in an **OFF** **medication** state. During these visits, after these specific assessments have been completed, we will ask you to take your normal anti-PD medication and repeat some of these assessments when you feel your medication is working, i.e., when you are in an **ON** **medication** state. Apart from these specific visits, you can otherwise continue taking all of your PD medications as normal. If your doctor thinks that your PD medication or dose needs changing, then that is absolutely fine. We will keep a record of this by asking you about this when you come back for your trial visits.

**Filming assessments**

Some of the assessments will be filmed to allow a second assessor to independently score your response. To ensure that it is not possible for the independent reviewer to distinguish before and after surgery you will need to wear a cap for the duration of these assessments. This will be provided to you during your visit.

**COVID-19 guidance**

During any COVID-19 or similar pandemic, strict safety measures will be in place during visits to the research centres to minimise your risk of infection. This may include members of staff wearing surgical face masks, aprons and gloves, and you may also be asked to wear a face covering, in line with the current guidance at the time of the visit. More information regarding any safety measures in place will be sent to you before your appointments.

Brain donation

If you decide to participate in this trial, we will ask you to become a brain donor for the Cambridge Brain Bank, based at Addenbrooke’s Hospital, Cambridge University Hospital. All information regarding this will be given to you in a separate participant information sheet and an expert in this area will be available to talk to you about your decision to donate. Becoming a brain donor is completely optional. If you decide not to become a brain donor, this will not affect your participation in this trial or your future care.

Details of the trial visits are described below. A flow chart of the visits is provided in section 7.1: Trial visit flow chart**.**

### Screening visit (5 hours long) – part 1

Prior to agreeing to take part in the trial, you will be given the opportunity to discuss the trial with your trial doctor in detail. You can ask as many questions as you like during this discussion. We will contact you at least two weeks after you have received this information leaflet. If you are still interested in taking part, a screening visit will be arranged for you where you will be asked to sign the informed consent form at the end of this document. You can contact your trial doctor, or a member of the trial team at any time throughout the trial should you have any questions. Contact details are included at the end of this document (section 24).

You will need to come to this visit **OFF medication**.

During your visit, we will:

- Ask you to perform **motor assessments;** assessments that measure the movement and actions of your body. These will be carried out first so you can take your usual PD treatment as soon as possible after completing these tasks, before continuing with the remainder of the assessments. These **motor assessments** will involve a physical examination by a trial doctor to assess your movement, including feeling for stiffness in your limbs, asking you to perform some repetitive movements, asking you to stand up and walk, checking your balance and examining for tremor and involuntary movements. You will be videoed for these assessments and will need to wear the provided cap. You will also be asked about symptoms you may experience with your PD. The same **motor assessments** will be repeated once you have taken your usual PD medication and have reached an **ON** state.
- Review and record your medical history and all the medications you are currently taking.
- Record your height and weight.
- Take a blood sample of 44 ml, about 3 tablespoons for routine blood tests. This is done to find out whether there are any reasons why it would not be suitable for you to take part.
- We will collect a swab for a bacteria called Methicillin-resistant Staphylococcus aureus (or **MRSA**).
- For participants who are able to become pregnant (of childbearing potential), an additional blood sample of 5ml, about 1 teaspoon, will be taken to allow a pregnancy test to be performed.
- Perform a physical examination, which will include the trial doctor checking your general physical health to ensure there are no problems with you taking part in the trial.
- Perform a screening **MRI** scan to check your eligibility for the trial. This scan can be noisy and claustrophobic. See section 10.2.6 for more details about the scan.
- Perform an assessment of your memory and thinking processes, using a standard set of questionnaires and tests.
- Assess your mental health, using a standard questionnaire. This may contain some questions that some people will find sensitive.

### Screening visit (2.5 hours long) – part 2 – at Skåne University Hospital, Lund.

Providing we don’t identify any reasons why you might not be appropriate for the trial from your first screening visit, you will be made an appointment to undergo a **PET** scan at Skåne University Hospital. This scan will be combined with a **CT** (or computerised tomography) scan. You may be accompanied by a member of the trial team from Cambridge, and you may bring a companion (i.e., family member or friend) with you. All travel, accommodation and living costs will be covered.

For your **PET** scan you will need to be **OFF** **medication**. The trial doctor will confirm the details with you. Before the scan, you should also avoid a heavy meal and eat only a low protein breakfast. Example meals will be discussed with you. You should not consume any caffeine (i.e., tea, coffee, and energy drinks) or smoke on the day of the scans. About an hour before the scan, you will be given 150 mg of a medication called carbidopa.

For the **PET** scan you will be asked to lie down with your upper body inside the scanner and have an injection through a cannula placed into a blood vessel in your forearm of about a third of a tablespoon of the short-lasting radioactive liquid. You will be given earplugs to protect your ears from the noise created by the scanner. The scan will last 90 minutes from when you have the injection and during this time you will be asked to keep your head as still as possible. The injected radioactivity fades away naturally over a few hours and you can leave the scanning centre 30 minutes after the scan ends. You will be in constant contact with the **PET** technician and/or MR operator (the person who operates the scanner) and the researcher who will make sure that you are comfortable. If at any point you need to stop or take a break, please tell the research staff. Throughout the visit, we will make every effort to answer any questions you may have or clarify anything you are unsure about.

With the results of all of the screening assessments, your trial doctor will run through a checklist to make sure you are suitable (‘eligible’) to take part in the trial. Once we know the results, we will contact you, by email or telephone, to confirm whether or not you are eligible to participate in the trial. In some circumstances, the results of the scans or the screening assessments from your first visit may preclude you from continuing in this trial. If this happens, it will be explained to you as to why this is the case, and you will continue to receive the same healthcare you would normally have received.

We will use the results of your screening assessments to check you are appropriate for the trial. If you are found to be suitable, then a baseline visit will be arranged, surgery will be discussed with you and scheduled.

### Baseline visit (6.5 hours long)

Before your visit you will be provided with a diary card (called the Hauser patient diary card). The Hauser patient diary card will be used to record information on your PD symptoms during a normal day. You will be asked to record your mobility every half hour for 3 consecutive days prior to the baseline visit, and the trial team will explain to you when this should be done. At your baseline visit, we will collect the completed diary cards.

Your baseline visit will take place up to 60 days before your surgery. As with the screening visit you will need to come for this visit **OFF medication**. Before this visit, you should avoid a heavy meal and eat only a low protein breakfast. Example meals will be discussed with you. At this visit we will:

- Measure your ability to perform certain **motor assessments**. These tests will be more extensive than the ones completed at your screening visit. These will be carried out first when you are **OFF medication** so you can take your usual PD treatment as soon as possible. You will be given a soluble form of levodopa and asked to repeat some assessments. This is so we can assess your movement in both the **OFF** and **ON** state. After these assessments you can take your normal medications. Once you feel they are working at their best, you’ll be asked to repeat some further tasks. Some of the assessments will also be filmed. As at your previous visit, you will need to wear the provided cap for the duration of the assessments being filmed.
- For participants of childbearing potential, a urine pregnancy test will be performed.
- Assess your memory and thinking processes using a standardised set of questionnaires and tests. Some of these tests will be done before you take your PD medications.
- Review your medical history and all the medications you are taking and ask you about your symptoms.
- Record your vital signs (blood pressure, pulse, temperature), and weight.
- Perform an **electrocardiogram** (or **ECG**) which looks at your heart rate, rhythm and conduction.
- A repeat physical examination will be performed.
- Perform questionnaires relating to other non-motor symptoms of PD and quality of life.
- Assess your mental health, using a standardised set of questionnaires and tests. As previously outlined, these may contain some questions that some people will find sensitive.
- You will be provided with a participant alert card. You should keep this card with you at all times and show it to anyone who gives you medical attention.
- An **optional** blood sample of 5 ml, less than a tablespoon, may be collected, for further research. It is entirely up to you whether you decide to have this done or not. The sample that we collect may be used to look at your DNA and blood serum elements and will be retained for future research.
- Perform an **optional** lumbar puncture to collect a small sample (10 ml, about 2/3 of a tablespoon), of **cerebrospinal fluid** (**CSF**). An additional blood sample of around 11 ml, or just under a tablespoon, will also be collected. It is entirely up to you whether you decide to have these procedures. More details about the lumbar puncture are included below. The samples that we collect will be used to measure markers of inflammation and disease state, and to investigate how these change throughout the trial.

Lumbar puncture

The lumbar puncture is completely **optional**. During the trial, we plan to perform five lumbar puncture procedures, if you consent to these. You are free to decline further lumbar punctures at any time in the trial without giving a reason and without it affecting your future care or medical treatment or participation in the trial.

A lumbar puncture (or ‘spinal tap’) is a standard procedure used to obtain a sample of **CSF**. This fluid cushions your brain and spinal cord and contains important proteins and salts similar to blood. A lumbar puncture involves you lying down comfortably on one side, and a trained health professional taking a sample of **CSF** though a small needle which is placed in the lower back, below the level of the spinal cord. The procedure can be uncomfortable so to minimise this, the skin of the lower back is numbed with local anaesthetic before a very fine needle is introduced to obtain the sample. The procedure takes 10-15 minutes. You may wish to rest for up to an hour afterwards, but you will be free to go home as soon as you feel ready to do so. You do not need to take any special precautions afterwards.

### Travel to Lund for surgery

Approximately 7 days before your planned surgery date, you will fly to Lund in Sweden. All transport, including flights, and transfers to and from the airports will be arranged for you. You can bring a companion with you if you wish. Accommodation for yourself and your companion will be arranged for you at or near to the Skåne University Hospital campus for the duration of your stay in Lund. All travel, accommodation and living costs will be covered.

You should bring your own PD medication to Lund, ensuring you bring enough medication for the duration of your stay.

A member of the Cambridge STEM-PD trial team will also travel to Lund prior to your surgery. You will not share accommodation with any of the Cambridge team.

When you are in Lund, you will:

- Have pre-surgical checks as is the standard procedure at Skåne University Hospital. This may include a blood sample of 29 ml, about 2 tablespoons, done to perform the blood tests routinely completed before any surgery.
- Have a surgical planning **MRI** scan. This **MRI** scan will be completed under general anaesthesia to generate high resolution images for neurosurgical planning purposes. This scan is likely to be short (< 30 minutes) but may lead to discomfort in the throat and mouth.
- Have a **CT** scan angiogram. **CT** scans use X-rays and a computer to create detailed images of the inside of the body. This scan will be used to give better visualisation of the blood vessels in your brain.
- Have a **PET** scan. This scan will be combined with a **CT** scan. You will need to be **OFF** **medication** for the **PET** scan. Before the scan you should avoid a heavy meal and eat only a low protein breakfast. You should not consume any caffeine (i.e., tea, coffee, and energy drinks) or smoke on the day of the scan.
- On the same day as the **PET** scan, you will also have a separate **MRI** scan whilst you are still **OFF medication**. If for any reason it is not possible to complete the **MRI** scan on the same day as the **PET** scan, it may be performed the following day. Should this happen the trial doctor will discuss with you whether you need to be **OFF** or **ON** your medication for the scan.

### One day before, and the day of surgery

You will attend Region Skåne – Skåne University Hospital the day before the operation and you will be admitted overnight. A member of the Cambridge team will accompany you for this visit. Prior to your surgery, in line with the standard procedures, you will be told which medications, if any, you will need to stop taking. It is at this point that you will be started on **immunosuppressants** and antibiotics. You will be required to stay in hospital for at least 4 days after surgery. If it is felt necessary by your trial doctor, then you may need to stay for longer.

Final pre-operative checks will take place, which will involve:

- Asking how you have felt since your last visit along with a review of all your medications.
- A blood sample of 29 ml, about 2 tablespoons, will be taken if this has not already been completed in the 3 days prior to your surgery. This is done to perform the blood tests routinely completed before any surgery.
- For participants of childbearing potential, a pregnancy test will be performed.
- Recording your vital signs and weight.

As you will need to have a general anaesthetic, you will not be allowed to eat for around 6 hours before the operation. You will be allowed clear liquids (e.g. water) until 2 hours prior to the surgery.

On the day of surgery, your head will be washed with special soap and either minimally or fully shaved in preparation for surgery. After this, a frame will be fitted to your head, which will hold your head in place during the surgery. The frame will be fitted under local anaesthesia if you agree to this, but general anaesthesia can be used at this point if you would prefer. This will be discussed with you. This frame is a standard piece of equipment used in neurosurgery. Once the frame is in place, you will have an **MRI** scan to allow the surgeons to plan the precise area of your brain into which the **STEM-PD** cells will be transplanted. Following these procedures, you will be anaesthetised for surgery, and a catheter and a **nasogastric** (**NG**) **tube** will be inserted according to standard procedures. A **NG tube** is a flexible tube of rubber or plastic that is passed through the nose, and down through the throat into the stomach to provide support to your stomach during the surgery. The **NG** **tube** might also be used to deliver medication before and after surgery.

*Surgery*

A neurosurgeon will make one or more small holes (known as **burr holes**) on each side of your skull to allow access to your brain. Using the **R-L** device, transplants of the **STEM-PD** cells will be made along 5 paths on each side of the brain. In some circumstances it may be necessary for the sponsor investigator to be present during the transplantation. You will be informed ahead of your surgery if they will be present. All of the transplants will be done in one operation, which is expected to take up to 12.5 hours (including the **MRI** scanning and wake-up time after the surgery). The operation will end by closing the skin over the **burr holes** with sutures or standard skin staples, which will be done following standard processes. To minimise the risk of infection, the **burr holes** will not be filled in at this stage. This leaves a slight indentation in the skull which is covered by your skin. You can ask your trial doctor to see an image of what these **burr holes** look like. It is routine for all neurosurgical procedures on the skull to leave these unfilled as there is no risk from doing this. Following the completion of your **immunosuppressants** you can request for the **burr holes** to be filled, which would involve an additional surgery under local anaesthesia in the UK.

### Follow-up procedures at day 1 and day 2 after the surgery

After surgery, you will be taken to the recovery room and then back to the ward. You will also be commenced on the medications to prevent **osteoporosis**; alendronic acid and calcium/vitamin D supplements (or an equivalent approved therapy). For the first three days there will be limits on your ability to leave your room and for your companion to visit you. This is for your protection as you will be in the most intense period of **immunosuppressant** treatment and therefore most at risk of catching an infection. Normal medications will be restarted as soon as possible after the surgery, in line with standard prescribing procedures. Pain medication can also be given post-surgery in line with standard processes. You will be looked after by the hospital staff following the usual hospital procedures after neurosurgery. You will be accommodated by an English-speaking person although many of the medical team in Lund speak fluent English. In addition to the standard procedures after surgery:

- Your vital signs will be recorded.
- A blood sample of 13 ml, or a tablespoon, will be taken at day 1 and day 2 after the surgery to perform routine and safety blood tests.
- At day 2 only, you will have an **ECG.**
- At 4 days after the surgery, you will receive a second dose of the **immunosuppressant** basiliximab.
- In addition, you will have an **MRI** scan of your brain within 3 days of your surgery to check for any complication and the location of the transplanted cells.

At 5 days post-surgery you will travel back to the UK as long as the doctors are satisfied with your recovery after surgery. Members of the Cambridge STEM-PD trial team who travelled to Lund prior to your surgery may travel back with you. If there is any medical reason you cannot travel back at 5 days post-surgery, you will remain in Lund until you are fit to travel. Any additional accommodation and living costs will be covered. You will be reminded of your follow-up appointments and be given a weeks’ supply of the trial medications.

One week after your surgery you will be allowed to drive a car, providing there are no complications from the transplant surgery and new medications.

### Follow-up visits at 7, 14, 21, 28 and 42 days after the surgery (1 hour long)

Before every appointment you will receive an appointment letter to remind you whether you need to come to your visit **ON** or **OFF** your medication.

You will need to attend the John van Geest Centre for Brain Repair on days 7, 14, 21, 28 and 42 after your surgery. You will need to come to these visits **ON** **medication.** A mutually convenient date for these appointments will be made for you by the research team. At each visit we will:

- Ask how you have felt since your last visit, and review all of your medications.
- Record your vital signs and weight.
- Perform a physical examination.
- Collect a blood sample of 16 ml, just over a tablespoon, for routine and safety blood tests. One of the blood tests will check the levels of the **immunosuppressant** tacrolimus (or cyclosporine, depending on which medication you are taking) in your body. You will therefore need to miss your morning dose of tacrolimus (or cyclosporine) on the day of the visit.

At the **28 days** post-surgery visit the following additional activities will take place:

- A repeat **MRI** scan, which will take place at the Wolfson Brain Imaging Centre.
- For participants of childbearing potential only, a urine pregnancy test will be performed.
- Assess your mental health, using a standardised set of questionnaires and tests. As previously outlined, these may contain some questions that some people will find sensitive.

These additional assessments will take an additional 1.5 hours to complete, meaning the 28 day post-op visit will take approximately 2.5 hours.

### Follow-up visits at 2, 3, 4 and 5 months after the surgery (1 – 2.5 hours long)

At 2, 3, 4 and 5 months after the surgery you will be made an appointment, at a mutually convenient date and time, to attend the John van Geest Centre for Brain Repair. You will need to come to these visits **ON** **medication**. At these visits, we will:

- Ask how you have felt since your last visit and review all your medications.
- Record your vital signs and weight.
- A repeat physical examination will be performed.
- Collect a blood sample of up to 28 ml, around 2 tablespoons, for routine and safety blood tests. One of the blood tests will check the levels of the **immunosuppressant** tacrolimus (or cyclosporine) in your body. You will therefore need to miss your morning dose of tacrolimus (or cyclosporine) on the day of the visit.
- For participants of childbearing potential only, a urine pregnancy test will be performed.

At the **2 and 3 month visits only,** you will additionally undergo a repeat **MRI** scan at the Wolfson Brain Imaging Centre. The **MRI** scan will take approximately 1 hour.

At the **3 month visit only**, we will also:

- Measure your ability to perform certain **motor assessments**. Some of these assessments will also be filmed. As at your previous visits, you will need to wear the provided cap for the duration of the assessments being filmed.
- Collect your Hauser patient diary card . You’ll be posted the diary ahead of your visit with instructions on what you need to do. As for the baseline visit, you’ll need to record your PD symptoms for 3 consecutive days before your visit.
- Assess your mental health, using a standardised set of questionnaires and tests. As previously outlined, these may contain some questions that some people will find sensitive.

These additional assessments will take between an hour and 2 hours to complete. Therefore the 4 and 5 month post-surgery visits will take1 hour to complete, the 2 month post-surgery visit will take 2 hours and the 3 month post-surgery visit will take 3 hours to complete.

### Follow-up visits 6 months after the surgery

At 6 months after your surgery, you will attend two appointments, at mutually convenient dates and times.

#### Part 1 - John van Geest Centre for Brain Repair (6 hours long)

The first visit will take place at the John van Geest Centre for Brain Repair. You will need to come for this visit OFF medication. Before this visit, you should avoid a heavy meal and eat only a low protein breakfast. Example meals will be discussed with you.

At this visit we will:

- Assess your ability to perform certain **motor assessments**. We will aim to do this first when you are **OFF medication** so you can take your usual anti-PD treatment as soon as possible. You will then be given a soluble form of levodopa and asked to repeat some assessments. After these tasks, you can take your normal medications. Once you feel they are working at their best, you’ll be asked to repeat some tasks. This is so we can assess your movement in both the **OFF** and **ON** state. Some of these assessments will also be filmed. As at your previous visits, you will need to wear the provided cap for the duration of the assessments being filmed.
- Ask how you have felt since your last visit and review all your medications.
- Record your vital signs and weight.
- Perform a physical examination.
- Collect a blood sample of 28 ml, around 2 tablespoons, for routine and safety blood tests. One of the blood tests will check the levels of the **immunosuppressant** tacrolimus (or cyclosporine) in your body. You will therefore need to miss your morning dose of tacrolimus (or cyclosporine) on the day of the visit.
- For participants of childbearing potential only, a urine pregnancy test will be performed.
- Assess your memory and thinking processes using a standardised set of questions and tests (similar to your screening visit).
- Assess your mental health. As previously outlined, these questionnaires may contain some questions that some people will find sensitive.
- Collect your Hauser patient diary card. You’ll be provided with the diary ahead of your visit. As for the previous visits, you will need to record your PD symptoms for 3 consecutive before your visit.
- Perform a repeat **MRI** scan, which will take place at the Wolfson Brain Imaging Centre.
- If you previously consented to the **optional** lumbar puncture, and agree to have this repeated, this will be performed to collect 10 ml, about 2/3 of a tablespoon, of **CSF.**
- If you previously consented to the **optional** additional blood sample for further research, an additional blood sample of 20 ml, between 1 and 2 tablespoons will be collected.

#### **OPTIONAL:** Part 2 - Skåne University Hospital, Lund (3.5 hours long)

It is entirely up to you whether you decide to attend this visit or not. If you decide to go ahead with the visit, you will be made an appointment to undergo a repeat **PET** scan with a **CT** scan at Skåne University Hospital in Lund. As with your screening scans in Lund you will need to be **OFF medication** for the **PET** scan. Before the scan, you should avoid a heavy meal and eat only a low protein breakfast. Example meals will be discussed with you. You should not consume any caffeine (i.e., tea, coffee, and energy drinks) or smoke on the day of the scans.

On the same day as the **PET** scan, you will also have a separate **MRI** scan whilst you are still **OFF medication**. This is also **OPTIONAL.** If for any reason it is not possible to complete the **MRI** scan on the same day as the **PET** scan, it may be performed the following day. Should this happen the trial doctor will discuss with you whether you need to be **OFF** or **ON** your medication for the scan.

You may be accompanied by a member of the trial team from Cambridge, and you may bring a companion (i.e., family member or friend) with you. All travel, accommodation and living costs will be covered.

### Follow up 7 and 8 months after the surgery

For participants of childbearing potential only, a urine pregnancy test will be provided to be taken at home at 7 and 8 months post-surgery. These participants will be contacted by a member of the trial team to confirm the test result.

### Follow-up visit 9 months after the surgery (2.5 hours long)

At 9 months after the surgery, you will be made an appointment, at a mutually convenient date and time, to attend the John van Geest Centre for Brain Repair. You will need to come to these visits **ON** **medication.** At this visit we will:

- Ask how you have felt since your last visit, and review all of your medications.
- Record your vital signs and weight.
- Perform a physical examination.
- Collect a blood sample of 28 ml, around 2 tablespoons, for routine and safety blood tests. One of the blood tests will check the levels of the immunosuppressant tacrolimus (or cyclosporine) in your body. You will therefore need to miss your morning dose of tacrolimus (or cyclosporine) on the day of the visit.
- For participants of childbearing potential only, a urine pregnancy test will be performed.
- Perform a repeat **MRI**. This will take place at the Wolfson Brain Imaging Centre.
- Measure your ability to perform certain **motor assessments**. Some of these assessments will also be filmed. As at your previous visits, you will need to wear the provided cap for the duration of the tasks being filmed.
- Collect your Hauser patient diary card. You will be provided with the diary ahead of your visit. As for the previous visits, you will need to record your PD symptoms for 3 consecutive days before your visit.

### Follow up 10 and 11 months after the surgery

For participants of childbearing potential only, a urine pregnancy test will be provided to be taken at home at 10 and 11 months post-surgery. These participants will be contacted by a member of the trial team to confirm the test result.

### Follow-up visits 12 months after the surgery

At 12 months after the surgery (as at 6 months after the surgery) you will attend for two appointments, at mutually convenient dates and times. At this time point we will discuss tapering and stopping of the trial medications.

#### Part 1 – John van Geest Centre for Brain Repair (6 hours long)

The first visit will take place at the John van Geest Centre for Brain Repair and will be very similar to your visit at 6 months after the surgery. You will also need to come for this visit **OFF medication**. Before this visit, you should avoid a heavy meal and eat only a low protein breakfast. Example meals will be discussed with you.

You will also need to miss your morning dose of tacrolimus (or cyclosporine) on the day of the visit as one of the blood tests will check the levels of this trial medication in your body. In addition to the procedures of the 6 month visit, we will also:

- Ask you to complete questionnaires relating to the non-motor symptoms of PD and quality of life assessments.

#### Part 2 – Skåne University Hospital, Lund (up to 6 hours long, over up to 2 days)

For your second 12 month post-surgery visit you will be made an appointment to undergo a repeat **PET** scan with a **CT** scan at Skåne University Hospital in Lund. As with your screening scans in Lund you will need to be **OFF medication** for your **PET** scan, you should avoid a heavy meal and eat only a low protein breakfast. You should not consume any caffeine (i.e., tea, coffee, and energy drinks) or smoke on the day of the scans. You may be accompanied by a member of the trial team from Cambridge, and you may bring a companion (i.e., family member or friend) with you. All travel, accommodation and living costs will be covered.

**OPTIONAL additional imaging**

Whilst you are in Lund, we would like to perform two additional scans as described below:

- A separate **MRI** scan whilst you are still **OFF medication, o**n the same day as the **PET** scan. If for any reason it is not possible to complete the **MRI** scan on the same day as the **PET** scan, it may be performed the following day. Should this happen the trial doctor will discuss with you whether you need to be **OFF** or **ON** your medication for the scan., you will also have
- A second **PET** scan with a **CT** scan, which would use a different tracer to your first scan, to help light up slightly different activity in your brain. As with the previous **PET** scans, you will need to be **OFF** medication for the PET scan, avoid a heavy meal and eat only a low protein breakfast. You should also not consume any caffeine (i.e., tea, coffee, and energy drinks) or smoke on the day of the scans. This second PET scan would be completed on a separate day to the first scan.

It is entirely up to you whether you decide to have either or both of these additional scans done.

### Follow up 13, 14, 15, 16, and 17 months after the surgery

For participants of childbearing potential only, a urine pregnancy test will be provided to be taken at home at 13, 14, 15, 16, and 17 months post-surgery. These participants will be contacted by a member of the trial team to confirm the test result.

### Follow up visit after completion of immunosuppressants (1 hour long)

Within 2 weeks of finishing treatment with **immunosuppressants**, you will be made an appointment at a mutually convenient date and time, to attend the John van Geest Centre for Brain Repair. You will need to come to these visits **ON** **medication.**

This visit will take place between the 12- and 18- months follow-up visits unless you finish treatment with **immunosuppressants** within 2 weeks of your scheduled 18-month follow-up visit. In this situation this visit will not be required. It is likely this visit will take place after 15+ months post-surgery.

At this visit we will:

- Ask how you have felt since your last visit, and review all of your medications with you.
- Record your vital signs and weight

### Follow-up visit 18 months after the surgery (2.5 hours long)

At 18 months after the surgery, you will be made an appointment, at a mutually convenient date and time, to attend the John van Geest Centre for Brain Repair. You will need to come to these visits **ON** **medication.** At this visit we will:

- Ask how you have felt since your last visit, and review all of your medications with you.
- Perform a physical examination and record your vital signs.
- Collect a blood sample of 22 ml, about 1.5 tablespoons, for routine and safety blood tests.
- For participants of childbearing potential only, a urine pregnancy test will be performed.
- Measure your ability to perform certain **motor assessments**. Some of these assessments will also be filmed. As at your previous visits, you will need to wear the provided cap for the duration of the assessments being filmed.
- Collect your Hauser patient diary card. You will be provided with the diary ahead of your visit. As for the previous visits, you will need to record your PD symptoms for 3 consecutive days before your visit.
- If you previously consented to the **optional** lumbar puncture, and agree to have this repeated, this will be performed to collect 10 ml, around 2/3 of a tablespoon, of **CSF**.
- If you previously consented to the **optional** additional blood sample for further research, an additional blood sample of 20 ml, between 1 and 2 tablespoons will be collected.

### Follow-up visits 24 months after the surgery

At 24 months after the surgery, as with the visits 6 and 12 months after the surgery, you will attend two appointments.

#### Part 1 - John van Geest Centre for Brain Repair (5.5 hours long)

The first visit will take place at the John van Geest Centre for Brain Repair and will be almost identical to your visit at 12 months post-surgery. You will need to come for this visit **OFF medication**. Before this visit, you should avoid a heavy meal and eat only a low protein breakfast. Example meals will be discussed with you. Unlike your 12 month visit:

- There will be **no** recording of your weight.
- There will be **no** pregnancy test for participants of childbearing potential.
- There is **no** repeat lumbar puncture or additional blood sample for research for those who previously consented to the **optional** procedures.

#### **OPTIONAL:** Part 2 – Skåne University Hospital, Lund (up to 6 hours long, over up to 2 days)

It is entirely up to you whether you decide to attend this visit or not. If you decide to go ahead with the visit, you will be made an appointment to undergo a repeat **PET** scan with a **CT** scan at Skåne University Hospital in Lund. As with your previous scans in Lund; you will need to be **OFF medication** for your **PET** scan, you should avoid a heavy meal before and eat only a low protein breakfast. You should not consume any caffeine (i.e., tea, coffee, and energy drinks) or smoke on the day of the scans. You may be accompanied by a member of the trial team from Cambridge, and you may bring a companion (i.e., family member or friend) with you. All travel, accommodation and living costs will be covered.

Whilst you are in Lund, we would like to perform two additional scans as described below:

- A separate **MRI** scan whilst you are still **OFF medication, o**n the same day as the **PET** scan. If for any reason it is not possible to complete the **MRI** scan on the same day as the **PET** scan, it may be performed the following day. Should this happen the trial doctor will discuss with you whether you need to be **OFF** or **ON** your medication for the scan., you will also have
- A second **PET** scan with a **CT** scan, which would use a different tracer to your first scan, to help light up slightly different activity in your brain. As with the previous **PET** scans, you will need to be **OFF** medication for the PET scan, avoid a heavy meal and eat only a low protein breakfast. You should also not consume any caffeine (i.e., tea, coffee, and energy drinks) or smoke on the day of the scans. This second PET scan would be completed on a separate day to the first scan.

All of the scans at Skåne University Hospital at this visit are optional. It is entirely up to you whether you decide to complete any or all of these additional scans.

### Follow-up visit 30 months after the surgery (1.5 hours long)

At 30 months after the surgery you will be made an appointment, at a mutually convenient date and time, to attend the John van Geest Centre for Brain Repair. This visit will be almost identical to your visit at 18 months. The differences between the visits are as follows:

- There will be **no** pregnancy test for participants of childbearing potential.
- There will be **no** repeat lumbar puncture for those who previously consented to the **optional** procedure.
- There will be no repeat **optional** additional blood sample for further research

### Follow-up visits 36 months after the surgery

At 36 months after the surgery, you will attend two appointments.

#### Part 1 - John van Geest Centre for Brain Repair (6 hours long)

The first visit will take place at the John van Geest Centre for Brain Repair and will be almost identical to your visit at 24 months post-surgery. You will need to come for this visit **OFF medication**. Before this visit, you should avoid a heavy meal and eat only a low protein breakfast. Example meals will be discussed with you. Unlike the visit at 24 months:

- There will be more assessments to test your memory and thinking processes than at your 24-month visit.
- You will also have an **ECG**.
- If you previously consented to the **optional** additional blood sample for further research, an additional blood sample of 20 ml, between 1 and 2 tablespoons will be collected.

#### Part 2 –Region Skåne – Skåne University Hospital (6 hours long, over 2 days)

For your second 36 month visit after surgery you will be made an appointment to undergo two repeat **PET** scans at Region Skåne – Skåne University Hospital over two days. These scans will be combined with a **CT** scan. You can bring a companion with you if you wish. All travel, accommodation and living costs will be covered. You might also be accompanied by a member of the trial team from Cambridge. Like the previous **PET** scans in Lund, you will need to be **OFF medication** for the **PET** scans. Before the scan you should avoid a heavy meal and eat only a low protein breakfast. You should also not consume any caffeine (i.e., tea, coffee, and energy drinks) or smoke on the day of the scans. After your **PET** scan, you will also have a separate **MRI** scan whilst you are still **OFF** **medication**. If for any reason it is not possible to have the **MRI** scan on the same day as either **PET** scan, it may be performed on a separate day. Should this happen the trial doctor will discuss with you whether you need to be **OFF** or **ON** your medication for the scan.

### Enrolment in long term follow up study

After the 36 month follow up visits, you will have completed all your follow-up visits for the STEM-PD trial. However, we want to continue to monitor you for life. You will therefore be asked if you are willing to consent to a separate long-term follow-up trial. This will be covered by a separate patient information sheet and informed consent form and will be similar to that which you have done before as part of the TransEUro observational study.

### Schedule of trial procedures

| **ASSESSMENT** | Screening | Baseline | Pre-surgery | Day before surgery | Surgery  Day 0 | Days | | | | | | |  | Months | | | | | | | | | | |
| --- | --- | --- | --- | --- | --- | --- | --- | --- | --- | --- | --- | --- | --- | --- | --- | --- | --- | --- | --- | --- | --- | --- | --- | --- |
| Visit |  |  |  |  |  | 1 | 2 | 7 | 14 | 21 | 28 | 42 | 2 | 3 | 4 | 5 | 6 | 9 | 12 | 15+ | 18 | 24 | 30 | 36 |
| Medication state | OFF | |  | | | ON | | | | | | | | | | | OFF | ON | OFF | ON | ON | OFF | ON | OFF |
| You agree to participate | X |  |  |  |  |  |  |  |  |  |  |  |  |  |  |  |  |  |  |  |  |  |  |  |
| Discuss your health and medication | X | X | X |  | X | X | X | X | X | X | X | X | X | X | X | X | X | X | X | X | X | X | X | X |
| Height | X |  |  |  |  |  |  |  |  |  |  |  |  |  |  |  |  |  |  |  |  |  |  |  |
| Weight | X | X |  | X |  |  |  | X | X | X | X | X | X | X | X | X | X | X | X | X |  |  |  |  |
| **ECG** |  | X |  |  |  |  | X |  |  |  |  |  |  |  |  |  |  |  |  |  |  |  |  | X |
| Vital signs |  | X |  |  | X | X | X | X | X | X | X | X | X | X | X | X | X | X | X | X | X | X | X | X |
| Physical examination | X | X |  |  |  |  |  | X | X | X | X | X | X | X | X | X | X | X | X |  | X | X | X | X |
| Pregnancy test^1^ | X | X |  | X |  |  |  |  |  |  | X |  | X | X | X | X | X | X | X |  | X |  |  |  |
| Blood tests | X |  | X | X^4^ |  | X | X | X | X | X | X | X | X | X | X | X | X | X | X |  | X | X | X | X |
| **MRSA** swab | X |  |  |  | |  |  |  |  |  |  |  |  |  |  |  |  |  |  |  |  |  |  |  |
| **Optional** research blood sample |  | X |  |  |  |  |  |  |  |  |  |  |  |  |  |  | X |  |  |  | X |  |  | X |
| **Optional** lumbar puncture for **CSF** & blood sample (where required) collection |  | X |  |  |  |  |  |  |  |  | X |  |  |  |  |  | X |  | X |  | X |  |  |  |
| **MRI** scan (in Cambridge) | X |  |  |  |  |  |  |  |  |  | X |  | X | X |  |  | X | X | X |  |  | X |  | X |
| **MRI** scan (in Lund) |  |  | X |  | X |  | X |  |  |  |  |  |  |  |  |  |  |  |  |  |  |  |  |  |
| **Imaging** in Lund^3^ | X |  | X^5^ |  |  |  |  |  |  |  |  |  |  |  |  |  | X^2^ |  | X |  |  | X^2^ |  | X |
| **Motor assessments** | X | X |  |  |  |  |  |  |  |  |  |  |  | X |  |  | X | X | X |  | X | X | X | X |
| Hauser patient diary card |  | X |  |  |  |  |  |  |  |  |  |  |  | X |  |  | X | X | X |  | X | X | X | X |
| Assessments of your memory and thinking processes | X | X |  |  |  |  |  |  |  |  |  |  |  |  |  |  | X |  | X |  |  | X |  | X |
| Assessments of your mental health | X | X |  |  |  |  |  |  |  |  | X |  |  | X |  |  | X |  | X |  |  | X |  | X |
| Assessments of other PD symptoms and quality of life |  | X |  |  |  |  |  |  |  |  |  |  |  |  |  |  |  |  | X |  |  | X |  | X |
| **TREATMENT** | | | | | |  |  | | | | | | | | | | | | | | | | | |
| Surgery in Lund |  |  |  |  | X |  |  |  |  |  |  |  |  |  |  |  |  |  |  |  |  |  |  |  |
| Other medications^6^ |  |  |  |  | |  | **As prescribed** | | | | | | | | | | | | | | |  |  |  |

^1^For participants of childbearing potential only. Monthly urine pregnancy tests will be performed by participants at home at months 7, 8, 10, 11, 13, 14, 15, 16 and 17.

^2^This visit is optional

^3^This can include **PET/CT** scans and **MRI** scans

^4^ Blood samples will not be repeated if already performed within 3 days prior to surgery

^5^Completed **OFF medication**

^6^**Immunosuppressants**, antibiotics, medications to reduce the risk of **osteoporosis**.

## What are the side effects of the treatment being tested?

**STEM-PD** is an investigational product, and this trial is the first time it has been used in people. The side effects of this treatment are unknown and it is possible that some participants may do better than others. It is important that you tell your trial doctor and/or trial staff about any side effects or symptoms you may have during the trial, whether or not you think they are related to **STEM-PD**.

In previous studies, where **dopamine** cells from **foetal tissue** were transplanted into the brain of people with PD, some patients developed new or worsening symptoms called graft-induced dyskinesias (**GIDs**). These involuntary movements are similar to the abnormal movements most people with PD get after prolonged exposure to levodopa/L-dopa (i.e., sinemet/madopar tablets); however, in this case, the abnormal movements were driven by the transplant and not by medications. In a few cases, patients required additional surgery called **deep brain stimulation**, where a pulse generator (a device like a heart pacemaker) is placed under the skin around the chest or stomach area. This is connected to one or two fine wires that are inserted into specific areas of the brain, and is able to deliver high frequency stimulation, which changes some of the electrical signals in the brain that cause some of the motor problems seen in PD. **Deep brain stimulation** is the main type of surgery normally used nowadays to treat PD when medications no longer control a person’s symptoms. We believe that compared to transplants involving **foetal tissue**, the risk of this side effect is reduced with **STEM-PD.** However, there is still a risk that patients who undergo transplantation in this trial may develop these involuntary movements following the surgery. If this happens the research team will follow standard processes for managing these, which may eventually include **deep brain stimulation**.

**STEM-PD** is made from **stem cells** that can develop into many different cell types, including brain cells producing **dopamine**. Although the cells in **STEM-PD** no longer have the ability to develop into other cell types, any residual **stem cells** could in theory give rise to uncontrolled cell growth or lead to the development of a teratoma (a tumour composed of tissues not normally present at the site). No teratoma formation, abnormal growth or tumours have been detected in any of the thorough pre-clinical testing of the **STEM-PD** cells. We therefore consider the risk of teratoma formation, abnormal growth or tumours to be extremely small.

It is also possible for the transplanted cells to move from the transplantation site to other areas of the brain. If this happens, the transplanted cells could disrupt the function of other important behaviour and movement pathways. However, there has been no sign of migration of the transplanted cells to other sites in any of the pre-clinical testing.

## What are the possible disadvantages and risks of taking part?

There is a substantial time commitment associated with being part of the trial. It will involve multiple visits to the hospital, and trips to Sweden. As well as being time consuming, there are some potential risks associated with the treatments, as documented above.

Other risks associated with trial procedures are listed below.

### Possible risks relating to the surgery

Any surgical intervention involves a risk. All local standard procedures will be followed to minimise the risks attached to surgery. Potential risks and side-effects that could be associated with surgery are listed below.

#### Possible anaesthesia risks

- During the surgery, you will receive general anaesthesia. Anaesthesia is very safe, but there are risks with any medicine. Minor side effects of anaesthesia and ventilating you whilst anaesthetised, such as a sore throat, nausea, and vomiting, are common.
- Major complications from anaesthesia are rare. If you have any concerns, please ask your anaesthesiologist. Serious complications, involving the general anaesthetic, which are rare, may affect any of the following body systems: heart, breathing, neurological, or immune systems (allergies or infections).
- The surgery is expected to take up to 10 hours. The long general anaesthesia time increases the risk of lower blood flow to the brain. To mitigate this risk, your blood pressure will be closely monitored throughout the surgery and all other standard processes will be followed. Furthermore, neurosurgery operations of this length are not uncommon at Skåne University Hospital in Lund and Addenbrooke’s Hospital in Cambridge.
- The anaesthetic used during the surgery may render your brain more sensitive to some anti-PD medications. There is therefore a higher risk of transient confusion and hallucinations after the long surgery times. This confusion and/or hallucinations are usually temporary and pass after a few days. Your medication will be managed by the trial doctor before, during and after surgery to reduce the risk of these side effects.
- After your surgery you may feel sick or nauseous or you may vomit. This is a common side effect of an anaesthetic.

#### Possible risks of the **NG tube**

- There is a risk that the **NG tube** can accidentally go into the lungs rather than the stomach when it is being inserted where it would be unable to support the stomach. The **NG tube** will be placed by experienced clinician and its position verified.

#### Possible risks associated with transplantation

- There is a small risk that bleeding may occur in the brain during the operation (up to 1 in every 500 operations). If this occurs, blood can sometimes compress the brain, requiring a further operation.
- Direct injections into the brain very slightly increases the risk of epileptic fits in the immediate period post-transplant and leakage of fluid around the brain (**cerebrospinal fluid**) which can cause headaches.
- Transplantation of cells into the body carries a small risk of passing on an infection. **STEM-PD** has been thoroughly tested and found to be sterile. Handling of the cells will be limited and performed under sterile and controlled conditions. You will also receive antibiotic treatment, as is standard for this type of surgery, and therefore the risk of infection from the transplant is minimal.

#### Possible other risks

- You may notice painful redness around the vein into which any infusions/medicinal products have been infused.
- The introduction of a tube into the wind pipe (intubation) to help with breathing during the surgery may result in throat soreness or hoarseness after the operation.
- In some cases, the intubation can also cause damage to your teeth and mouth. To reduce the risk of this, you must inform medical staff of any false teeth or specific dental fragility.
- The prolonged position on the operating table may cause compressions, particularly to certain nerves, which may result in numbness or, in exceptional cases, paralysis in the arms or legs. This typically resolves anywhere between a few days to a few weeks.
- Accidents linked to the passage of vomit into the lungs are very rare, especially if you fast as instructed.

### Possible risks relating to other trial procedures

Due to the amount of trial visits, there may be inconveniences in travel to and from your trial visits. Listed below are the other risks and discomforts that may be associated with the trial procedures.

#### Immunosuppressive treatment

All medications have risks and potential side effects. The common risks and side effects for **immunosuppressants** in general are listed below:

- Long-term use of **immunosuppressants** weakens the immune system, increasing your risk of developing infections. You can reduce your risk of developing new infections by following careful hygiene rules when handling food and interacting with infectious people. You will be given instructions on how to let the trial team know of any side effects that you experience, to allow for early and effective treatment if needed. To help reduce the risk of developing new infections, you will be given long-term antibiotics for the duration of the **immunosuppressant** treatment.
- Long-term **immunosuppressant** use could put you in a higher risk category for infections, including COVID-19. As long as you are fully vaccinated against COVID-19 prior to start of the trial **immunosuppressants** (irrespective of which type of vaccine), the protection of the vaccination will not be diminished with **immunosuppressant** treatment. If you are not fully vaccinated, there is an increased risk of complications of COVID-19 infection. If vaccination takes place during the period of immunosuppression it will be less effective. Prior vaccination will provide adequate protection against a severe course of COVID-19. People with PD are otherwise not at risk for getting more severe COVID-19 infections.
- In the early stage of the trial, higher doses of prednisolone will be used, meaning there is a higher chance that side effects may develop. Possible side-effects can include diabetes, skin thinning, mania, psychosis, confusion and other neurological symptoms; however, these occur in a small number of patients.
- Use of **immunosuppressants** can allow dormant infections to manifest. During the screening process we will check for risk factors including potential sites for dormant infections and review your blood test results for evidence of previous exposure to certain viruses. If we identify any concerns during the screening that would put you at a higher risk, you will not be included in the trial.
- Taking **immunosuppressants** increases the risk of certain malignancies including skin cancers. The risk increases with the presence of certain viruses, which we will check for during the screening process. The relatively short length of time that the **immunosuppressants** are being used in this trial (less than 15 months) means that this risk is greatly reduced.
- **Immunosuppressants** increase the risk of **osteoporosis**, which can be prevented with the use of other medications. As part of this trial, you will be given other medications (therefore reducing this risk), as detailed earlier in this document. Long-term use also increases the risk of developing type II diabetes. We will closely monitor this throughout the trial, including by regular blood tests.
- When you begin to reduce/stop **immunosuppressants** there is a risk that your immune system may reject the transplanted cells and with it you may lose any of its benefits. This is much less likely to occur than with a whole organ transplant. The length of **immunosuppressant** treatment used in this trial is informed by the previous research with **foetal tissue** transplants. In the event of a suspected rejection, you may be treated with high-dose **immunosuppressants** in the first instance.

#### Antibiotic treatment

Antibiotics have potential risks and side effects like all medications. The common risks and side effects for antibiotics in general are listed below:

- A common side effect of some antibiotics is diarrhoea and/or nausea. These types of gastrointestinal issues could lead to lower absorption of medication. These side effects are most common for a particular type of antibiotic which will not be used in the trial. Furthermore, the antibiotics used in this trial are the same type as have been used in previous transplants using **foetal tissue**, without patients needing to adjust their normal medications.
- Overuse and ineffective use of antibiotics can lead to antibiotic resistance. The antibiotic treatment regime used in this trial has been used for several hundred thousand organ transplant patients worldwide and has been shown to lead to minimal antibiotic resistance.

#### Temporarily stopping your PD medication for some assessments (**OFF** **medication** state)

On several occasions we will ask you to stop taking your usual anti-PD medication for at least 12 hours, or longer for long-acting medications. This is to assess some of the features of your PD in the **OFF** state. Going without your normal PD medication will temporarily worsen the symptoms of your disease. As soon as the assessments have been done, you will be given a dose of your normal PD medication. As missing this medication can be difficult for some patients, trial visits will be scheduled for the morning wherever possible. If it is impossible for you to travel to the trial visit whilst **OFF medication**, we may be able to arrange overnight accommodation for you and your companion if appropriate at or near the hospital.

#### Blood samples

You will have multiple blood tests over the duration of the trial. None of the blood tests in the trial will be fasting blood tests unless the trial doctor feels it is clinically necessary. If required, a fasting blood test means you will be told not to eat or drink anything (other than water) beforehand (typically from midnight before you attend).

The blood tests may cause mild discomfort and bruising of the skin. Standard protocols will be followed to prevent infections. Having a blood sample taken can be uncomfortable when the needle goes into your skin and may cause infection, bruising, redness or swelling.

#### **Optional** lumbar puncture

A possible side effect of a lumbar puncture is a headache, usually coming on within 48 hours. The chance of this is said by NHS-Choices to be 1 in 4, but more recent studies using modern methods show much lower rates, of around 1 in 20 people. The risk may be reduced by drinking plenty of fluids (a litre of water a day until the following day). If a headache does develop, it usually responds well to resting in bed, paracetamol and caffeine-containing drinks. For the lumbar puncture itself, we use local anaesthetic, but there may still be a pin-prick sensation in the lower back at the time, or mild tenderness or pain afterwards which settles after a short time. Occasionally there may be a little bleeding from the puncture site as sometimes happens after a routine blood sample is taken. There is a small risk of infection from a lumbar puncture as the needle breaks the skin's surface, providing a possible way for bacteria to enter the body. This is minimised by following the hospital’s standard procedures. Rarely, some bruising or swelling at the site may occur. Serious complications after a lumbar puncture are extremely rare using the method we are adopting, which is standard in our hospitals. These rare complications include tingling and numbness in the legs, infection in the spine, double vision, tinnitus and hypersensitivity to light or sound, but these are so rare as to be unquantifiable.

#### **MRI** scans

**MRI** scans involve a large magnet which is used to create a number of different types of images of your internal organs, from ones that show extremely high anatomical detail, to ones that tell us how well the organs are functioning. Unlike **PET** and **CT** scans, MRI scans do not involve any exposure to ionising radiation.

Though **MRI** scanning is generally very safe, there are certain circumstances where it must be avoided, as such preventing you from taking part in this type of scan. We will go through a checklist to ask whether you have metal objects attached to or inside your body (e.g. stents, shrapnel, plated fractures, piercings, tattoos) or electronic devices (e.g. heart pace-maker). Many such items (most modern cardiac stents, for instance) have been tested in MRI scanners and provided that scanning staff are given full details of the implant in advance of the scan these ca often be scanned safely. We will also ask you to remove jewellery and any make-up as these can sometimes contain metallic pigments. For the **MRI** scans, you will be asked to lie as still as you can in a small and enclosed space in the **MRI** machine for about 30 minutes. You may find this claustrophobic. The scanner is noisy, but you will be given earplugs and/or headphones. For some visits, you may ask to have your choice of music played over headphones if you wish. The technician performing the **MRI** scan will communicate with you throughout the scan to check whether you remain comfortable. If for any reason you cannot continue then the scan can be stopped at any point. Where possible, we will schedule the scan on the same day as the other tests, but there may be occasions where this is not possible if the scanner is fully booked or is unexpectedly unavailable.

Some of the **MRI** sequences used during the course of this study may be classified as research sequences and we may use an aerial (radiofrequency coil) to collect the data that is not provided by the manufacturer. This means that the **MRI** scanner will be used in a different way to what is standard by the manufacture (we call it “off-label”). Using the scanner in this way could help to reduce the scan time and provide better quality or more useful image data for the research study.

Like faces, brains come in all shapes and sizes, so that there are many normal variations of what the scan shows. There is a chance of less than 1:100 that your **MRI** scan may show a significant abnormality of which you are unaware. If this should happen, we will notify you and ask for your consent to contact your GP directly if appropriate, to ensure you get the correct support and advice. Such early detection has the benefit of starting treatment early but, in a small number of cases, may have implications for future employment and insurance.

At least one of your **MRI** scans will be performed under a general anaesthetic in order to generate a higher resolution image of your brain. The scan is likely to take less than 30 minutes and is a low risk procedure. For possible anaesthetic related side-effects, see the relevant section above: possible risks relating to the surgery.

#### **PET** scans

**PET** scans involve a radioactive liquid, called a radioligand or a tracer, which is injected into your bloodstream to help light up certain areas in your body to allow better pictures to be taken. The tracer has a short-lived radioactivity and there are no precautions for you to take after the scan has been carried out. Like all medicines, injected tracers can be associated with some side effects. Side effects are rare and often relatively minor and short-lived but can include nausea, rash, abdominal discomfort, cold flushes and dizziness. Severe reactions are possible but are very rare and have never occurred in any of our own studies.

All of the **PET** scans in this trial will be combined with a **CT** scan. This type of scan uses X-rays to generate images which exposes you to additional radiation.

If you take part in this trial you will have between 5 and 9 **PET** scans as some of the scans are optional. These will be extra to those that you would have if you did not take part in the trial. These procedures use ionising radiation to form images of your body. Ionising radiation may cause cancer many years or decades after the exposure. We are all at risk of developing cancer during our lifetime. 50% of the population is likely to develop one of the many forms of cancer at some stage during our lifetime. Taking part in this trial, with the maximum number of PET scans, would increase the chances of this happening to an otherwise healthy adult by an additional 0.2%.

#### CT angiography

Angiography is a type of X-ray used to check blood vessels. Blood vessels do not show clearly on a normal X-ray, so a special dye called a contrast agent needs to be injected into your blood first. A small cut will be made over a blood vessel and a very thin flexible tube will be inserted. This tube is used to deliver the contrast agent into the blood vessels that lead to the brain. Local anesthetic will be used to numb the area where the cut is made. Angiography is generally a safe and painless procedure, but for a few days or weeks afterwards it's common to have bruising, soreness, and/or a very small lump or collection of blood near where the cut was made.

As this type of imaging uses x-rays, you will be exposed to a small amount of ionising radiation. The amount of radiation is equivalent to around 8 months of average background radiation for people living in the UK.

#### Assessments/questionnaires

Some of the questions in the assessments/questionnaires are of a personal nature and will involve discussion of sensitive topics such as depression and suicide. You may find them upsetting or embarrassing. They will only be carried out by trained individuals from the trial team who will discuss any issues that arise from these with you. Following consultation, you may be referred to your GP or other professional, if appropriate, and if this is in line with your wishes.

#### Incidental findings

In some cases, we may discover something about your health that you are unaware of, for example an abnormal finding on a blood test or scan. If this should happen, we will notify you and ask for your consent to contact your GP directly if appropriate, to ensure you get the correct support and advice.

## What are the possible benefits of taking part?

There is no guarantee that you will benefit from taking part in this trial. However, information collected as part of your participation in this trial may be of huge benefit to PD research and people with PD in the future.

The main purpose of this trial is to test the safety of the **STEM-PD** cells. This is the first time the **STEM-PD** cells will be given to humans meaning that it is unknown if it will have a beneficial effect.

## What are the alternatives for treatment?

Currently no treatment is available for slowing down the progression or repairing the causes of PD. Medications are available to treat the symptoms of PD, which you are already taking. We do not plan to alter or change these medications if you decide to participate in the trial unless there are clinical reasons for doing so as would be the case normally in the management of your PD.

## What happens when the trial stops?

After this trial stops, your treatment will carry on as normal, you will return to the care of your current consultant neurologist/physician. We would like to continue to monitor you for life when the trial ends and we will ask you to consent to a separate long-term follow-up study, which will be covered by a separate patient information sheet and informed consent form. This long-term follow-up study will enable the collection of high-quality data on **STEM-PD** for an extended period of time. This will be vital in determining the longer-term safety and the durability of the potential benefit of **STEM-PD**.

## Expenses & payment?

You will not receive any payment for participating in this trial.

We will arrange and pay for all travel, accommodation and living costs for your travel to Lund, Sweden. Travel insurance will be arranged for travel to Lund for surgery and **PET** imaging.

It is important that you keep any travel-related receipts and request a claim form at your hospital visit, if appropriate. Details of how and when payments will be made are available from the trial team.

We can reimburse any reasonable travel, parking and living costs incurred by your participation in this trial that are not already paid for directly by the trial team (i.e., travel to Lund, overnight accommodation, etc.). Parking is available free of charge at the John van Geest Centre for Brain Repair during your visits here.

All payments are made electronically via BACS payment, and so in order to process any travel claims we will need your bank account number and sort code. These will be kept confidential and only used for the purpose of reimbursing you for your travel expenses.

# Section 2: Trial Conduct

## Who is the Sponsor for the trial?

Region Skåne – Skåne University Hospital is the Sponsor for this clinical trial. The Sponsor has overall responsibility for the set-up, management, and reporting of the data from the trial. Contact details for the Sponsor are as follows:

Region Skåne – Skåne University Hospital, Region Skåne: SE-291 89 Kristianstad, Sweden. Tel: +46 44-309 30 00

## What if new information becomes available?

Sometimes during the course of a trial, new information becomes available which might affect your decision to continue participating in this trial. If this should happen, your trial doctor will contact you to discuss the new information and whether you wish to continue participating in the trial. If you still wish to continue in the trial, you will be asked to sign a new informed consent form.

The trial Sponsor organisation, the regulatory authorities, or the trial doctors may decide to stop the trial at any time. If this happens, we will tell you why the trial has been stopped and arrange for appropriate care and treatment for you.

## What if I decide I no longer wish to participate in the trial?

You are free to leave this trial at any time without giving a reason and without it affecting your future care or medical treatment. No further tests will be performed on you and no further research samples will be collected. Any data already collected or results from tests already performed on you or your samples will continue to be used in the trial analysis. If you withdraw, you will be given the option to choose for your un-processed research samples to be destroyed.

If you choose to withdraw after the surgery, we will ask for your consent to contact your neurologist/physician or GP. This is to allow us to monitor for any potential side effects relating to the **STEM-PD** cells, which is important in a first in human trial. We will also ask for your consent to access and use ongoing clinical data concerning your PD. In this case, you will be given contact details of the trial team in the event that any problems arise relating to your participation in this trial.

Should you withdraw from the trial after the surgery in a period where you are still taking medications provided as part of the trial (i.e., in the first 15 months post-surgery) you should continue to take the medications provided as instructed.

The trial doctor may also choose to withdraw you from the trial if they feel it is in your best interests or if you have been unable to comply with the requirements of the trial. Reasons for trial withdrawal could include:

- Being unable to complete your trial visits or assessments as required.
- The trial doctor feels you are no longer suitable for the surgery.

## What if there is a problem?

Any complaint about the way you have been dealt with during the trial or any possible harm you might have suffered will be addressed. If you have any concerns about any aspect of this trial, you should speak to your trial doctor who will do their best to answer your questions.

In the event that something does go wrong, and you are harmed by taking part in this trial and this is due to someone’s negligence then you may have grounds for a legal action for compensation against Cambridge University Hospitals NHS Foundation Trust, University of Cambridge, Lund University or Region Skåne – Skåne University Hospital. The normal National Health Service complaints mechanisms will still be available to you (if appropriate). The University of Cambridge has obtained insurance which provides no-fault compensation i.e., for non-negligent harm, you may be entitled to make a claim for this***.***

The NHS does not provide no-fault compensation i.e., for non-negligent harm, and NHS bodies are unable to agree in advance to pay compensation for non-negligent harm. They are able to consider an ex-gratia payment in the case of a claim.

Procedures in Sweden are covered by the Patient Injury Act, the patient insurance responsible for heath and care in Sweden. This insurance covers the surgery and any assessments that will take place in Lund and any harm that could be caused through negligence of employees of Region Skåne – Skåne University Hospital, or any harm caused where no legal liability arises. You will be covered by this insurance for the duration of your stay in Sweden. This includes any problems that may arise after you have returned to the UK, if relating to actions at RS-SUS.

If you wish to complain or have any concerns about any aspect of the way you have been approached or treated during this trial, you can do this through the NHS complaints procedure. In the first instance it may be helpful to contact the Patient Advice and Liaison Service (PALS) at Addenbrooke’s hospital.

## How will we use information about you?

Region Skåne – Skåne University Hospital is the Sponsor for this clinical trial based in the United Kingdom and Sweden. The University of Cambridge will act as the data processor for the trial, with Region Skåne – Skåne University Hospital, who are responsible for looking after your information and using it properly, acting as the data controller for this trial.

We will need to use information from you and your medical records for this trial. This information will include:

- your name
- NHS/hospital number
- date of birth
- sex
- contact details
- height and weight
- GP contact details

People will use this information to do the research or to check your records to make sure that the research is being done properly. We will keep all information about you safe and secure. The Sponsor will keep identifiable information about you for 30 years after the trial has finished to ensure your safety and allow the trial to be reviewed by the authorities after it is finished.

Information about you will be securely transferred between the trial team at different trial sites in relation to your participation in this trial. Where your information is transferred outside of the UK, we expect our rules about keeping your information safe to be followed.

Your imaging data generated at the Wolfson Brain Imaging Centre will be kept for a minimum of 10 years after collection, and possibly indefinitely. Your imaging data will be stored on secure computer systems with data encryption according to the policies of the Wolfson Brain Imaging Centre, University of Cambridge. This data will contain your personal information (name, age, height and weight), however only authorised researchers involved in the trial will have access to view identifiable data.

Images generated from the PET and MRI scans in the trial will be **pseudoanonymised** and shared between Addenbrooke’s hospital, Region Skåne – Skåne University Hospital, Imperial College London, and other members of the trial team, to allow for analysis of the imaging.

In order to arrange your surgery and **PET** imaging in Sweden a copy of your informed consent form and data including your name, date of birth, sex, address, height, and weight will be stored on secure network drives hosted by Cambridge University Hospital NHS FT or the University of Cambridge Clinical School’s Secure Server, which is ISO 27001 certified, and conforms with NHS Information Governance Toolkit and NHS Data Security Protection Toolkit. These data will be shared with authorised members of the trial team at both sites via a two-factor authentication system provided by the University of Cambridge Secure Research Computing Platform (SRCP). Access will be restricted to authorised members only.

People who do not need to know who you are will not be able to see your name or contact details. Your data will have a code number instead.

Video recordings of the trial assessment detailed in section 8 will be labelled with a code number unique to you, the name of the assessment recorded and where applicable the date. It will not be possible to obscure you face in these videos, as a clear recording of your face is needed to accurately score the assessment. Recordings will be kept for 30 years after the end of the trial.

**Pseudoanonymised** **data**, including safety data such as details of adverse events, may be shared with the manufacturer of **STEM-PD**, the Royal Free Hospital in London, and the funder Novo Nordisk. This data will allow the manufacturer and funder to evaluate the safety of STEM-PD and may inform future clinical trials using **STEM-PD**. **Pseudoanonymised** **data** has had information that can identify a person removed and replaced with a reference or ID number but may include information such as your date of birth. An individual can only identify a person from **pseudoanonymised** **data** when they have access to additional relevant information. The only people who will have access to information that identifies you will be people who need to contact you in relation to this trial or to audit the data collection process.

We will need to inform your GP of your participation in this trial so that any medical decisions made by your GP account for any treatment you are receiving as part of this trial.

Once we have finished the trial, we will keep some of your information so we can check the results. We will write our reports in a way that no-one can work out that you took part in the trial.

De-identified information about your health and care relevant to this trial may be made available for other research studies run by Cambridge University Hospitals, Lund University, Skåne University Hospital, and/or the University of Cambridge. These organisations may be NHS or other public sector organisations, academic institutions, charities and commercial companies in the UK or abroad. Before your data is shared with other organisations all personal identifiers, such as names, addresses and dates of birth, will be removed. Making information from trials available for further research helps maximise the benefit of conducting trials and allows other researchers to verify results and avoid duplicating research. To facilitate this, some trial datasets are made available to researchers via a public online database and become “open data”. Data are thoroughly de-identified before they are submitted to an open data platform and once the data are uploaded, we do not have control over how they are used.

- 1. **What are your choices about how your information is used if you change or stop your participation in the trial?**
- You can stop your participation in this trial at any time, without giving a reason, but we will keep information about you that we have already collected.
- If you choose to stop taking part in the trial, we would like to continue collecting information about your health from your neurologist/physician or GP. We will ask for your consent to do this.
- We need to manage your records in specific ways for the research to be reliable. This means that we will not be able to let you see or change the data we hold about you.

### Where can you find out more about how your information is used?

You can find out more about how we use your information below

- at <https://www.skane.se/en/support-pages/how-we-process-your-personal-data/>
- at [www.hra.nhs.uk/information-about-participants/](http://www.hra.nhs.uk/information-about-patients/)
- our leaflet available from [www.hra.nhs.uk/participantdataandresearch](http://www.hra.nhs.uk/patientdataandresearch). Alternatively, please visit, for Cambridge University Hospitals NHS Foundation Trust: [https://www.cuh.nhs.uk/participant-privacy/](https://www.cuh.nhs.uk/patient-privacy/). For University of Cambridge: <https://www.medschl.cam.ac.uk/research/information-governance> or email The Information Governance team at: [researchgovernance@medschl.cam.ac.uk](mailto:researchgovernance@medschl.cam.ac.uk)
- by asking one of the research team refer/to/add contact details
- by sending an email to [gdpr.enquiries@addenbrookes.nhs.uk](mailto:gdpr.enquiries@addenbrookes.nhs.uk), or
- contact the Data Protection Officer at: Region Skåne, SE-291 89 Kristianstad, Sweden. Tel: +46 44-309 30 00, or e-mail at: [region@skane.se](mailto:region@skane.se)

## What will happen to my samples?

Only members of the authorised trial team, and certain individuals from the Sponsor and regulatory organisations who may need to check the accuracy of this trial, will have access to the collected trial samples. Samples will be labelled with your Participant ID number.

The blood samples collected over the duration of the trial will be analysed as per standard practice in the routine NHS Biochemistry, Haematology and Microbiology laboratories of Addenbrooke’s Hospital or the Department of Laboratory Medicine at Region Skåne – Skåne University Hospital. As is the standard care processes at Addenbrooke’s Hospital, some samples will be sent to Sandwell and Birmingham Hospital for analysis.

Where you have consented to the **optional** research blood sample, samples collected from you will be analysed and any remaining samples will be retained for future research in the secure freezers at the John van Geest Centre for Brain Repair.

Where you have consented to the **optional** **CSF** collection, analysis of this will be performed on site as per standard practice in the routine NHS laboratories at Addenbrooke’s hospital. Your samples will also be analysed for immunological responses and any remaining sample may be stored in the secure freezers at the John van Geest Centre for Brain Repair for future research.

At the end of the trial, we will ask for ethical approval to use any unused samples in another project. Samples will only be retained if we have appropriate ethical approval to do so. Some of these samples may be shared with other research centres who are doing very similar research to us, and which has been ethically approved. Research data collected during the trial may also be shared in a similar way. Such data and sample sharing allows information from larger groups of participants from different sites to be analysed all together, which makes the results more reliable. Sharing samples can also allow more specialised tests to be done which are relevant to the research, but that we might not be able to do locally. Sharing of research data and samples may take place both within and outside the UK and EEA, including the USA, where data protection laws are less strict. However, any research data or samples leaving Cambridge University Hospitals NHS Trust/University of Cambridge will be fully anonymised; your personal details (including your name and address) will be removed and replaced with a trial-specific code so that you cannot be recognised. We will ask you on the consent form if you are happy for your anonymised data and samples to be shared in this way with researchers working in collaboration with us on similar ethically approved studies.

Any samples you donate will be treated as “gifts” to the University of Cambridge, Department of Clinical Neurosciences. This means that the department will have control over what happens to the samples, how they are used and all rights to any “inventions” (such as medications or tests) which might come out of research performed using your samples.

## How will results of the trial be published?

We would like to keep you informed about the progress of the trial. If you do not want this, please indicate on the consent form at the end of this document. When the results of this trial are available, they may be published in peer reviewed medical journals and used for medical presentations and conferences. They will also be published in an approved, publicly accessible online Clinical Trials Register. The results of the trial will be anonymised and you will not be able to be identified from any of the data published.

**Pseudoanonymised** **data** will be shared with Novo Nordisk. This may include information such as your Participant ID and date of birth. It will not be possible to identify you from the shared data.

Anonymous datasets from the trial may also be made available to other researchers in line with national and international data transparency initiatives.

At the end of the trial, the trial team will be able to provide you with a copy of the results and the publications that arise from the research. The team will be happy to go through these in detail with you. The results of the trial will also be available in the John van Geest Centre for Brain Repair annual newsletter. You will have the option of receiving this newsletter if you wish.

## Who is funding the trial?

The trial is being funded by a research grant from Novo Nordisk, a Danish pharmaceutical company. Novo Nordisk will not be responsible for any part of the trial or involved in any data management or interpretation of the results of the trial.

## Who has reviewed this trial?

This trial has been peer reviewed by representatives of Cambridge University Hospitals NHS Foundation Trust. All research within the NHS is reviewed by an independent group of people called a Research Ethics Committee, to protect your interests. This trial has been reviewed and given favourable opinion by the Oxford A Research Ethics Committee in the UK and reviewed and approved by the Swedish Ethical Review Authority in Sweden. The Medicines and Healthcare Products Regulatory Agency (MHRA) and the Medical Products Agency (MPA) who are responsible for regulating medicines in the UK and Sweden respectively have also reviewed this trial.

## Further information and contact details

If you would like further information or would like to discuss any aspect of participating in this trial, please do feel free to contact:

**Professor Roger Barker (Clinical Lead)**

*For complaints please contact:*

*PALS and Complaints Department*

Box 53, Cambridge University Hospitals NHS Foundation Trust

Hills Road

Cambridge

CB2 0QQ

Email: cuh.complaints@nhs.net

Tel: 01223 216756

# *In the event of an emergency please contact*:

The 24-hour Addenbrooke’s switchboard on 01223 245151 and ask for a member of the STEM-PD trial team.

If for any reason you attend Accident & Emergency, make sure you show your participant alert card.

## INFORMED CONSENT FORM

**Trial Title:** **STEM-PD** Trial: A clinical trial to evaluate the safety of transplantation of stem cell-derived dopamine cells, that have not been tested in humans before, into the brain of individuals with Parkinson's disease

**(Formally registered as:** STEM-PD trial: A multicentre, single arm, first in human, dose-escalation trial, investigating the safety and tolerability of intraputamenal transplantation of human embryonic stem cell derived dopaminergic cells for Parkinson’s disease (**STEM-PD**))

**Clinical Lead:** Professor Roger Barker **Participant Number:** _________

| If you agree with each sentence below, please initial the box | | **INITIALS** |
| --- | --- | --- |
| 1 | I have read and understood the participant information sheet version ##, dated #### for the above trial and I confirm that the trial procedures and information have been explained to me. I have had the opportunity to ask questions and I am satisfied with the answers and explanations provided. |  |
| 2 | I understand this is the first time that the **STEM-PD** cells will be used in humans and there may be unanticipated side effects. |  |
| 3 | I understand that my participation in this trial is voluntary and that I am free to withdraw at any time, without giving a reason and without my medical care or legal rights being affected. |  |
| 4 | I understand that personal information about me will be collected and used in accordance with this information sheet. This information will be kept in the strictest confidence and none of my personal identifiable information will be published. |  |
| 5 | I understand that sections of my medical notes or information related directly to my participation in this trial may be looked at by responsible individuals from the Sponsor, regulatory authorities and research personnel where it is relevant to my taking part in research and that they will keep my personal information confidential. I give permission for these individuals to have access to my records. |  |
| 6 | I understand that my GP will be informed of my participation in this trial and sent details of the STEM-PD trial. |  |
| 7 | I understand that my personal data will be transferred to Lund in Sweden in order to arrange surgery/trial procedures. I understand that any personal data will be sent using appropriate secure transfer methods. |  |
| 8 | I understand that **pseudoanonymised** and/or anonymised data will be shared with external collaborators including the Royal Free Hospital, Novo Nordisk, Lund University, and Region Skåne – Skåne University Hospital for the purposes of this trial. |  |
| 9 | I have read and understood the compensation arrangements for this trial as specified in the Participant Information Sheet. |  |
| 10 | I understand that the doctors in charge of this trial may close the trial or stop my participation in it at any time without my consent. |  |
| 11 | I have read and understood my responsibilities for the trial including using appropriate contraception as listed in section 7. |  |
| 12 | I agree to video recording of certain trial assessments as detailed in section 8 and acknowledge that my face cannot be obscured. |  |
| 13 | I understand that de-identified information collected about me may be used to support other research in the future, including research conducted by both commercial and non-commercial organisations in the UK and abroad. |  |

| **OPTIONAL** | | **YES** | **NO** |
| --- | --- | --- | --- |
| 14 | I agree to undergo the **optional** lumbar punctures (and associated blood testing) as part of this trial. |  |  |
| 15 | I agree to provide the additional **optional** research blood samples as part of this trial. |  |  |
| 16 | I understand that my samples may be retained for future research with appropriate regulatory and ethical approval. Samples may be sent to organisations, including commercial organisations, in the UK and abroad. |  |  |
| 17 | I agree to allow the trial investigators to contact me in the future with respect to further follow-up for this trial or related ethically approved studies. |  |  |
| 18 | I agree for my research samples to be used for future ethically approved research studies. |  |  |
| 19 | I agree to be kept informed of the progress of the trial |  |  |

**I agree to participate in this trial:**

Name of participant Signature Date

Name of person taking consent Signature Date

Time of Consent (24hr clock) _______:_______

1 copy for the participant, 1 copy for the trial team, 1 copy to be retained in the hospital notes.

**Information till forskningspersoner & informerat samtycke**

En klinisk studie för att utvärdera säkerheten av transplantation av stamcellsderiverade dopaminceller till hjärnan hos individer med Parkinsons sjukdom

Vi frågar dig om du vill delta i en forskningsstudie. I det här dokumentet får du information om studien och vad det innebär att delta. Här finns också information om vad som förväntas av dig och om risker och eventuella fördelar med studien. Diskutera gärna studien med dina närstående och ställ frågor till din läkare om allt du undrar över innan du bestämmer om du vill delta.

Vad är syftet med studien och varför blir jag tillfrågad om medverkan?

Syftet med studien är att undersöka säkerheten av transplantation av en ny cellprodukt till hjärnan vid Parkinsons sjukdom.

Vid Parkinsons sjukdom dör nervceller i hjärnan som producerar signalsubstansen dopamin.

Läkemedel som nu används för att behandla Parkinsons sjukdom ersätter dopamin.

Läkemedelsbehandling är mycket effektiv under de första åren av Parkinsons sjukdom. Med tiden minskar dock effekten och besvärliga svängningar mellan bra och dålig rörlighet uppstår. Det finns så kallade avancerade behandlingar (pumpbehandling med Duodopa, Lecigon eller apomorfin och implantation av djupa hjärnelektroder (DBS)). Dessa terapier kan ge en god effekt på symptomen, i alla fall under en viss tid.

Ingen av de nuvarande behandlingarna kan ersätta de förlorade dopamincellerna och påverka sjukdomens förlopp. Vi forskar sedan många år på om förlorade dopaminceller kan ersättas genom experimentell transplantation av nya dopaminceller till hjärnan.

Tidigare experimentella behandlingsstudier i Sverige och utomlands med transplantation av dopaminceller från donerad fostervävnad har visat blandade resultat. Vissa transplanterade patienter har upplevt förbättring av rörligheten och minskade svängningar, mindre stelhet och förbättrad gång. Några av dessa patienter har kunnat minska eller sluta helt med sina anti-Parkinson läkemedel under flera år, medan andra patienter inte har upplevt någon påtaglig förbättring. En del av de transplanterade patienterna i tidigare studier med fostervävnad utvecklade biverkningar i form av bestående besvärande överrörlighet som i vissa fall krävde en neurokirurgisk åtgärd med DBS.

Fostervävnad finns dock tillgänglig för transplantation bara i små mängder vilket gör att endast få patienter kan opereras. Kvaliteten på cellerna och därmed effekten varierar alltför mycket.

Detta har lett till arbete med att i stället försöka framställa dopaminceller från mänskliga stamceller. Sådana celler kan standardiseras, frysas in, kvalitetstestas innan användning och tillverkas i stora mängder, så att de kan bli tillgängliga för många patienter.

Stamceller är celler som kan utvecklas till många olika celltyper, inklusive hjärnceller. Forskare vid Lunds universitet har utvecklat en stamcellsprodukt som kallas för STEM-PD. *Mer om produkten kan du läsa i nästa avsnitt (STEM-PD produkten).*

I denna studie undersöker vi primärt om STEM-PD är säker att transplantera till personer med Parkinsons sjukdom. Vi undersöker två olika doser av celler. Vidare undersöker vi om cellerna överlever och mognar till dopaminceller. Parkinsonsymtomen mäts för att avgöra om de förändras efter transplantationen.

Det är första gången STEM-PD produkten ges till människor.

Vi ber dig ta ställning till om du vill delta i studien eftersom du har Parkinsons sjukdom och har deltagit i TransEuro observationsstudie.

# STEM-PD produkten

STEM-PD har utvecklats från en mänsklig embryonal stamcellslinje som kommer från ett donerat överblivet befruktade ägg från in vitro-fertilisering.

Forskare vid Lunds universitet har utvecklat ett specifikt protokoll som gör att stamcellernas utveckling styrs till ett förstadium av dopaminproducerande nervceller. Cellprodukten kallas i detta stadium för STEM-PD.

En stor mängd celler har framställts och förvaras djupfrysta. Samma STEM-PD celler som kommer att transplanteras i denna studie har testats i djurförsök. I djurstudier har cellerna efter transplantation till hjärnan mognat till dopaminceller. Djurstudierna har visat att STEM-PD produkten inte leder till tumör i djur och att cellerna kan minska symtom som beror på dopaminbrist.

När och i vilken omfattning STEM-PD cellerna mognar och utvecklas i den mänskliga hjärnan är fortfarande okänt och kommer att undersökas i denna studie. Säkerhet av cellerna i människor är också okänt. *Se sida 7 för möjliga risker med STEM-PD.*

# Organisation och godkännande

Studien genomförs vid Skånes Universitetssjukhus i Lund, VE (verksamhetsenhet) Neurologi i samarbete med VE neurokirurgi och andra enheter inom sjukhuset. Studien är ett gemensamt projekt mellan Skånes Universitetssjukhus (Region Skåne), Lunds universitet, Cambridge University Hospitals NHS Foundation Trust (CUH) & Cambridge University i Storbritannien. Studien kommer även att genomföras i Cambridge, Storbritannien.

Forskningshuvudman för studien är Region Skåne. Med forskningshuvudman menas den organisation som är ansvarig för studien i Lund. Region Skåne är även sponsor för studien och därmed ansvarig för upplägg och genomförandet av studien och för datahantering och rapportering.

Studien finansieras till stor del av ett forskningsbidrag från det danska läkemedelsföretaget Novo Nordisk till Lunds universitet. Novo Nordisk ansvarar inte för studien och har ingen del i datahantering eller tolkning av resultaten.

Studien är granskad och godkänd av Etikprövningsmyndigheten och Läkemedelsverket.

Hur går studien till?

Du behöver ta ställning till om du vill delta i studien, och bekräfta det genom att skriva under ”Informerat samtycke”.

Om du bestämmer att du vill delta, undersöker vi dig först för att se om du uppfyller alla kriterier för deltagandet (screeningbesök).

Om screening visar att du kan delta, följer en rad undersökningar för att registrera dina symtom före transplantationen, som utgör baseline-symtom som effekterna senare jämförs med.

Därefter genomgår du en hjärnoperation med transplantation av STEM-PD celler till hjärnan.

Efter operationen undersöks du regelbundet under 3 år. Det är ett omfattande åtagandande för dig med 33 planerade sjukhusbesök i Lund.

I studien kommer följande typer av mättningar göras:

- Blodprov och läkarundersökning
- Rörelsetester i så kallad OFF och ON. Testerna kommer att videofilmas
- Kognitiva tester (enstaka tester kan videofilmas)
- Enkäter om stämningsläge, andra icke-motoriska symtom och livskvalitet
- PKG®-aktivitetsklocka och Parkinsons dagbok
- Magnetresonans kameraundersökningar
- PET-kameraundersökningar (isotop undersökning med positron emissionstomografikamera)
- CT-angio kameraundersökning

*På sida 17-21 beskrivs alla besök och vad som kommer att genomföras i mer detalj och en översikt av alla besök finns på sista sidan.*

Åtta patienter planeras att delta i studien; cirka 4 deltagare från Sverige och 4 från Storbritannien. Två olika doser av celler planeras. De första 4 deltagarna får dos 1. Efter en utvärdering bestäms om de kommande 4 deltagarna får samma dos eller en högre dos. En oberoende grupp av experter kommer att granska data från dos 1 för att bedöma säkerheten av STEM-PD produkten och transplantationen.

# Informerat samtycke

Du får denna skriftliga information om studien och vi kommer även att ge dig muntlig information.

Om du kan tänka dig att vara med i studien kallas du till ett besök till neurologimottagningen i Lund. Du får gärna ta med en anhörig/närstående till besöket. Vid besöket går vi genom studien en gång till och ni har möjlighet att ställa ytterligare frågor.

Efter besöket får du tid på dig att prata om studien med din närstående. Om du bestämmer dig för att delta i studien skriver du under ett ”Informerat samtycke”.

**Screening och baseline besök**

*Se sida 17 för syftet och vilka undersökningar som kommer att utföras*

# Förberedelse inför operation

Du kallas för ett inskrivningsbesök då blodprov tas och du kommer att träffa narkosläkare.

Dagen efter eller senare utförs även en magnetkamera-undersökning under narkos för en detaljerad planering av operationen. Det kommer också utföras en CT angiografi för att synliggöra blodkärl i operationsområdet.

# Inläggning på sjukhus och operation

Kvällen före operation blir du inlagd på sjukhuset. Vanliga rutiner inför en hjärnoperation genomförs liksom blodprov tas och du får antibiotika för att förebygga infektion. Du börjar även med immunhämmande läkemedel. *Se nästa avsnitt.*

Under operation och vid transplantation av STEM-PD kan även medprövare Håkan Widner vara med. Han är även sponsor-representant, men kommer i så fall vara med som medprövare.

Operationen sker med en så kallad stereotaktisk ram som fästs i skallbenet under lokalbedövning. Om du föredrar kan detta göras under narkos. Du genomgår en magnetkamera-undersökning med ram för att beräkna målpunkter. Efter magnetkamera-undersökning flyttas du till operationsavdelningen varefter du sövs för operationen.

Operationen börjar med ett snitt av ca 5 cm i skalpens hud, på en sida av hjässan. Ett eller två hål om 14 mm borras genom skallbenet. En liten öppning görs i hjärnhinnan och ett 1,2 mm tunt transplantations-instrument förs försiktigt in i hjärnan till området som kallas putamen (ca 10 cm ned i hjärnan). Där deponeras STEM-PD celler i små portioner om 2,5 mikroliter i 4 – 8 punkter i en linje från den nedre delen av putamen till den övre med 1 – 2 mm mellanrum. Antalet punkter (4 eller 8) beror på vilken dosgrupp du blir tilldelad. Instrumentet förs ned 5 gånger i olika delar av varje putamen för att STEM-PD cellerna när de mognar ut skall nå till så stor volym av putamen som möjligt. Hjärnhinnan försluts, benet täcks, underhuden sys och såret i läderhuden sluts med agraffer som ska tas bort 7 – 10 dagar senare. Det hela upprepas sedan på andra sidan av hjärnan. Hela operationen tar ungefär 8 – 10 timmar.

Transplantationsinstrumentet som används är Rehncrona-Legradi instrumentet. Instrumentet har utvecklats av en neurokirurg och medicintekniker i Lund och har använts i alla tidigare studier i Lund då fostervävnad transplanterats till hjärnan. Användning av instrumentet i denna studie har godkänts av Medicinsk Teknik Skåne.

Efter operation kommer du att överföras till en övervakningsavdelning, och om ditt tillstånd är stabilt och bra kommer du att flyttas till en vårdavdelning och vara inlagd 4 – 6 dagar. De första 3 dagarna kommer du att vara i ett isoleringsrum för att skydda dig under fasen med de högsta doserna av de immunhämmande medicinerna.

Under vårdtiden görs kontroller och upprepade blodprov tas framför allt för att justera dosen av den immunhämmande behandlingen. Inom de första 3 dagar efter operationen görs en magnetkameraundersökning av hjärnan för att kontrollera om transplantationen har gett upphov till några negativa reaktioner eller komplikationer.

När du har återhämtat dig, och Parkinsonmediciner och den immunhämmande behandlingen är inställda skrivs du hem.

Innan hemgång får du ett deltagarkort. Du bör alltid ha det här kortet med dig och visa upp det vid kontakt med sjukvården.

Inför varje uppföljningsbesök kommer vi att skicka dig en kallelse och vi kontaktar dig också per telefon som påminnelse om besök eller aktiviteter, exempelvis starta med PKG®-aktivitetsklocka och dagbok, och om du ska sätta ut Parkinson mediciner inför besöket.

# Extra läkemedel - immunhämmande och profylaktiska

Du kommer att behöva ta andra läkemedel som en del i studien. Det är godkända, registrerade läkemedel som inte undersöks i studien.

STEM-PD produkten består av mänskliga celler som ditt immunsystem kan uppfatta som främmande och bilda försvarsreaktioner mot. För att undvika avstötning av de transplanterade cellerna får du en kombination av immunhämmande läkemedel. Du börjar med dem dagen före operation och fortsätter under 12 till 15 månader efter operationen. Det handlar om följande mediciner:

- Prednisolon (kortisonpreparat)
- Takrolimus (om det inte är lämpligt får du ciklosporin i stället)
- Azathioprin

Det är mycket viktigt att du tar dessa mediciner enligt anvisningarna. Regelbundna blodprov tas för att fastställa den korrekta dosen av vissa immunhämmande mediciner och doserna kan behöva justeras.

Immunhämmande medel leder till ökad infektionskänslighet. För att motverka en del av dessa behandlas du i förebyggande syfte med antibiotika varannan dag under perioden när du tar immunhämmande läkemedel. Antibiotika ger skydd mot vissa vanliga typer av infektioner som kan uppträda med behandling av immunhämmande medel.

Långvarig användning av kortikosteroider ökar risken för benskörhet, som innebär att skelettet blir svagare och att man lättare kan få benbrott. För att minska risken för att utveckla detta får du medicinen alendronsyra som du tar en gång i veckan, och ett kalcium / vitamin D-tillskott. Både ska du använda under perioden du använder prednisolon.

Under tiden du använder dessa extra läkemedel ska du inte inta grapefrukt, grapefruktjuice eller koncentrerade citrusfrökapslar. Dessa kan påverka hur takrolimus och ciklosporin verkar och ge upphov till biverkningar. Vissa smärtstillande läkemedel ska användas med försiktighet. Nya mediciner ska du bara använda i nära samråd med studie-teamet.

Under perioden du är inlagd för operation får du ytterligare läkemedel för att förebygga infektioner och hämma immunsystemet. Om du vill veta mer om dem kan du fråga studieläkaren.

# Graviditet och amning

Vissa immunhämmande läkemedel kan skada ett ofött barn eller ammande barn. Du kommer inte att kunna delta i denna studie om du är gravid eller ammar eller planerar att bli gravid under studien. Är du man som deltar i studien måste du se till att ingen kvinna blir gravid under den perioden du använder immunhämmande läkemedlen. Du bör också avstå från att donera spermier under den perioden.

Studieläkare kommer att diskutera effektiva preventivmedel med dig. Prata med din partner om detta.

Blir du eller din partner ändå gravid under studien? Låt studieläkare veta omedelbart. Läkare kommer att diskutera med dig om/hur läkemedel behövs anpassas och vad som ska göras.

# Vad vi förväntar från dig om du deltar i studien

Sammanfattningsvis, betyder deltagandet i studien följande för dig:

- Du genomgår en operation för transplantation av STEM-PD cellerna till hjärnan.
- Du deltar i alla studiebesök under lite mer än 3 år och kommer medicin-fastande när det behövs.
- Du tar alla extra mediciner du får från studieläkaren under 12–15 månader.
- Du fortsätter ta din Parkinson mediciner som tidigare. Behandlingen kommer att anpassas vid behov.
- Det finns läkemedel som kan påverka STEM-PD cellerna eller de mediciner du får i studien. Det är därför viktigt att du rådfrågar studieteamet innan du börjar använda något nytt läkemedel inklusive naturläkemedel, naturmedel, kosttillskott, örtmediciner och vitaminer eller dylikt.
- Du ska berätta för studieteamet om du känner dig dålig eller annorlunda på något sätt. Om du utvecklar större problem eller allvarliga symtom, kontakta studieläkare omedelbart enligt kontaktinformationen på sida 15 i denna information eller på ditt deltagarkort.
- För att kunna delta i studien måste du vara fullständigt vaccinerad mot Covid-19.
- Diskutera följande information med din partner:
  - Du bör inte delta i studien om du planerar att bli gravid eller få barn under studien. Om du skulle kunna bli gravid ska du använda effektiva preventivmedel.
  - Som manlig studiedeltagare ska du se till att ingen kvinna blir gravid under den perioden du använder immunhämmande läkemedlen och du ska avstå från att donera spermier.

# Möjliga följder och risker med att delta i studien

## Cellprodukt, operation och profylaktiska mediciner

### STEM-PD

STEM-PD är framtagen från stamceller som kan utvecklas till många olika celltyper, såsom dopaminceller. Stamceller har också en stor förmåga att föröka sig. Cellerna i STEM-PD har inte längre denna förmåga, men eventuella kvarvarande stamceller kan i teorin ge upphov till någon form av godartad eller elakartad tumör. STEM-PD är testad i djur och där har produkten visat sig vara säker och stabil. Det betyder att cellerna inte spritt sig i kroppen, inte lett till toxicitet och att ingen onormal tillväxt eller tumör har hittats i något av de djurförsök som genomförts med STEMPD-produkten.

STEM-PD produkten används nu för första gången i människor i denna studie. Det betyder att vi inte vet om produkten är säker för människor och för patienter med Parkinsons sjukdom. Vi kan inte utesluta att oväntade problem uppstår när produkten ges till människor. Det är viktigt att du omedelbart berättar för studiepersonalen om eventuella biverkningar eller symtom du utvecklar under studien, oavsett om du tror att de är relaterade till STEM-PD-produkten eller ej.

Det finns en risk att några celler utvecklar sig till en annan typ av nervceller som bildar andra signalämnen såsom serotonin vilket kan leda till utveckling av ofrivilliga rörelser. Detta är ett problem som drabbade några patienter i de tidigare studierna med transplantation av fostervävnad. Vid studier på djur som transplanterats med STEM-PD har inga serotoninbildande celler påvisats. Om du ändå utvecklar svåra ofrivilliga rörelser, kommer det att vara möjligt att behandla dem med etablerade metoder. Dessa kan omfatta läkemedel eller behandling med DBS (djupelektrodstimulering i hjärnan).

### Hjärnoperationen

Varje operation innebär en risk för **komplikationer**. Gemensamt för alla operationer är att det finns risk att en blodpropp bildas i benen under operationen, och som kan om den lossnar, fastna i lungan, en så kallad lungemboli. Detta kan vara mycket allvarligt och dödligt. Det är inte möjligt att ge full blodförtunnande behandling som skydd mot detta, då risken för blödning i hjärnan istället ökar. Under operationen och efteråt ges det vadmassage med maskin som minskar risken och efter operation kommer du att uppmanas att röra dina ben mycket. I Lund har det under 20 års tid med DBS verksamhet förekommit 1 fall med allvarlig lungemboli på ca 1000 operationer.

Stereotaktisk hjärnoperation är ett vanligt förekommande ingrepp, och standardiserade procedurer kommer att tillämpas som minskar riskerna med operation. Det finns stor erfarenhet i Lund av stereotaktisk implantation av fostervävnad i putamen, den hjärnregionen i vilken transplantationen kommer att ske. Risken för komplikationer efter neurokirurgiska operationer mäts kontinuerligt sedan många år och anges som alla komplikationer inom 1 månad från en operation. I Lund vid VE Neurokirurgi förekommer komplikationer för stereotaktiska operationer vid maximalt 1 på 40 operationer, 2,5%. Komplikationerna som rapporterats omfattar allt från sårinfektion till allvarlig komplikation i hjärnan. Risken för allvarligt neurologiskt handikapp efter stereotaktisk operation i hjärnan vid Parkinsons sjukdom med allvarlig symtomgivande blödning har varit mindre än 2 promille (2 av 1069) patienter. Operationen i denna studie planeras med hjälp av magnetkamerabilder samt CT-angio bilder så att risken att skada blodkärl minskas.

Efter en hjärnoperation är det olämpligt att köra bil under en tid. Du förväntas därför att inte köra bil under första tiden efter operation och förrän en ny bedömning gjorts av studieläkaren.

### Immunhämmande medel

De vanligaste riskerna och biverkningarna vid långvarig användning av immunhämmande medel är att:

- Du blir mer känslig för infektioner från din omgivning. Du får ett informationsblad med hygienregler och hur du bäst kan agera gentemot infekterade personer i din omgivning. CoVID-19 infektion som drabbar en ovaccinerad person som behandlas med immunhämmande läkemedel ökar risken för infektionen att få ett allvarligt förlopp. Redan vaccinerade personer som startar med immunhämmande behandling har ett skydd mot ett allvarligt förlopp.

Du måste därför vara fullt vaccinerad enligt gällande föreskrifter för CoVID-19 infektion för att kunna delta i studien. Tidig behandling av infektioner är viktigt, så du kommer att informeras om att kontakta oss vid infektionssymtom.

- Vilande infektioner i kroppen kan aktiveras av immunhämmande behandling. Under screeningen kommer vi att kontrollera riskfaktorer och leta efter potentiella vilande infektioner. Blodprov tas för att bedöma eventuella tidigare exponering för vissa virus som kan finnas vilande i kroppen.
- Tidigt efter operationen kommer höga doser av prednisolon att användas, vilket ökar risken för biverkningar. Möjliga biverkningar av de höga doserna inkluderar mani, psykos, förvirring och andra neurologiska symtom; dessa förekommer endast hos få patienter.
- Immunhämning innebär en något ökad risk för utveckling av vissa tumörer, vanligen i blodet eller huden. Eftersom du använder de immunhämmande medlen under kort tid (upp till 15 månader) är risken liten och du kommer att kontrolleras för detta.
- Vissa immunhämmande mediciner kan ge upphov till ökat blodsocker och typ 2 diabetes. Det kan krävas behandling med mediciner eller låga doser insulin. Vi kommer att kontrollera ditt blodsocker regelbundet under studien. Oftast, men inte alltid, normaliseras blodsockret efter att medicineringen avslutas.
- Det finns även en ökad risk för utveckling av benskörhet, men risken är låg med kort immunhämningsperiod och med de läkemedel som ges som motverkar benskörhet.

*Mer information om de mediciner du ska använda finns i bipacksedlarna som följer med läkemedlen.*

## Provtagning och mätningar

*Studiens besök på sjukhuset utan Parkinson mediciner* är obekväma men medför inga risker. Det finns möjlighet att övernatta på patienthotellet om resan till Lund känns för besvärlig utan parkinsonmediciner.

### Risker med PET-kamera undersökningar

PET/CT-undersökningarna av hjärnan i studien ger en något ökad exponering för joniserande strålning. Stråldosen som en person i studien utsätts för är ca 15 mSv/år och totalt under 3 år 45 mSv. Detta är inklusiv en mindre stråldos för CT-angiografi som görs en gång före operationen.

Denna stråldos kan sättas i relation till den dos som alla människor erhåller dagligen från naturliga strålkällor såsom kosmisk strålning, naturligt förekommande radionuklider i luft, mark och i den egna kroppen samt från byggnadsmaterial. Denna s.k. naturliga bakgrundsstrålning resulterar i en genomsnittlig stråldos till en person som lever i Sverige, på ca 1 mSv/år. Inkluderar man stråldosen som en del personer boende i Sverige får på grund av medicinsk bestrålning och exponering från radon i hus och mark uppgår den årliga medel-stråldosen till ca 3 mSv.

Ökningen av stråldosen på grund av medverkan i denna studie är sålunda måttlig och vi bedömer risken för strålningsframkallad cancer på grund av deltagande i studien som låg. Stråldoserna har godkänts av Etikprövningsmyndigheten.

### Obehag med PET-kamera undersökningar

Du behöver ligga stilla i 75–90 minuter vilket kan upplevas som obehagligt. PET-kamera undersökningen sker i OFF och då ska du inte ta dina Parkinson mediciner 12 till 24 timmar innan besöket. Du kommer att få tydligare Parkinson symtom, med stelhet, skakningar och svårigheter med rörligheten mm. Även andra läkemedel kan sättas ut beroende på din läkares bedömning. Personal kommer att se till att du ligger så bekvämt som möjligt under undersökningen och de finns tillgängliga under hela tiden. Vid vissa undersökningsdagar ska du avstå från nikotin och koffein före undersökningen.

En venkateter (för att injicera spårämnen) kan vara obehaglig, smärtsam och orsaka blåmärke vid insättning. Det är ovanligt med några kvarvarande obehag efter att katetern tagits bort.

### Risker och obehag med CT-angio undersökningen

CT-kameran är en kort tunnel och tiden du ligger i kameran är ungefär 5 minuter. En venkateter används för att injicera kontrastmedel.

### Risker med MR-kamera undersökningar

MR-kameror använder ett mycket kraftigt magnetfält. Du kommer att tillfrågas om du har några metallföremål i kroppen som pacemaker, metallclips, proteser, metallsplitter eller liknande för att du säkert skall kunna genomgå undersökningen. Om ingen MR-kamera undersökning kan genomföras kan du inte delta i studien. Har du tendens till cellskräck kan det vara omöjligt att genomföra undersökningarna. Det är inte möjligt att ge lugnande medicinering under MR-kamera undersökningen, då det påverkar resultaten.

### Obehag i samband med MR-kamera undersökningar

MR-kamera undersökning kan upplevas som obehaglig då den sker i ett mycket trångt utrymme och kameran bullrar mycket under undersökningen. Du får hörselproppar och hörlurar för att dämpa oljudet. Du behöver ligga så stilla som möjligt under undersökningen (20 till 60 minuter beroende på typ av undersökning) för att kvaliteten på bildmaterialet skall bli så bra som möjligt.

### Risker och obehag i samband med ryggvätskeprov – frivilliga extra prov

Provtagningen gör vanligen inte ont. De flesta mår som vanligt efteråt. En del får huvudvärk ett par timmar till 2 dagar efter provtagningen. Besvären kan vara kvar i flera dagar. Risken kan minskas genom att dricka tillräckligt med vatten. Huvudvärken kan lindras om du lägger dig ner. Ligg gärna utan kudde så att huvudet är i höjd med ryggraden.

### Risker och obehag i samband med blodprovstagning

Blodprovstagning kan orsaka obehag och kan ibland leda till lokala blåmärken och infektion på punkteringsstället.

## Möjliga fördelar med att delta i studien

STEM-PD produkten ges nu för första gång till människor, vilket betyder att vi inte vet om cellerna överlever, om de utvecklas till dopaminceller, och om de kommer att ha effekt på din Parkinsons sjukdom. Det finns alltså ingen garanti att du kommer ha nytta av att delta i denna studie.

Resultaten från studien skulle kunna bidra till att utveckla en ny behandling för Parkinsons sjukdom.

Om du utvecklar negativa Parkinsonrelaterade symtom eller otillräckliga effekter har du fortfarande tillgång till alla tillgängliga etablerade behandlingar för Parkinsons sjukdom efter studien och om det behövs, även under studien.

## Alternativa behandlingar för Parkinsons sjukdom

Om du väljer att inte delta i denna studie får du fortsatt tillgång till den vanliga Parkinsonvården och det påverkas inte av ditt ställningstagande. Om dina Parkinsonmediciner inte längre ger optimal effekt kommer din läkare att diskutera alla andra tillgängliga behandlingar med dig.

Vad händer med mina uppgifter?

Ditt deltagande i forskningsstudien registreras i din patientjournal. I studien kommer besöken att journalföras som om det vore ett normalt sjukvårdsbesök. De uppgifter som registreras i journalen är personnummer, namn, adress, kliniska data, laboratoriedata, och även enstaka testresultat och enkätsvar från dig som om du vore en ordinarie patient. STEM-PD cellernas ursprung kommer också att journalföras genom att registrera deras ”batch nummer”. Normala journalsekretessregler gäller.

Parallellt kommer alla resultat av undersökningar i studien att lagras i en databas. Uppgifter såsom ålder, kön, hälsodata (såsom nuvarande och tidigare sjukdomar) hämtas från din sjukvårdsjournal och lagras också i studiedatabasen.

Videoinspelningar av dina rörelsetester sparas på en extern disk som hålls inlåst. De kommer att bedömas av oberoende Parkinsonspecialister.

MR- och PET-kamerabilder av din hjärna kommer att delas med forskare vid Imperial College in

London som genomför MR- och PET-kamera undersökningar på de engelska deltagarna i studien.

All information om dig i studiedatabasen, videofilmer samt MR- och PET-kamera bilder är kodade, dvs. bär inte ditt namn eller födelsedatum, utan en kod. Bara studie-teamet vid neurologi i Lund har tillgång till kodnyckeln.

Ändamålet med denna databas är forskning. Forskning är ur rättslig grund ”allmän intresse”, men dina personuppgifter är sekretesskyddade och ingen obehörig har tillgång till databasen. Dina svar och dina resultat kommer att förvaras och behandlas så att ingen obehörig kan ta del av dem. Vid databearbetning, då studien rapporteras eller publiceras kommer en enskild individ inte att kunna urskiljas.

Databasen finns i Storbritannien hos Norwich Clinical Trial Unit som är kontrakterad av vår samarbetspartner Cambridge sjukhus & universitet. Detta betyder att dina personuppgifter kommer att överföras till ett land utanför EU (s.k. tredjeland). Det finns ett avtal mellan samarbetspartners som också säkerställer ett adekvat skydd av dina uppgifter. Databearbetning och analys kommer att göras av Forum Söder som är en del av Region Skåne och stödjer forskningen inom Skånes universitetssjukhus. Data kommer att delas med de samarbetspartnerna i studien, Lund Universitet och Cambridge sjukhus & universitet.

Resultat av undersökningar kommer även att delas med den oberoende grupp av experter som ska bedöma säkerheten av STEM-PD produkten och transplantationen. Experterna finns inom EU och Storbritannien.

Kodade data kommer att delas med Novo Nordisk som kan använda data för vetenskaplig forskning och för att utveckla STEM-PD till ett läkemedel. Kodade säkerhetsdata kan komma att delas med tillverkaren av STEM-PD-produkten, Royal Free Hospital i Storbritannien, om det är nödvändigt för att spåra eventuella oväntade reaktioner.

# Dina rättigheter

Enligt Dataskyddsförordningen, GDPR, (EU 2016/679) har du rätt att ansöka om information om vilka personuppgifter som behandlas, få rättelse av personuppgifter samt rätt att begära att data raderas. När det gäller forskning är det inte alltid möjligt att få redan insamlade data raderad. Region Skåne är ansvarig för behandlingen av personuppgifterna. Rätten till radering och till begränsning av behandling av personuppgifter gäller dock inte när uppgifterna är nödvändiga för den aktuella forskningen. Dataskyddsombudet är den person som ansvarar för att dina personuppgifter behandlas på ett lagligt och korrekt sätt. Vid behov kan dataskyddsombudet hjälpa dig att få information om vad som registrerats och få eventuella rättelser genomförda. Kontakta i första hand ansvariga för studien enligt kontaktuppgifter nedan. Dataskyddsombudet för Region Skåne går att nå på: Region Skåne, Dataskyddsombudet, 291 89 Kristianstad. Telefon: 044-309 30 00, e-post: region@skane.se.

Om du är missnöjd med hur dina personuppgifter behandlas har du rätt att framföra klagomål till Integritetsskyddsmyndigheten, som är tillsynsmyndighet.

Dina personuppgifter kommer endast att användas för de ändamål som angivits ovan. De kan endast komma att behandlas för andra syften om du lämnat ett nytt samtycke och/eller om Etikprövningsmyndigheten utfärdat ett nytt godkännande.

# Kvalitetskontroll och arkivering

För att säkra kvaliteten och kontrollera att studien blivit rätt genomförd kommer en kvalitetsgranskare utsedd av sponsorn (Monitor) att jämföra insamlad studiedata med uppgifter i din patientjournal. En myndighetsperson kan också komma granska insamlade data och jämföra med din journal. Kvalitetsgranskaren måste underteckna en sekretessförbindelse för att få tillgång till din medicinska journal. Genom att du skriver under samtycket ger du din tillåtelse till denna insyn i din patientjournal. Studiedata sparas minst 30 år efter att studien är avslutad.

Vad händer med mina prov?

De blodprov, och en del av det frivilliga ryggvätskeprovet som tas i studien skickas direkt för analys i sjukhuset enligt vårdrutiner och kommer förstöras efteråt.

Du kan välja att lämna extra blodprov och/eller ryggvätskeprov. Om proven inte analyseras direkt, kommer de att förvaras i en biobank i enlighet med Biobankslagen (SFS 2023:38) som reglerar på vilket sätt prov får sparas och användas. Biobankens namn är Region Skånes Biobank och den finns i Lund. Huvudman (ansvarig) för biobanken är Region Skåne. Samtliga ovan nämnda prov kommer att vara kodade, vilket innebär att de inte kan kopplas direkt till dig som person. Kodnyckeln ansvarar studieansvarig läkare för, och den förvaras oåtkomlig för obehöriga. Dina prov får enbart användas på det sätt som du har gett samtycke till.

För alla framtida, ännu ej specificerade, forskningsstudier med redan insamlade prov/material krävs en ny etisk prövning och Etikprövningsmyndigheten beslutar då om du behöver kontaktas igen med en ny förfrågan om samtycke.

Du har rätt att utan närmare förklaring begära att dina sparade prov ska förstöras. Om du samtycker till att prov sparas har du rätt att senare ta tillbaka (ångra) det samtycket. Dina prov kommer i så fall att kastas eller avidentifieras. Om du vill ångra ett samtycke ska du kontakta ansvariga för studien enligt kontaktuppgifter nedan.

Prov som sparas i biobanken kan fysiskt komma att sparas hos vår samarbetspartner, Cambridge universitetet i Storbritannien. De får enbart kodade prov och inte personuppgifter som skulle kunna identifiera dig. När studien avslutats kommer samtliga prov att destrueras.

Prov kan också komma att sändas till laboratorium i Sverige eller ett land inom eller utanför EU för analys. Laboratoriet som kommer att analysera proven får bara kodade prov och inte personuppgifter som kan identifiera dig. Dina prov kommer att destrueras eller återlämnas efter analys.

Hur får jag information om resultatet av studien?

Efter studien har avslutats och alla testresultat har analyserats kan du få information om dem muntligt eller skriftligt genom att kontakta studiepersonalen.

Resultaten kommer att publiceras i vetenskapliga tidskrifter och presenteras i samband med nationella och internationella vetenskapliga möten. Enbart statistik på gruppnivå kommer att presenteras och ingen enskild person kommer att kunna identifieras. Information om kliniska prövningar kan hittas på www.clinicaltrials.gov: [https://clinicaltrials.gov/ct2/show/study/NCT05635409.](https://clinicaltrials.gov/ct2/show/study/NCT05635409)

# Försäkring och ersättning

Liksom inom sjukvården i övrigt omfattas du av Patientskadeförsäkringen och Läkemedelsförsäkringen.

Om du tror att du har skadats genom att vara med i den här studien ska du kontakta studieläkaren. Om du drabbas av en negativ händelse till följd av deltagande i den studien kommer du att få den extra hälso- och sjukvård du behöver för den skada du fick.

Deltagandet i studien är utan kostnad för dig. Du får ersättning för dina resekostnader i samband med studiebesök. Du får också ersättning för förlorad arbetsinkomst inklusive restid upp till motsvarande din sjukpenningsgrundande inkomst. Du sjukskrivs på vanligt sätt under operation och tiden efter innan du kan återgå i arbete.

# Deltagandet är frivilligt

Du avgör själv om du vill delta i denna studie. Du kan när som avbryta ditt studiedeltagande utan att uppge någon anledning. Det kommer i så fall inte heller att påverka din fortsatta vård eller behandling av Parkinsons sjukdom. Du kommer att tillfrågas att fortsätta att delta i TransEuroobservationsstudien precis som tidigare.

Om du väljer att avbryta ditt deltagande kommer redan insamlade data och prov fortsatt att användas i projektet, men inga ytterligare uppgifter och prov kommer att samlas in.

Om du vill avbryta ditt deltagande ska du kontakta den ansvariga för studien (se nästa sida).

Om du avbryter deltagandet inom 15 månader efter transplantationen rekommenderar vi dig att fortsätta med de immunhämmande medicinerna och hälsokontroller kopplade till dem.

Om du väljer att lämna studien efter att du har avslutat immunhämmande behandling, kommer vi fortsättningsvis att be dig och din behandlande läkare att informera oss om viktigare händelser i ditt hälsotillstånd, då det är viktigt att kunna samla in alla uppgifter som kan röra säkerhetsinformation om STEM-PD produkten.

När studien är avslutad kommer vi att fråga alla deltagare om de vill delta i en uppföljningsstudie för att kunna undersöka långtidseffekterna av STEM-PD.

När du har avslutat studien efter 3 år kommer du att få en ny forskningspersoninformation och vi kommer då att fråga dig om du vill delta. Deltagandet i uppföljningsstudien är frivilligt.

# Avslutningsvis

Denna forskningspersoninformation innehåller mycket information och det handlar om ett viktigt beslut för dig att delta eller inte delta. Ta god tid på dig att läsa informationen och prata med dina närstående. Ställ också gärna frågor till studieteamet om något är oklart. Du hittar kontaktinformation nedan.

# Ansvariga för studien

**VE neurologi, Skånes universitetssjukhus**  Sök i första hand:

[Namn] (forskningssjuksköterska), [e-postadress], 046 - 17 56 40

[Namn] (forskningssjuksköterska), [e-postadress], [telefon]

Gesine Paul-Visse (neurolog, studieansvarig) 046 - 17 77 66

Håkan Widner (neurolog, sponsor-representant) 046 - 17 14 25

**VE neurokirurgi, Skånes universitetssjukhus**

Hjálmar Bjartmarz (neurokirurg) 046 - 17 13 23

**Besöksadress**

Neurologimottagning Lund, Entrégatan 7, hisshall B, plan 9

Forskningshuvudman:

Region Skåne, Regionstyrelsen, 291 89 Kristianstad

växel: 044-309 30 00

# Samtycke till att delta i STEM-PD studien

Jag har fått muntlig och skriftlig information om studien. Jag har haft möjlighet att ställa frågor och har fått svar på alla mina frågor. Jag har fått tillräckligt med tid att tänka över mitt beslut. Jag vet att deltagande är helt frivilligt. Jag vet också att jag kan när som helst och utan närmare förklaring avbryta mitt deltagande och att det inte påverkar mitt fortsatta omhändertagande.

- Jag samtycker till att delta i studien ”**En klinisk studie för att utvärdera säkerheten av transplantation av stamcells-deriverade dopamin celler till hjärnan hos individer med Parkinsons sjukdom**”
- Jag samtycker till att sponsorn och auktoriserade representanter, Läkemedelsverket och utländska tillsynsmyndigheter får ta del av de för studien relevanta delarna av min patientjournal som beskrivs i denna forskningspersonsinformation.
- Jag har tagit del av informationen om hur mina personuppgifter kommer att hanteras och hur insamlad data om mig förvaras, hanteras och delas med andra parter.
- Jag samtycker till att mina prover sparas i en biobank och skickas till olika laboratorier på det sätt som beskrivs i forskningspersonsinformationen – gäller bara om du samtycker till frivilliga extra prov, se nedan

## Frivilliga extra prov för forskning

Jag samtycker till extra blodprov vid 4 tillfälle som beskrivs i denna information

□ JA □ NEJ

Jag samtycker till ryggvätskeprov vid 3 tillfälle som beskrivs i denna information

□ JA □ NEJ

___________________________ ______________________ ___________

Underskrift (forskningsperson) Namnförtydligande Datum

**Klinikens underskrift**

Jag har informerat om studien och forskningspersonen har fått svar på sina frågor angående studien

___________________________ ______________________ ___________

Underskrift (studieläkare) Namnförtydligande Datum

*Forskningspersonen får en kopia av information och ifyllt och signerat samtycke*

## Bilaga beskrivning av alla besök med händelser

I denna bilaga beskrivs alla besök och vad som kommer att genomföras vid varje besök*.*

*En översikt av alla besök med mätningar hittar du på sista sidan.*

**Screening-besök** (ungefär 3 timmar)

Efter det informerade samtycket görs undersökningar för att se om du uppfyller kriterierna för att ingå i studien. Vi kontrollerar bland annat att du inte har utvecklat andra symtom eller sjukdomar som bedöms vara olämpliga för transplantation eller behandling med immunhämmande mediciner.

Du ombeds att komma i så kallat OFF: du ska inte ta dina Parkinson mediciner 12 till 24 timmar innan besöket. Du ska äta en lågproteininnehållande frukost. *Specifika instruktioner får du i kallelse till besöket.*

Under besöket kommer följande att genomföras:

- Genomgång av din sjukdomshistoria, allmänna hälsotillstånd och Parkinsons sjukdom
- Allmän hälsoundersökning
- Blodprov tas. Blod testas bland annat för sjukdomar som hepatit och HIV
- För kvinnor i fertil ålder kommer ett graviditetstest i blod att utföras
- Rörelsetester i OFF och ON
- Kognitiva tester och ett frågeformulär om ditt stämningsläge

Du kommer även kallas till en magnetkamera-undersökning och en PET-kamera-undersökning av hjärnan i separata besök. För PET-kamera undersökningen behöver du komma i OFF. Även andra typer av läkemedel kan pausas tillfälligt inför undersökningen. På undersökningsdagen ska du avstå från nikotin och koffein före undersökningen. *Specifika instruktioner får du i kallelse till besöket.*

Om du uppfyller kriterierna för studien kommer du att kallas till ett baseline besök. Vid besöket mäts symtom och medicineffekter före transplantationen. Dessa tester är lika dem du har genomfört i TransEuro observationsstudien.

**Baseline besök** (ungefär 6 timmar)

Du ombeds att komma i så kallat OFF efter en lågproteinhaltig frukost, liksom vid screeningsbesöket. Innan besöket ska du bära en Parkinsons KinetiGraf (PKG^®^-klocka) i 7 dagar på rad, som registrerar dina rörelser. Du ska även fylla i en Parkinsondagbok under 3 dagar på rad.

Under besöket kommer följande att genomföras: - Allmän hälsoundersökning

- EKG: mätning av hjärtats elektriska aktivitet
- Rörelsetester i OFF och ON. En del av mätningar genomförs upprepade gånger efter du har fått en dos av löslig levodopa (Madopark Quick) av oss.
- Kognitiva tester och frågeformulär om ditt stämningsläge och livskvalitet.
- För kvinnor i fertil ålder kommer ett graviditetstest i blod att utföras

Frivilliga extra prov kan samlas in för forskning.

o Blodprov för mätning av en eventuell immunreaktion på de transplanterade cellerna och för analys av markörer för Parkinsons sjukdom, samt DNA analys (för att bestämma ev. genetisk typ av Parkinsons sjukdom, om du inte redan har analyserats för detta). o Ett ryggvätskeprov för mätning av en eventuell immunreaktion på de transplanterade cellerna och för analys av markörer för Parkinsons sjukdom . *För mer information om provtagning se i slutet av denna bilaga (sida 20).*

Under besöket (eller under planeringsbesök) kommer du även att träffa neurokirurgen. Han förklarar hur operationen går till och du kan ställa frågor.

Du kommer även att kallas för en Magnetkamera-undersökning under narkos och en CTundersökning.

Vidare kommer du kallas till en PET-kamera undersökning av hjärnan i ett separat besök. Du behöver komma i OFF och även andra läkemedel kan utsättas beroende på din läkares bedömning. Du ska avstå från nikotin och koffein på undersökningsdagen. I direkt anslutning till PET-kamera undersökningen görs en magnetkamera-undersökning.

*Specifika instruktioner till de olika kameraundersökningar får du i kallelse till besöket.*

### Besök 7, 14, 21, 28 och 42 dagar efter operation (ungefär 1 timme – dag 28 ungefär 2 timmar)

Sju dagar efter operation är du möjligtvis fortfarande inlagd. Om inte, kommer du till sjukhuset hemifrån. Vid detta besök kommer sannolikt agrafferna i operationssåret att tas bort.

Du ska alltid ta med dig alla medicinförpackningar du fått från oss, även de tomma då all medicineringen behöver bokföras.

Vid varje besök kommer följande att genomföras:

- Vi frågar dig hur du mår och hur du har känt dig sedan ditt senaste besök
- Allmän hälsoundersökning
- Blodprov tas

Vid besöket **28 dagar** efter operation kommer även följande att genomföras:

- EKG
- Magnetkamera-undersökning
- Frågeformulär om ditt stämningsläge
- Endast för kvinnor i fertil ålder kommer ett graviditetstest i blod att utföras.

**Besök 2, 4 och 5 månader efter operation** (1-2 timmar) *För besöket 3 månader efter operation se nedan.*

Du ska ta med dig alla medicinförpackningar du fått från oss (även tomma).

Vid varje besök kommer följande att genomföras:

Vi frågar dig hur du mår och hur du har känt dig sedan ditt senaste besök

- Allmän hälsoundersökning
- Blodprov tas
- Vid **2 månaders** besöket görs även en magnetkamera-undersökning innan besöket

### Besök 3, 9, 18 och 30 månader efter operation (ungefär 3 timmar)

Innan besöket ska du bära en PKG®-klocka i 7 dagar på rad och fylla i en Parkinson dagbok under 3 dagar på rad.

Bara vid **3-månadersbesök**: ett frågeformulär om ditt stämningsläge

Bara vid **3 och 9-månadersbesök:**

- En magnetkameraundersökning kommer att göras i samband med besöket - Du ska ta med dig alla medicinförpackningar du fått från oss även de tomma

Vid **alla besök** kommer följande att genomföras:

- Vi frågar dig hur du mår och hur du har känt dig sedan ditt senaste besök
- Allmän hälsoundersökning
- Blodprov tas
- Rörelse tester
- Vid **18 månaders besök**: ryggvätskeprov tas och extra blodprov om du tidigare har samtyckt till detta och instämmer till att nya prov tas
- För kvinnor i fertil ålder: ett graviditetstest i blod utföras (inte vid 30 månaders besök).

### Besök 6, 12, 24 och 36 månader efter operation (ungefär 6 timmar)

Du ombeds att komma i så kallat OFF och efter en lågproteinhaltig frukost, liksom vid screeningsbesöket. Innan besöket ska du bära en PKG®-klocka i 7 dagar på rad och fylla i en Parkinson dagbok under 3 dagar på rad.

Vid **6 och 12-månadersbesök** ska du ta med dig alla medicinförpackningar du fått från oss även de tomma.

Vid besöket kommer följande att genomföras:

- Vi frågar dig hur du mår och hur du har känt dig sedan ditt senaste besök
- Allmän hälsoundersökning
- Blodprov tas
- Rörelse tester i OFF och ON. En del av mätningar genomförs upprepade gånger efter du har fått en dos av löslig levodopa (Madopark Quick) av oss.
- Kognitiva tester och frågeformulär om ditt stämningsläge och livskvalitet (livskvalitet görs inte vid 6 månaders besök).
- Vid **6 månaders besök**: ryggvätskeprov tas och extra blodprov om du tidigare har samtyckt till detta och instämmer till att nya prov tas

Vid **6 & 12-månaders besök**: Endast för kvinnor i fertil ålder kommer ett graviditetstest i blod att utföras.

Separata besök kommer att bokas för PET-kamera undersökningar; en undersökning vid 6 månader och två undersökningsdagar vid 12, 24 och 36 månader. Magnetkamera-undersökning genomförs i direkt anslutning till PET-kamera undersökningar.

Vid **36 månaders besök** kommer även följande att genomföras:

- EKG
- Extra kognitiva tester som även genomfördes vid baseline besöket
- Extra blodprov tas om du tidigare har samtyckt till detta och instämmer till att ett nytt prov tas - Du kommer bli tillfrågad att delta i en långtidsuppföljning i en separat studie.

### Besök 15 månader efter operation (ungefär 1 timme)

När du har avslutat behandlingen med de immunhämmande läkemedel ombeds du att komma för ett kort besök där följande kommer att genomföras:

- Vi frågar dig hur du mår och hur du har känt dig sedan ditt senaste besök
- Vi mäter kroppsvikt och blodtryck

Du ska ta med dig alla medicinförpackningar du fått från oss även de tomma.

### Hur går specifika undersökningar till

#### Ryggvätskeprov

Ryggvätska är den vätska som omger din ryggmärg och hjärna. Provtagning av ryggvätska är frivilligt. Provet tas från nedre ländryggen med hjälp av en tunn nål. Provtagningen görs med lokalbedövning.

#### Magnetresonanskameraundersökning av hjärnan

Magnetresonans kameraundersökning (MR) ger en noggrann kartläggning av hjärnans utseende. Undersökning genomförs vid bild och funktionscentrum i Lund. Den tar mellan 20 och 60 minuter, beroende på syftet med undersökningen. I studien används MR-kamera för att bedöma om du kan bli opererad (screeningsbesök), för att planera operation (för denna undersökning kommer du att sövas), precis innan operation med stereotaktisk ram och för att undersöka eventuella komplikationer som följd av operation eller av STEM-PD produkten. Vidare görs en utökad MRundersökning för att få mer information om en funktionell förbättring skett till följd av transplantatet. Denna undersökning genomförs i anslutning till en PET-kameraundersökning.

#### CT-angio-undersökning av hjärnan

Computer tomografi (CT)-angio eller även kallad datortomografi-angio av hjärnan är en bildteknik som genomförs med hjälp av ett kontrastämne som injiceras i blodet. Resultat visas som 3D-bilder

på en dator och synliggör blodkärlen i din hjärna. Neurokirurgen använder bilderna vid planering av operationen.

#### PET-kameraundersökning (Positron Emission Tomografi)

PET-kameraundersökning är en bildteknik som genomförs med hjälp av ett radioaktivt spårämne. Genom att injicera en liten mängd av ett radioaktivt märkt spårämne via en venkateter i din arm når spårämnet din hjärna. PET kameran mäter sedan fördelningen av ämnet i din hjärna. Mätningarna visas som 3D-färgbilder på en dator och synliggör förmågan av hjärnan att lagra och transportera dopamin. I denna studie används spårämnen ^18^F-DOPA och ^18^F-FE-PE2I.

Undersökningarna sker med ett spårämne i taget. På morgonen får du äta en lågproteinhaltig frukost. På undersökningsdagen ska du avstå från nikotin och koffein före undersökningen. Före undersökningar behöver du pausa dina Parkinsonmediciner (24 timmar för långtidsverkande mediciner och 12 timmar för korttidsverkande). Även andra läkemedel kan pausas. PET-kameraundersökningen tar 75–90 minuter (beroende på spårämnet) och under tiden ska du ligga så stilla som möjligt.

De radioaktiva spårämnena går ut via urinen och därför uppmanas du att dricka rikligt efter undersökningen och tömma blåsan ofta
