# Supplementary material for: STEM-PD trial protocol: a multi-centre, single-arm, first-in-human, dose-escalation trial, investigating the safety and tolerability of intraputamenal transplantation of human embryonic stem cell-derived dopaminergic cells for Parkinson’s disease
Source: BMJ Open. 2025 Dec 30;15(12):e107597. doi: 10.1136/bmjopen-2025-107597 (PMC12766818; doi:10.1136/bmjopen-2025-107597)
Supplement: Supplementary file 2 [file bmjopen-2025-107597.reviewer_comments.pdf]

PEER REVIEW HISTORY

BMJ Open publishes all reviews undertaken for accepted manuscripts. Reviewers are asked to complete a checklist review form and are provided with free text boxes to elaborate on their assessment. These free text comments are reproduced below.

ARTICLE DETAILS

Title (Provisional)

STEM-PD trial protocol: a multi-centre, single arm, first-in-human, dose-escalation trial, investigating the safety and tolerability of intraputamenal transplantation of human embryonic stem cell derived dopaminergic cells for Parkinson's disease

Authors

Paul, G; Bjartmarz, H; Björklund, Anders; Cutting, Emma; Evans, A; Harry, B; Hansson , Oskar; Kayhanian, Saeed; Kirkeby, A; Lao-Kim, N; Lindvall, Olle; Nelander, J; Piccini, Paola; Smith, R; Ullén, S; Van Vliet[1], T; Widner, H; Parmar, M; Barker, Roger A

VERSION 1 - REVIEW

**Reviewer**	**1**
**Name**	**Alterman, Ron L**
**Affiliation**	**Beth Israel Deaconess Medical Center, Neurosurgery**
**Date**	**07-Aug-2025**
**COI**	**None**

The authors present the protocol for a planned Phase I, open-label safety and feasibility study of allogeneic stem cell transplantation in patients with moderately advanced Parkinson's disease (PD). Patients will be recruited from two centers (Cambridge, UK and Lund, Sweden) but will undergo surgery only at Lund due to the lack of a CE mark for the transplantation device. Eight patients with moderately advanced PD will be recruited and divided into two cohorts of four patients each. The first cohort will receive 3.54 x 106 cells in each Putamen and the second cohort will receive either or 7.08 x 106 or 3.54 x 106 cells. The decision regarding whether to proceed with the dose escalation will be made after the first cohort has been followed for 6 months. Following transplantation patients will be treated with immunosuppressive medications for 12 moths. Standard inclusion and exclusion criteria are proposed as are standard primary and secondary outcome measures. Ethics boards at both institutions have approved the protocol.

This protocol description meets the criteria for publication in BMJ Open. I do have the following criticisms/comments:

1. I'm not sure how the authors can claim that this is a first in human trial given that Tabar, et al have already published a first in human trial of allogeneic embryonic stem-cell derived dopaminergic neurons (Nature; May 2025). How is this different?

2. A more detailed description of the implant surgery is warranted, particularly how many trajectories per side and how many deposits per trajectory.

3. The authors' claim that their choice of study cohort was based on prior research, which suggests that this small number is sufficient to assess safety without subjecting too many individuals to the surgery. I strongly disagree, the fetal cell transplant studies seemed perfectly safe and HIGHLY effective in small open-label cohorts. The problem of GID emerged in the larger blinded trials that followed. In undertaking a small, open-label study, the authors are merely repeating the mistakes of the past. I would urge them to consider a larger cohort of 15-20 patients and independent blinded evaluations performed by qualified, disinterested parties.

4. Despite the approvals, I remain concerned about the ethics of these transplantation studies. All are targeting patients who are candidates for DBS and asking those patients to forego that standard of care, which has a 30-year track record of safety and efficacy, that requires just one trajectory per side, no immunosuppression, and improves symptoms within weeks of surgery, not months or years. This does not seem to be the choice a rational person would make unless they had a false hope of cure.

**Reviewer**	**2**
**Name**	**Jarraya, Bechir**
**Affiliation**	**Hopital Foch, Neurology**
**Date**	**17-Sep-2025**
**COI**	**None**

This trial aims to revive cell therapy for Parkinson's disease (PD) after a long history of unmet objectives, including during studies led by the authors, who are notable figures in this approach. The novelty here is shifting from fetal mesencephalic tissue to human ES cell-derived dopaminergic cells (hES-DA cells). This marks a significant paradigm shift, as using

hES-DA cells enables better optimization of cell therapy logistics and may help address graft-induced dyskinesias (which still need to be evidenced).

However, there are several points the authors should address.

1. The Rationale of the Cell Therapy Approach

It is now established that PD is an alpha-synucleinopathy, in which aggregated alpha-synuclein (aSyn) spreads beyond dopaminergic cells. Dopaminergic replacement helps only with dopamine-sensitive symptoms, excluding most non-motor symptoms. Therefore, even if the hES-DA approach ultimately succeeds (unlike previous fetal tissue methods by the same authors), it will only alleviate part of the symptoms. The authors mention gene therapy approaches for dopamine replacement (ProSavin/OXB-102). What are the advantages of hES-DA over dopamine gene therapy? Cell therapy has drawbacks, particularly the need for immunosuppressive treatment, which carries risks. In contrast, gene therapy does not require immunosuppression.

2. Concerns from Previous Studies

Previous research indicates that cell therapies in PD can lead to the spread of aggregated aSyn into grafted cells, causing their degeneration (Kordower et al.; publications from the current authors). How will the authors prevent this cell-to-cell transfer and the seeding of alpha-synucleinopathy? The authors should extensively discuss this critical point.

3. Treatment Delivery via Neurosurgical Stereotaxy

First, patients from the UK will need to travel to Sweden for surgical delivery, raising ethical concerns given the poor motor condition of PD patients considered for neurosurgery. Many neurosurgical centers performing deep brain stimulation (another PD treatment) are aware of the challenges involved in treating international patients. The authors should comment on how they will consider reducing the "travel effect".

Second, the operative procedure should be described in more detail (stereotaxy landmarks, etc.).

Third, it is unfortunate that the authors plan to use an investigational device (not CE marked) for cell delivery while simultaneously evaluating a new cell therapy. This could introduce confounding factors in the interpretation of the final results.

VERSION 1 - AUTHOR RESPONSE

Reviewer: 1
Dr. Ron L Alterman, Beth Israel Deaconess Medical Center
Comments to the Author:
The authors present the protocol for a planned Phase I, open-label safety and feasibility study of allogeneic stem cell transplantation in patients with moderately advanced Parkinson's disease (PD). Patients will be recruited from two centers (Cambridge, UK and Lund, Sweden) but will undergo surgery only at Lund due to the lack of a CE mark for the transplantation device. Eight patients with moderately advanced PD will be recruited and divided into two cohorts of four patients each. The first cohort will receive 3.54 x 106 cells in each Putamen and the second cohort will receive either or 7.08 x 106 or 3.54 x 106 cells. The decision regarding whether to proceed with the dose escalation will be made after the first cohort has been followed for 6 months. Following

transplantation patients will be treated with immunosuppressive medications for 12 moths. Standard inclusion and exclusion criteria are proposed as are standard primary and secondary outcome measures. Ethics boards at both institutions have approved the protocol.

We thank the reviewer for assessing our manuscript and the helpful comments.

This protocol description meets the criteria for publication in BMJ Open. I do have the following criticisms/comments:

1. I'm not sure how the authors can claim that this is a first in human trial given that Tabar, et al have already published a first in human trial of allogeneic embryonic stem-cell derived dopaminergic neurons (Nature; May 2025). How is this different?

This is a good point. This trial is the first in Europe using this approach and the first-in-human trial using this specific ESC-derived cell product (the STEM-PD product), derived from the RC17 cell line, which is different from the ESC-derived cell product used in Tabar et al., 2025. We have made this clearer on page 3:

This is first such trial using this approach in Europe and the first to use this human embryonic stem cell line, but a similar approach has been undertaken by other groups who have recently published the results from their first-in-human trials.

2. A more detailed description of the implant surgery is warranted, particularly how many trajectories per side and how many deposits per trajectory.

We thank the reviewer for this comment and have updated the methods to include the following paragraph on page 4 about the surgical technique:

The surgical procedure will use a stereotactic image-guided technique. Five targets for each putamen will be used, two in the anterior and three in the posterior part of the putamen, divided as follows: one anterior to the anterior commissure; one at the anterior commissural line; and three posterior to the anterior commissure. The trajectories to those targets for each putamen will be made through one or more burr holes per side of the cranium, depending on the best trajectory to the target, and avoiding the ventricles and blood vessels, which will be imaged preoperatively using MRI. The trajectories will be placed to achieve maximal distribution of the graft tissue within the putamen. For Dose 1 each tract will have 4 deposits, and for Dose 2 each tract will have 8 deposits. Each deposit will contain 2.5 µl of the cell suspension (70 000 cells/µl).

3. The authors' claim that their choice of study cohort was based on prior research, which suggests that this small number is sufficient to assess safety without subjecting too many individuals to the surgery. I strongly disagree, the fetal cell transplant studies seemed perfectly safe and HIGHLY effective in small open-label cohorts. The problem of GID emerged in the larger blinded trials that followed. In undertaking a small, open-label study, the authors are merely repeating the mistakes of the past. I would urge them to consider a larger cohort of 15-20 patients and independent blinded evaluations performed by qualified, disinterested parties.

We appreciate the concern of the referee and the fact that in the previous fetal human ventral mesencephalic transplant trials, graft-induced dyskinesias (GIDs) were only noticed in the double blind placebo control trials, although once recognised they were seen and reported in the smaller open label studies (Hagell et al., Nature Neuroscience 2002). These side effects were thought to be due to contaminating 5HT neurons in the fetal preparations which should not be found in stem cell-derived dopamine products (Kirkeby et al., Cell Stem Cell 2023). Thus, we think this side effect is unlikely to be seen but we will explicitly be looking for this as we did in the recent TransEuro study where they were again noted (Barker et al., Nat Biotechnol. 2025), even in a sample size of 11.

In terms of deciding on the sample size for first in human studies, it is always difficult to know what is the optimal number especially as this study is primarily concerned about safety. Larger cohorts will only be appropriate in subsequent studies where efficacy is being looked at. The number of patients being grafted in this study is not only similar to that reported in the published Japanese trial using human iPSC derived dopamine cells (Sawamoto et al. Nature 2025) but has been agreed upon after lengthy discussion with the regulatory agencies in Sweden and UK (MPA and MHRA). We have clarified this point on page 8 with the following addition:

"*This cohort size is similar to the number of patients that been grafted in a recently published Japanese trial using iPSC-derived dopamine cells and was agreed upon after detailed discussions with the regulatory agencies in Sweden and UK (MPA and MHRA)[16]. Furthermore, trials of this size have been shown previously to identify specific side effects, including graft-induced dyskinesias*"

Future phase 2 studies will likely adopt a blinded approach in a larger number of patients. Finally, all patients in this study (as with our previous TransEuro trial) have their assessments videoed so that third parties could review these if needed. This is clarified with the addition on page 6:

"The motor assessments will be video recorded such they can be rated by a blinded independent rater, as was done in the TransEuro fetal dopamine cell transplant trial."

4. Despite the approvals, I remain concerned about the ethics of these transplantation studies. All are targeting patients who are candidates for DBS and asking those patients to forego that standard of care, which has a 30-year track record of safety and efficacy, that requires just one trajectory per side, no immunosuppression, and improves symptoms within weeks of surgery, not months or years. This does not seem to be the choice a rational person would make unless they had a false hope of cure.

We thank the referee for the comment and concerns expressed. This is a first-in-human trial of a new therapeutic approach using this cell therapy for Parkinson's disease, for which we have received both ethical and regulatory approval from the relevant agencies in Sweden and UK. This therapy holds the hope not only for symptomatic improvement but long-lasting repair and restoration of one area of cell loss in PD, while not offering a cure. This is clearly explained to the patient along with the experimental nature of this intervention which will include letting them know about the risks of the procedure and the fact that they may get worse or not improve from the intervention.

It is also important to recognise that the stage of disease being targeted for recruitment to this trial has been extensively discussed with the national authorities and the ethical boards in both the UK and Sweden. This disease stage is comparable to the cohorts included in the recently published studies using similar approaches (Tabar et al., 2025; Sawamoto et al., 2025). As is expected by our ethical committees, all patients will be advised and offered standard of care treatments, including DBS, as is normal for studies of this type. The experimental nature of this intervention will be explicitly emphasised and discussed. Furthermore, regarding DBS, some patients in the historical fetal grafting studies went onto to receive DBS surgery and a history of cell replacement therapy was not a barrier to this later intervention. Thus, patients will be fully informed of their treatment options. Furthermore, all patients have to have normal cognition and capacity in order to consent for this intervention, which we would deem to make their decision rational. We have expanded on the ethics section of the manuscript on page 8 to explain this:

"*Potential participants will receive verbal and written information about the trial and these will follow Good Clinical Practice (GCP), local regulatory and legal requirements, and General Data Protection Regulation (GDPR). The investigator or designee will ensure that each trial participant is fully informed about the nature and objectives of the trial and possible risks associated with their participation as well as the alternative therapies that are available for treating Parkinson's at this stage of the condition. All patient information and consent documentations have been reviewed by the ethical review bodies in both countries and are provided in Supplementary File 1."*

Reviewer: 2
Prof. Bechir Jarraya, Hopital Foch
Comments to the Author:
This trial aims to revive cell therapy for Parkinson's disease (PD) after a long history of unmet objectives, including during studies led by the authors, who are notable figures in this approach. The novelty here is shifting from fetal mesencephalic tissue to human ES cell-derived dopaminergic cells (hES-DA cells). This marks a significant paradigm shift, as using hES-DA cells enables better optimization of cell therapy logistics and may help address graft-induced dyskinesias (which still need to be evidenced).

We thank the referee for reviewing our manuscript and the helpful comments.

However, there are several points the authors should address.

1. The Rationale of the Cell Therapy Approach
It is now established that PD is an alpha-synucleinopathy, in which aggregated alpha-synuclein (aSyn) spreads beyond dopaminergic cells. Dopaminergic replacement helps only with dopamine-sensitive symptoms, excluding most non-motor symptoms. Therefore, even if the hES-DA approach ultimately succeeds (unlike previous fetal tissue methods by the same authors), it will only alleviate part of the symptoms. The authors mention gene therapy approaches for dopamine replacement (ProSavin/OXB-102). What are the advantages of hES-DA over dopamine gene therapy? Cell therapy has drawbacks, particularly the need for immunosuppressive treatment, which carries risks. In contrast, gene therapy does not require immunosuppression.

We thank the referee for this comment. We agree that dopaminergic cell replacement targets only dopamine-responsive symptoms and that problems in PD that have an origin outside this system will not be helped by this intervention. This will include non-dopaminergic motor problems (e.g. gait) and non-dopaminergic, non-motor features. A thorough discussion of the rationale and the merits of cell replacement will be explained to the patients, along with the risks and limitations of this dopamine cell therapy approach. However, the current manuscript is merely reporting the study protocol as is customary. The merits, limitations and risks of this approach compared to other experimental interventions such as gene therapies lies outside the scope of this submitted paper, but relevant papers discussing this have now been referenced in the revised manuscript introduction (page 3).

Thus, there is an urgent need for better treatments of Parkinson's and a number of experimental approaches are being considered including gene therapies, immune therapies, small molecules and drug repurposing approaches[4].

2. Concerns from Previous Studies
Previous research indicates that cell therapies in PD can lead to the spread of aggregated aSyn into grafted cells, causing their degeneration (Kordower et al.; publications from the current authors). How will the authors prevent this cell-to-cell transfer and the seeding of alpha-synucleinopathy? The authors should extensively discuss this critical point.

We thank the referee for this comment that is addressing an important question in the field. Of note, the alpha-synuclein inclusions that were reported in post-mortem tissue occurred > 10 years after fetal human VM cell transplant implantation. These were only seen in a small proportion of cells and there was no indication that this significantly affected graft function. Whether human embryonic-derived stem cells grafts are also vulnerable to the spread of aggregated alpha-synuclein pathology in the human Parkinsonian brain is unknown. However, this manuscript is discussing the trial protocol and thus issues around graft pathology are merely referenced in line with the concern around the competitive advantage of dopamine cell therapies in the treatment of advancing Parkinson's (see above response)

3. Treatment Delivery via Neurosurgical Stereotaxy
First, patients from the UK will need to travel to Sweden for surgical delivery, raising ethical concerns given the poor motor condition of PD patients considered for neurosurgery. Many neurosurgical centers performing deep brain stimulation (another PD treatment) are aware of the challenges involved in treating international patients. The authors should comment on how they will consider reducing the "travel effect".
Second, the operative procedure should be described in more detail (stereotaxy landmarks, etc.).
Third, it is unfortunate that the authors plan to use an investigational device (not CE marked) for cell delivery while simultaneously evaluating a new cell therapy. This could introduce confounding factors in the interpretation of the final results.

We thank the referee for this comment.

The patients will travel in the ON state to Lund, Sweden with a medical practitioner from the UK and a care giver. The patients chosen for this study are at a stage of disease, where they can function near normally when ON and so should be able to manage the travel. This has been discussed and approved by the relevant ethical committees.

Whilst several surgical sites would make it easier for patients in terms of their travel, one surgical site will reduce any potential variability due to a site/neurosurgeon effect. However, the more fundamental issue in our trial is that the device we are using is not CE marked and so can only be used in the hospital in Lund, and thus we have no choice but to ensure all patients come to this one surgical site. The reason for choosing this particular device is that there is no licensed system for delivering cells to the human brain. Given this we have chosen to use this particular instrument, because we know it can deliver cells successfully to the human brain with no safety risks based on its previous use in ~20 people with Parkinson's over 30 years using human fetal dopamine cells.

For this particular trial, we have therefore explicitly said that the inclusion criteria includes an assessment for "Ability to travel to Lund for surgery" (Table 1). We have made an addition on page 4 to explain this and that participants travelling for surgery will be accompanied by a member of the UK trial team as below in order to ease the burden of travel:

"This device has been shown to deliver developing dopamine cells accurately to the target site - the putamen [18] - and so we deemed it to be the best device to use in this trial given no approved and licensed device is available for cell delivery to the CNS. Given this, all UK participants will have to travel to Sweden and, to help support this, they will be accompanied by a care giver and a medically qualified member of the UK trial team. Furthermore, their stage of disease has been chosen (after discussions with the ethical committees) such that they are fully ambulant when on medication so they will be able to undertake this travel. This is part of the inclusion criteria."

We have also included more detail on the surgical procedure in the methods on page 4 as below.

"The surgical procedure will use a stereotactic image-guided technique. Five targets for each putamen will be used, two in the anterior part and three in the posterior part of the putamen, divided as follows: one anterior to the anterior commissure; one at the anterior commissural line; and three posterior to the anterior commissure. The trajectories to those targets for each putamen will be made through one or more burr holes per side of the cranium, depending on the best trajectory to the target, and avoiding the ventricles and blood vessels, which will be imaged preoperatively using MRI. The trajectories will be placed to achieve maximal distribution of the graft tissue within the putamen. For Dose 1 each tract will have 4 deposits, and for Dose 2 each tract will have 8 deposits. Each deposit will contain 2.5 µl of cell suspension (70 000 cells/µl)."

VERSION 2 - REVIEW

**Reviewer**	**1**
**Name**	**Alterman, Ron L**
**Affiliation**	**Beth Israel Deaconess Medical Center, Neurosurgery**
**Date**	**13-Nov-2025**
**COI**	

I thank the authors for addressing my concerns. Two remain:

1- You state in the text that candidates will be apprised of alternative approved therapies that are available to them, but I see no mention of alternative therapies in the otherwise highly detailed consent document. Did I miss it?

2- Curious as to why there is a pre-commissural tract. Prior biosurgical therapies have only targeted the post-commissural putamen as that was the 'motor' region receiving the nigral projections.

**Reviewer**	**2**
**Name**	**Jarraya, Bechir**
**Affiliation**	**Hopital Foch, Neurology**
**Date**	**12-Nov-2025**
**COI**	

I want to thank the authors for the revision.

Now I have no further concerns about this very well-written paper and important study.

I wish them all the best for their clinical trial.

VERSION 2 - AUTHOR RESPONSE

Reviewer: 2
Prof. Bechir Jarraya, Hopital Foch
Comments to the Author:
I want to thank the authors for the revision.
Now I have no further concerns about this very well-written paper and important study.
I wish them all the best for their clinical trial.

Thank you for your comments and for your time in reviewing our manuscript.

Reviewer: 1
Dr. Ron L Alterman, Beth Israel Deaconess Medical Center
Comments to the Author:
I thank the authors for addressing my concerns. Two remain:

Thank you for your comments and for your time in reviewing our manuscript.

1- You state in the text that candidates will be apprised of alternative approved therapies that are available to them, but I see no mention of alternative therapies in the otherwise highly detailed consent document. Did I miss it?

Thank you for your comment. Alternative treatments are not detailed in the consent documents as all the patients eligible and recruited for this trial are already on the standard of care for Parkinson’s disease for their stage of disease. Patients are not asked to forgo any treatment in order to participate in this trial. To make this clearer we have removed the reference to alternative the line about alternative therapies on page 8 and have made the following addition:

All patients remain on the standard of care for the treatment of Parkinson’s disease, under the care of their treating neurologist during the course of the trial.

2- Curious as to why there is a pre-commissural tract. Prior biosurgical therapies have only targeted the post-commissural putamen as that was the 'motor' region receiving the nigral projections.

Thank you for your comment. The surgical route chosen is in line with the safe approach used previously in the TransEuro trial, as well as similar approaches in recent stem-cell derived dopamine graft trials such as in Sawamoto et al. (2025).
